# *Porphyromonas gingivalis* sphingolipids affect early responses of THP-1 macrophages to outer membrane vesicles

**DOI:** 10.1128/iai.00736-25

**Published:** 2026-07-06

**Authors:** Zavier G. Eure, Tamires Fernanda Vilas Boas Cordeiro, Alejandro Riveros Walker, Fernanda G. Rocha, Mary Ellen Davey, Frank C. Gibson

**Affiliations:** 1 Department of Oral Biology, College of Dentistry, University of Florida 3463, Gainesville, Florida, USA; 2 Department of Periodontology, College of Dentistry, University of Florida 3463, Gainesville, Florida, USA; 3 Department of Microbiology, ADA Forsyth Institute 1816, Somerville, Massachusetts, USA; University of Pennsylvania School of Veterinary Medicine, Philadelphia, Pennsylvania, USA

**Keywords:** transcriptome, macrophage, innate immune sensing, host-pathogen interaction, outer membrane vesicles, sphingolipids, *Porphyromonas gingivalis*

## Abstract

*Porphyromonas gingivalis* is an oral bacterium commonly associated with periodontitis. *P. gingivalis* synthesizes sphingolipids (SLs), and recently, we reported that *P. gingivalis* SLs packaged into outer membrane vesicles (OMVs) can regulate THP-1 macrophage inflammatory responses to *P. gingivalis* OMVs. The contribution that *P. gingivalis* SLs have on host-pathogen interactions remains poorly understood, especially in the context of OMVs. Here, we demonstrate that *P. gingivalis* SLs significantly reduce the uptake of OMVs isolated from SL-containing wild-type (WT) *P. gingivalis* compared to OMVs from an SL-null mutant *P. gingivalis* strain, and that the loss of SLs drives uptake of *P. gingivalis* OMVs via lipid rafts in THP-1 macrophages. Intriguingly, we found that the sensing of *P. gingivalis* OMVs via TLR2 and MyD88 is attenuated due to the presence of SLs. Lastly, transcriptomic analysis of THP-1 macrophages co-cultured with either WT or SL-null *P. gingivalis* OMVs for 2 h revealed an array of differentially expressed genes. Interestingly, at 2 h, WT *P. gingivalis* OMVs promoted an overall trend of gene downregulation in THP-1 macrophages when compared to unchallenged, whereas SL-null OMVs strongly promoted upregulated gene expression. These findings provide early-phase characterization of the role of SLs in initial host cellular responses to *P. gingivalis* OMVs and expand our knowledge of interactions between SL-containing *P. gingivalis* OMVs and the host.

## INTRODUCTION

Sphingolipids (SLs) are a diverse class of membrane phospholipids composed of a sphingoid long-amino alcohol chain, fatty acid, and variable lipid head group (1). SLs are *de novo* synthesized by the enzyme serine palmitoyltransferase (SPT) that catalyzes the first step in the pathway. SL production is nearly ubiquitous among eukaryotes, and these lipids play important roles in multiple cellular processes such as stress response, cell signaling, and inflammation (1). Contrary to eukaryotes, SL synthesis is relatively uncommon among prokaryotes, with only approximately 100 bacterial phyla, including *Proteobacteria*, *Chlorobi*, and the *Bacteroidota,* known to synthesize SLs (2). Our functional understanding of the contributions of microbial SLs to bacteria is much less understood than their eukaryotic counterparts. Interestingly, work with the gut commensal *Bacteroides thetaiotaomicron* shows that the SLs of this organism are detected in intestinal tissues, and their presence in these tissues preserves intestinal health (3). Outside the gut, other sites, including the oral cavity, are known to harbor microbial SLs. Work by Nichols et al. (4) reported the identification of and shifts in microbial SLs present in gingival tissues of healthy individuals and those with periodontal disease, supporting that microbial SLs play an important role in periodontal disease progression.

*Porphyromonas gingivalis* is a gram-negative oral bacterium of the phylum *Bacteroidota*. This organism is closely associated with the highly prevalent chronic oral inflammatory disease known as periodontitis. Periodontitis affects approximately 47% of adults over the age of 30 (5) and is characterized by immune dysregulation driven by a polymicrobial dysbiosis of the subgingival biofilm. The resulting exacerbated host inflammatory response leads to irreversible destruction of the periodontal soft tissues and progressive alveolar bone resorption, ultimately contributing to tooth loss (6). Current therapy relies primarily on mechanical disruption of subgingival biofilm through scaling and root planing. Other adjuvant treatments can also be used, such as host immunomodulation therapies (7), offering additional potential benefits to limit tissue destruction. Nevertheless, these approaches are not sufficient to eradicate the disease. Although the precise mechanisms that initiate and sustain periodontitis remain unclear, the disease is recognized as a risk factor for diabetes mellitus and has also been associated with several systemic conditions, including rheumatoid arthritis, cardiovascular diseases, respiratory diseases, inflammatory bowel disease, among others (8). Interestingly, *P. gingivalis* or its products can translocate from the oral cavity to distant sites and have been detected in these extra-oral sites (6, 9–12). Similar to other members of the *Bacteroidota* phylum, *P. gingivalis* is capable of *de novo* SL biosynthesis (13–15). Employing purified SLs from *P. gingivalis* such as Lipid 1256 and Lipid 430 identified that these lipids can elevate inflammatory responses in host cells (16, 17), although how SLs contribute to the inflammatory response to *P. gingivalis* in the context of a live organism was unknown at that time. Following identification, an initial characterization of the SPT gene of *P. gingivalis* responsible for encoding the key enzyme required for SL biosynthesis, SPT (15), it was found using various immune cells that in the context of live organisms, *P. gingivalis* SLs limit host inflammatory responses to this organism (13, 18).

An interesting feature of *P. gingivalis* is its ability to package and secrete its virulence factors into nanosized spherical proteoliposomes known as outer membrane vesicles (OMVs) (19). *P. gingivalis* OMVs are loaded with internal and external cargo elements such as novel lipids (amino lipids and SLs), lipopolysaccharide (LPS), outer membrane and periplasmic proteins, and nucleic acids (20). Interestingly, it has been shown that there is both diversity among *P. gingivalis* OMVs in both size and cargo (21, 22). Although we do not fully understand all the benefits OMVs provide to *P. gingivalis,* it is known that OMV production by other bacteria is involved in several key processes, including nutrient acquisition, stress response, and cellular communication between host cells and other microbes (19, 23, 24). Recently, our group extended our understanding of *P. gingivalis* OMVs by identifying these structures as a delivery platform that *P. gingivalis* utilizes to transfer its own SLs to host cells, therefore limiting the host inflammatory responses to these OMVs. This process is similar to what was found to happen with whole organisms, thus supporting that SLs or the SL-associated cargo of *P. gingivalis* OMVs functionally regulate host inflammation (25), although precisely how *P. gingivalis* OMVs interact with the host is unclear.

Sensing of *P. gingivalis* OMVs by host cells involves Toll-like receptors (TLRs), especially TLR2 and TLR4 (26). These findings are consistent with results from *in vitro* live *P. gingivalis* bacterial challenge studies and animal modeling implicating the importance of TLR2 and TLR4 in the sensing of this organism (27–31). Beyond sensing, the uptake of *P. gingivalis* OMVs by host cells has primarily been studied using cells of epithelial origin, and to date, the mechanism of uptake of *P. gingivalis* OMVs appears to be cell nuanced, as several mechanisms have been described. For example, *P. gingivalis* OMV uptake appears to occur in a caveolin-mediated lipid raft endocytic process by HeLa cells (32), while a lipid raft-dependent endocytic pathway independent of caveolin was reported using human gingival epithelial cells (33), and when employing SY5Y human neuroblastoma and BV-2 mouse microglial cells, uptake of *P. gingivalis* OMVs was found to involve clathrin-dependent endocytosis (34). Macrophages have been shown to play a key role in sensing *P. gingivalis* (35) and are required for *P. gingivalis* to drive oral bone loss (36). However, there is limited information on bacterial OMV uptake via macrophages, and it is not understood how *P. gingivalis* OMVs are taken up by these cells and the impact of *P. gingivalis* SLs on the uptake of *P. gingivalis* OMVs.

Herein, we report that during the early events of the host response, *P. gingivalis* SLs incorporated in OMVs reduce OMV uptake by THP-1 macrophages. We also report that the absence of *P. gingivalis* SLs promotes strong lipid-raft-dependent endocytosis of OMVs via THP-1 macrophages. Furthermore, we found that immune sensing of *P. gingivalis* OMVs occurs via the TLR2 and myeloid differentiation primary response 88 (MyD88) axis. Lastly, transcriptomics of THP-1 cells co-cultured with wild-type (WT) OMVs for 2 h identified suppressed gene and pathway expression, while SL-null OMVs activated gene and pathway expression. Overall, our findings identify novel contributions of SLs to the interaction between *P. gingivalis* and the host via OMVs.

## RESULTS

### Lipophilic dye labeling does not affect *P. gingivalis* OMV size, composition, and elicited inflammation

**Fig 1:**
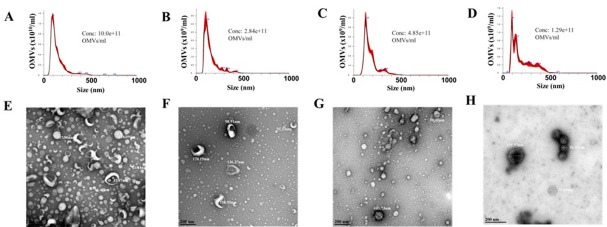
Characterization of *P. gingivalis* OMVs using NTA and negative-stain TEM. 1 × 10^11^ *P. gingivalis* W83 WT (**A and B**) and SL-null (ΔSPT; **C and D**) OMVs were either unlabeled (**A and C**) or labeled with DiO lipophilic dye (**B and D**) and analyzed by NTA to characterize OMV size (*x*-axis) and relative concentration (*y*-axis). Negative staining TEM was performed to assess ultrastructure of the corresponding OMV samples before (**E and G**) and after (**F and H**) DiO labeling; scale bar = 200 nm.

We found, using nanoparticle tracking analysis (NTA), that our isolated and purified OMVs from SL-containing WT *P. gingivalis* and *P. gingivalis* SPT mutant (SL-null) strains displayed physical characteristics similar to what we previously reported (25). As multiple studies have used lipophilic dye staining to monitor bacterial OMV uptake and trafficking by fluorescent microscopy (37–39), we first characterized the impact of DiO labeling on *P. gingivalis* OMVs, as prior reports suggest that lipophilic dye labeling can impact the biological activity of OMVs (40, 41). Using NTA, we found the average OMV size and distribution did not change between WT and SL-null OMVs due to the labeling process (Fig. 1A through D). We confirmed via negative stain-transmission electron microscopy (TEM) that unlabeled WT and SL-null OMVs have similar morphology (Fig. 1E and G). In addition, we found that the OMV membrane appeared intact after labeling and resembled unlabeled OMVs for both WT and SL-null OMVs (Fig. 1E through H). To understand if labeling of OMVs caused functional changes in the way host cells responded to labeled OMVs, we cultured THP-1 macrophages with unlabeled and labeled *P. gingivalis* OMVs and performed multiplex immunoassays on the culture supernatant fluids. When comparing the cytokine and chemokine profiles of THP-1 macrophages cultured with unlabeled and DiO-labeled *P. gingivalis* OMVs, DiO labeling had a minor impact on SL-null OMV cytokine or chemokine activity (*P* > 0.05 for all), as well as for WT *P. gingivalis* OMVs (*P* > 0.05), with the exception of a modest change in TNF-α (*P* < 0.05) (Fig. S1). Taken together, these data support that DiO labeling did not alter *P. gingivalis* W83 WT or SL-null OMVs and supported their use in OMV uptake assays.

### *P. gingivalis* sphingolipids limit OMV uptake by THP-1 macrophages

**Fig 2:**
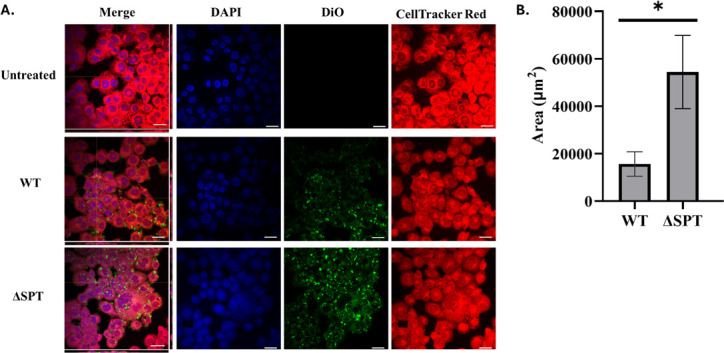
*P. gingivalis* OMV uptake by THP-1 macrophages is reduced by SLs. (**A**) Top to bottom: THP-1 macrophages were either untreated or challenged with DiO-labeled *P. gingivalis* W83 WT (WT) or SL-null (ΔSPT) OMVs at 1,000 OMVs/cell for 2 h. 3-D Z-stack images of representative fields were taken at 600× magnification and converted to 2-D HDR images. Left to right: merged, DAPI only (nuclei; blue), DiO only (OMVs; green), and CellTracker Red only (membrane; red), scale bar = 25 µm. Merged images for each condition show XZ (bottom panel) and YZ (right side panel) orthogonal views. (**B**) Regions of interest (ROIs) were traced around the perimeter of 250 individual cells, and the sum area of internalized OMVs (green; µm^2^) of each ROI was measured. Bars represent mean ± SEM values, *n* = 5 independent experiments. Data were analyzed by unpaired *t*-test and * = *P* < 0.05.

Several studies have found that bacterial OMVs can have a profound effect on host cellular responses (i.e., proliferation and inflammation) (42–44). Yet despite these findings, there is a gap in knowledge on how OMVs are initially taken up by macrophages. The uptake of OMVs is known to be impacted by the various cargo elements that make up its composition, such as outer membrane proteins, bacterial toxins, peptidoglycan, nucleic acids such as siRNA and DNA, and bacterial lipids such as LPS (45). To understand the effect SLs have on *P. gingivalis* OMV uptake, we challenged THP-1 cells with DiO-labeled *P. gingivalis* WT and SL-null OMVs and measured OMV uptake by confocal fluorescence microscopy. We observed that there was significantly more uptake of SL-null OMVs (mean = 54,432 ± 15,437 µm^2^) compared to WT OMVs (mean = 15,635 ± 5,170 µm^2^) (*P* < 0.05; Fig. 2A and B). These data support that *P. gingivalis* SLs limit OMV uptake by THP-1 macrophages.

### Sphingolipid status impacts OMV uptake via lipid rafts

**Fig 3:**
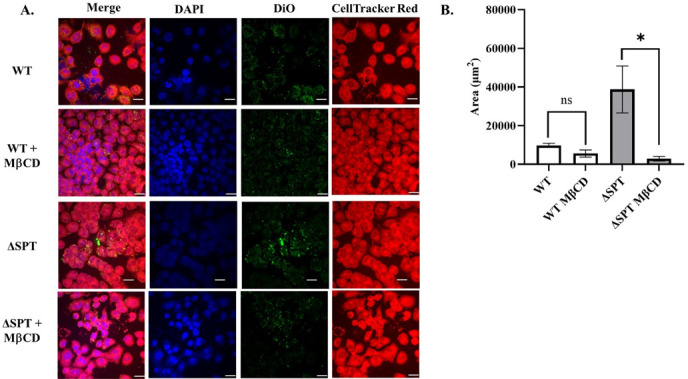
Uptake of *P. gingivalis* SL-null OMVs is dependent upon lipid rafts. (**A**) Top to bottom: THP-1 macrophages were either untreated or treated with the cholesterol sequestration agent MβCD (10 mM) to disrupt lipid rafts and were then challenged with DiO-labeled WT *P. gingivalis* OMVs (WT) or SL-null OMVs (ΔSPT) at 1,000 OMVs/cell for 2 h. 3-D Z-stack images of representative fields were taken at 600× magnification and converted to 2-D HDR images. Left to right are merged, DAPI only (nuclei; blue), DiO only (OMVs; green), and CellTracker Red only (membrane; red), scale bar = 25 µm. (**B**) ROIs were traced around the perimeter of 250 individual cells, and the sum area of internalized OMVs (green; µm^2^) of each ROI was measured. Bars represent mean ± SEM values, *n* = 5 independent experiments. Data were analyzed by unpaired *t*-test; ns, not significant; * = *P* < 0.05.

Previous studies investigating WT *P. gingivalis* OMV uptake have found that lipid rafts played a role in that process (32–34). Furthermore, these studies have primarily focused on epithelial cells (32, 33). Currently, it is not known how phagocytic cells such as macrophages take up *P. gingivalis* OMVs, and what the contribution SLs have on this uptake mechanism. Using methyl-β-cyclodextrin (MβCD) to disrupt THP-1 cell lipid rafts before OMV challenge, we first optimized the concentration of MβCD needed to sequester cholesterol from the THP-1 cell membrane and found that 10 mM MβCD maximally depleted cholesterol without impacting THP-1 cell viability (Fig. S2 and S3). Employing this condition, we found that MβCD treatment of THP-1 macrophages did not significantly reduce WT *P. gingivalis* OMV uptake compared to cells that did not receive MβCD treatment (*P* = 0.8873, Fig. 3). Interestingly, we observed MβCD treatment profoundly reduced THP-1 cell uptake of SL-null OMVs (*P* < 0.05, Fig. 3). These data support that while *P. gingivalis* WT OMVs are not taken up by a mechanism that involves lipid rafts, SL-null OMV uptake clearly involved lipid rafts, thus the bacterial SLs play an important role in the way THP-1 macrophages take up *P. gingivalis* OMVs. As the route of OMV uptake is known to impact elicited inflammatory responses from challenged host cells (46, 47), we also investigated the effect of lipid raft disruption on *P. gingivalis* OMV-elicited inflammation after MβCD inhibition. Regardless of the cytokines and chemokines examined, there were no significant differences found between inhibitor-treated or untreated THP-1 cells in their response to WT or SL-null OMVs (Fig. S4). These data support that WT *P. gingivalis* OMV-elicited inflammation occurs independently of lipid rafts in THP-1 macrophages.

### SLs alter TLR- and MyD88-dependent innate immune sensing of *P. gingivalis* OMVs

**Fig 4:**
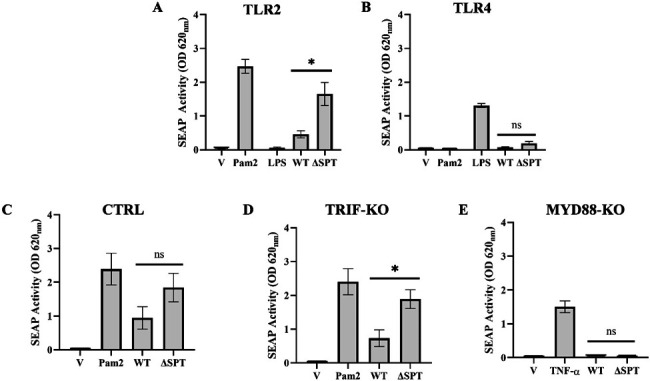
The SLs of *P. gingivalis* influence TLR sensing of its OMVs. HEK-Blue cells: hTLR2 (**A**) and hTLR4 (**B**), as well as THP-1 Dual cells: CTRL (**C**), TRIF-KO (**D**), and MyD88-KO (**E**) cells were challenged with *P. gingivalis* W83 OMVs from WT or SL-null (ΔSPT) at 1,000 OMVs/cell. Pam2CSK4 (Pam2) served as TLR2 positive control for HEK-Blue cells and as a control for MyD88-dependent THP-1 Dual cells. UltraPure *E. coli* LPS (LPS) served as TLR4 positive control. Recombinant TNF-α served as a MyD88-independent positive control. Vehicle alone (V)-treated cells served as a baseline. SEAP levels were measured spectrophotometrically at 620 nm. Bars represent mean ± SEM values, *n* = 3–5 independent experiments. Data were analyzed by unpaired *t*-test; ns, not significant; * = *P* < 0.05.

As it is known that *P. gingivalis* OMVs can activate cells in both a TLR2- and TLR4-dependent manner (26), we cultured HEK-Blue hTLR2 and hTLR4 reporter cells with WT and SL-null OMVs to investigate the contribution of SLs to innate immune sensing of *P. gingivalis* OMVs via TLRs. As expected, our positive controls, Pam2CSK4 (Pam2; TLR2) and ultra-pure *Escherichia coli* LPS (TLR4), were specific (Fig. 4). Interestingly, SL-null OMVs elicited a much stronger signaling phenotype than was observed with WT OMVs (Fig. 4A). Employing TLR4 reporter cells, we observed that there were no significant differences between OMV treatments (*P* > 0.05, Fig. 4B).

To further investigate the impact of SLs on TLR signaling, we utilized control THP-1 reporter cells as well as their TRIF-KO and MYD88-KO mutant cell lines to understand the importance of these adaptor molecules in the sensing of WT and SL-null OMVs. We found that control reporter cells possessing both MyD88 and TRIF responded less robustly to *P. gingivalis* WT OMVs compared to SL-null OMVs (Fig. 4C). Using TRIF-KO cells (MyD88-signaling intact), we found that there was a similar response profile to that observed with control reporter cells for WT and SL-null OMVs (Fig. 4D). Interestingly, when using the MYD88-KO cell line (TRIF-signaling intact), we found OMVs from both strains were only minimally sensed (Fig. 4E), thus supporting that MyD88 is a key signaling pathway adaptor molecule for TLR sensing of *P. gingivalis* OMVs.

### Transcriptomic analysis of THP-1 macrophages co-cultured with OMVs

**Fig 5:**
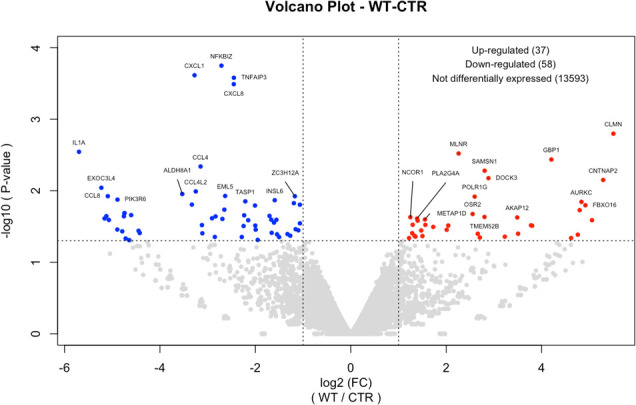
Volcano plot illustrating the transcriptome of THP-1 macrophages co-cultured with WT OMVs versus untreated THP-1 cells (CTRL). Differential expression analyses were performed with cutoffs for log_2_-fold change >1.0 and *P*-value <0.05. Upregulated genes are red and downregulated genes are blue.

**Fig 6:**
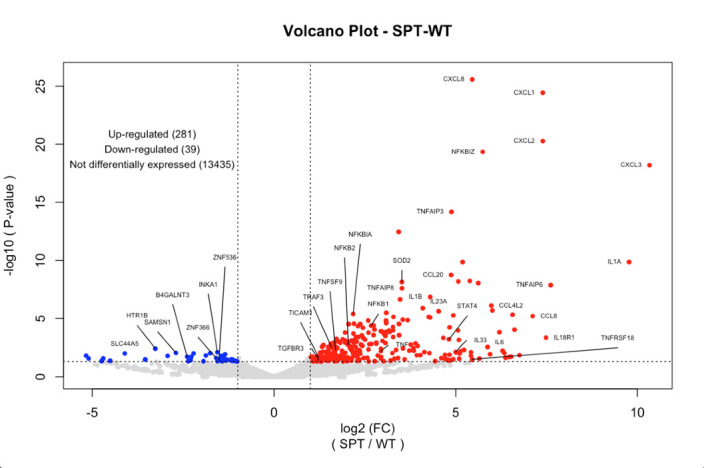
Volcano plot illustrating the transcriptome of THP-1 macrophages co-cultured with SL-null OMVs (SPT) versus THP-1 cells co-cultured with WT OMVs. Differential expression analyses were performed with cutoffs for log_2_-fold change >1.0 and *P*-value <0.05. Upregulated genes are red and downregulated genes are blue.

To understand the contribution of *P. gingivalis* OMV SLs in the host-pathogen interaction, we analyzed the transcriptome of THP-1 macrophages cultured with WT OMVs or SL-null OMVs for 2 h, and medium-only treatment served as control. Heatmap analysis showed striking differences between the treatments, while gene expression patterns of the biological replicates within each group were highly similar (Fig. S5). Both WT OMVs and SL-null OMVs elicited transcriptomic shifts in THP-1 macrophages versus THP-1 macrophages cultured in medium alone. Examining the data to identify differentially expressed genes (DEGs) elicited by *P. gingivalis* OMVs, we found 95 DEGs (37 upregulated and 58 downregulated) when comparing THP-1 macrophages co-cultured with WT *P. gingivalis* OMVs to cells cultured in medium alone (Fig. 5). When comparing SL-null OMV treatment to the untreated control, we found 260 DEGs (209 upregulated and 51 downregulated) (Fig. S6). Lastly, comparing SL-null OMVs challenge to WT OMVs, we observed 320 total DEGs (281 upregulated and 39 downregulated) (Fig. 6). The lists of all DEGs can be found in the supplementary materials (Table S1 to S6).

Among the DEGs found between THP-1 macrophages cultured with WT *P. gingivalis* OMVs compared with unchallenged cells, we unexpectedly observed more genes downregulated (61%) than upregulated (39%). Top downregulated genes include IL1A, EXOC3L4, POLN, CCL8, DNLZ, and PIK3R6, while top upregulated genes included CLMN, CNTNAP2, GABRB2, FBXO16, AURKC, CELF3, GBP1, and TFAP2C (Fig. 5, Table S4). Interestingly, when comparing THP-1 macrophages co-cultured with SL-null OMVs to THP-1 macrophages co-cultured with WT OMVs, we found 88% upregulated genes and 12% downregulated, with the top upregulated genes including EGR3, CXCL3, IL1A, TNFAIP6, CXCL2, and CXCL1, and the top downregulated genes including IGFLR1, ARHGAP40, CELF3, and TJP3 (Fig. 6). Finally, comparing SL-null OMV treatment to medium-only, we observed 80% of genes upregulated, including LAMP3, IL18AP, IL6, and CXCL3, while 20% were downregulated and included JAML, RASRF2, and NHLRC1 (Fig. S6).

### Pathways and gene ontology affected

**Fig 7:**
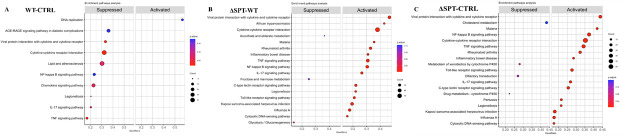
Enrichment pathway analysis of DEGs in THP-1 macrophages co-cultured with *P. gingivalis* OMVs. (**A**) THP-1 macrophages co-cultured with WT OMVs versus untreated (CTRL), (**B**) THP-1 macrophages co-cultured with SL-null (∆SPT) OMVs versus co-cultured with WT OMVs, (**C**) THP-1 macrophages co-cultured with ∆SPT OMVs versus CTRL. KEGG pathways are grouped into suppressed pathways (left) and activated pathways (right). The GeneRatio on the *x*-axis indicates the proportion of genes involved in each pathway. Circle size reflects the gene count associated with each pathway, and circle color represents the adjusted *P*-value (p.adjust), from red (significant), purple to blue (not significant).

To obtain a higher-order understanding of the gene expression profiles, we performed KEGG pathway analysis. At 2 h, when comparing WT to untreated cells, we found 12 enriched pathways that were differentially expressed. We found only 1 pathway activated (but not significant; *P* > 0.05), and similar to what was observed at the gene expression level, 11 pathways were found suppressed, with 7 being significantly different (*P* < 0.05). All those significantly suppressed genes relate to elements of host inflammatory responses including “cytokine-cytokine receptor interaction,” “chemokine signaling pathway,” “IL-17 signaling pathway,” and “TNF signaling pathway” (Fig. 7A). KEGG analysis of THP-1 macrophages cultured with SL-null OMVs versus WT OMVs revealed that 15 pathways were activated (*P* < 0.05 for all comparisons), including “viral protein interaction with chemokine and cytokine receptor,” “cytokine-cytokine receptor interaction,” “TNF signaling pathway,” “NF-kappa B signaling pathway,” “IL-17 signaling pathway,” “c-type lectin receptor signaling pathway,” and “Toll-like receptor signaling pathway,” while only three pathways were found mildly suppressed (not significant; *P* > 0.05) (Fig. 7B). Notably, when we compared the top up- and downregulated DEGs from the WT OMVs vs untreated THP-1 macrophages to the top activated or suppressed enriched KEGG pathways, we found that some downregulated genes were linked to “cytokine-cytokine receptor interaction” (IL1A, CCL8) and “chemokine signaling pathway” (CCL8, PIK3R6). We found that all of these pathways were suppressed in response to *P. gingivalis* OMVs (Fig. 7A and Table S7).

The transcription profile of top upregulated genes from the SL-null OMVs to WT OMVs challenge aligned with KEGG pathways: “Toll-like receptor signaling” (CCL8), “c-type lectin receptor signaling” (EGR3), “TNF signaling” (CXCL2, CXCL3, IL18R1), “IL-17 signaling pathway” (IL17C), “cytokine-cytokine receptor interaction” (IL1A, CCL8), and “NF-kappa B signaling pathway” (NFKB1, CXCL1). Fascinatingly, these pathways were significantly activated (Fig. 7B and Table S8), further supporting that SLs are strongly tied to the early immune response to *P. gingivalis* OMVs. The transcription profile comparing SL-null OMV-challenged THP-1 cells vs untreated THP-1 cells showed a similar transcriptional profile and the same activated KEGG pathways mentioned previously in our SL-null vs WT OMVs group (Fig. 7C and Table S9). Ultimately, these findings of the transcriptional landscape in the absence of SLs in *P. gingivalis* OMVs lead to an accentuated inflammatory response in host cells, supporting that *P. gingivalis* SLs dampen the host response in several areas related to host cellular response.

## DISCUSSION

The importance of microbial SLs in periodontal disease has been established (4, 48), but precisely how these lipids impact periodontal disease pathogenesis remains unclear. Although SL production by bacteria is rare, early work in this area focused on structural characterization of *P. gingivalis* SLs and characterization of host response to these lipids in pure form when cultured with host cells (14, 49–54). A seminal study by Moye et al. (15) identified the *P. gingivalis* gene responsible for encoding SPT, the key enzyme for SL *de novo* biosynthesis, which allowed for study of *P. gingivalis* SLs in the context of live bacteria. Employing this mutant, our group had recently shown that SLs limit the host immune response to *P. gingivalis,* as live WT *P. gingivalis* elicited a significantly lower inflammatory response from macrophages compared to the SL-null mutant, which was also found to be recapitulated using only purified OMVs from these strains (13, 25). In our present study, we determined that the uptake of *P. gingivalis* OMVs by macrophages is significantly impacted by SLs, as *P. gingivalis* WT OMVs were taken up significantly less than OMVs from the SL-null mutant. We also found that for *P. gingivalis* WT OMVs, the uptake mechanism by these cells appears to be independent of lipid rafts. While WT OMV uptake was not impacted by lipid raft disruption, we found that *P. gingivalis* SL-null OMVs revealed a strong association with lipid raft-mediated uptake. Innate immune sensing of both WT and SL-null *P. gingivalis* OMVs was dependent on TLR2/MyD88 signaling, but not via TLR4 or TRIF. Lastly, using a global transcriptional approach, we observed profound differences in the transcriptional profiles of macrophages cultured with WT OMVs compared with OMVs from the SL-null mutant and revealed that very early in OMV-host cell interactions, SLs appear to play an important role in limiting the early-phase activation of these immune cells to *P. gingivalis* OMVs.

To the best of our knowledge, our present study is the first detailed investigation to characterize the early host interaction and global transcriptional response to *P. gingivalis* OMVs and is the first study to begin to define the impact microbial SLs have on OMV binding and uptake. Interestingly, employing macrophage modeling and comparing *P. gingivalis* OMV uptake to other bacteria linked to periodontal diseases, including *Treponema denticola* and *Tannerella forsythia*, it was reported that *P. gingivalis* OMVs bound to macrophages more readily than the OMVs of the other organisms. *P. gingivalis* OMVs are more potent activators of NF-κB, yet elicited a less robust inflammatory cytokine response from these cells than the other periodontal disease-associated bacterial OMVs (26, 55), suggesting that there may be some level of control of the host response to *P. gingivalis* OMVs, which is intrinsic to these structures. Our current findings align with these prior reports in that *P. gingivalis* WT OMVs are taken up readily by macrophages (Fig. 2). Importantly, we observed that the increase in *P. gingivalis* OMV uptake depended on the OMV molecular cargo, as the SL-null OMV uptake was significantly more robust than observed with WT OMVs, implicating that *P. gingivalis* SLs play a major role in *P. gingivalis* OMV uptake by macrophages.

The concept that OMV cargo impacts uptake and function of these structures is relatively new, and likely is nuanced between bacteria. For example, and similar to what we observed examining SLs, it was reported that *E. coli* OMV uptake is impacted by its LPS O-antigen cargo, as the LPS O-antigen mutant OMVs were more readily taken up than WT OMVs (56). Although our data support that the SLs are the cargo element responsible for this shift in *P. gingivalis* OMV uptake, we cannot rule out other cargo elements in the OMVs from SL-null *P. gingivalis,* as we previously reported that, in comparison to WT OMVs, the OMVs from SL-null *P. gingivalis* have different arrays of protein cargo (25). Regardless, when taken together, it is clear that shifts in bacterial OMV cargo and the cells these structures interact with have important biological implications in host-pathogen interaction.

Studies have investigated uptake and function of bacterial OMVs using various host cells and have revealed important knowledge as to how bacterial OMVs impact host interaction. Importantly, we reported that *P. gingivalis* SLs are delivered to host cells in an OMV-dependent manner, and that the transfer of these SLs influenced the host cytokine and chemokine response to *P. gingivalis* OMVs (25). Our studies now extend from prior work on the mechanism of *P. gingivalis* OMV uptake (32, 33), and support that there are cell-specific nuances in how *P. gingivalis* OMVs are taken up. In our hands, macrophages do not appear to take up WT *P. gingivalis* OMVs using solely a lipid raft system. Similar studies using SY5Y neuroblastoma and BV-2 microglial cells, reported that WT *P. gingivalis* OMVs were internalized via clathrin-dependent endocytosis (34). Our findings revealed that shifts in OMV SL cargo had a profound influence on OMV uptake. The concept that OMV cargo impacts OMV uptake was established with *Helicobacter pylori* where VacA cytotoxin containing OMVs shift toward clathrin-dependent endocytosis while VacA mutant OMVs favored clathrin-independent uptake (37). Further studies are needed to fully characterize the cell-specific mechanisms of *P. gingivalis* OMV uptake.

It is known that TLRs play an important role in host sensing of *P. gingivalis* and its antigens (27, 57, 58). Indeed, TLR2 and MyD88 are linked to numerous functional endpoints of host-pathogen interaction, including oral bone loss (27, 29, 59–61). Previously, we reported that *P. gingivalis* SLs impact TLR and TLR adaptor molecule gene expression; however, these studies did not address whether these SLs impact TLR sensing of its OMVs (25). Employing TLR receptor and TLR adaptor molecule reporter cell assays, we found there was a significant increase in TLR2 sensing of SL-null OMVs compared to WT OMVs (Fig. 4A). In addition, there were no significant differences observed in OMVs from both strains by TLR4 reporter cells (Fig. 4B). To understand the mechanisms of TLR signaling, we used TLR adaptor molecule reporter cell lines and found that the vast majority of *P. gingivalis* OMV signaling, either WT or SL-null, went through MyD88, but not TRIF (Fig. 4C through E). This aligns with knowledge that TLR2 signaling is MyD88-restricted (62). To the best of our knowledge, these are the first reports directly examining the adaptor molecule involvement in TLR sensing of *P. gingivalis* OMVs. Prior studies show that OMVs from *P. gingivalis* 33277 and strain W50 are sensed by both TLR2 and TLR4 (26, 57, 63). We do not know why our OMVs were not sensed by TLR4; however, this may reflect nuanced differences between *P. gingivalis* strains used (64, 65). Understanding why OMVs from different *P. gingivalis* strains trigger distinct TLR responses is needed, and targeted follow-up studies are needed to elucidate the underlying mechanisms. Our approach to isolate OMVs utilized culture conditions that more resemble the host environment (RPMI-1640 medium supplemented with fetal bovine serum [FBS]) compared to traditional *P. gingivalis* media conditions (26). It is possible that under these conditions, OMV cargo changes could lead to differences in TLR responses. Interestingly, it has also been shown that *P. gingivalis* Lipid A structure can affect TLR4 sensing of *P. gingivalis* OMVs (63). Furthermore, *P. gingivalis* can modify its Lipid A structure in response to environmental change, such as hemin availability and temperature (48). Although the significance of Lipid A structure in our RPMI-derived OMVs in the context of SL status is currently unknown, future studies should be performed to determine the impact of different environmental growth conditions on *P. gingivalis* OMV cargo and downstream host TLR interaction.

Host interaction with bacterial vesicles has primarily focused on defining mechanisms of vesicle uptake, host sensing, cellular toxicity, and, in some cases, functional outcomes such as cytokine and chemokine expression (26, 34, 45, 66). Yet, few studies have investigated the very early global cellular response to bacterial OMV exposure (67). To the best of our knowledge, the present study is the first investigation to define the impact of bacterial lipid OMV cargo on the early-stage global cellular response of the host to OMVs. When we analyzed the early transcription response of THP-1 cells stimulated with WT *P. gingivalis* OMVs, we unexpectedly observed a predominance of downregulated genes compared to unstimulated controls. Pathway analysis reinforced this pattern. These downregulated pathways included key immunomodulation mechanisms such as “cytokine-cytokine receptor interaction,” “chemokine signaling,” and “TNF signaling pathway.” Conversely, THP-1 cells stimulated with SL-null OMVs showed a profile characterized by a stronger percentage of upregulated gene expression than downregulation observed with WT OMVs, highlighting a contribution of SLs found in *P. gingivalis* OMVs to the host response. Investigating this further at the level of pathway enrichment, this trend was further supported as most differentially expressed pathways between SL-null OMV and WT OMV challenged THP-1 cells were upregulated, and interestingly, we observed a correlation between our transcriptional data and cytokine levels for IL-1β and IL-6, as these genes were upregulated (Fig. 6), and protein secretion at higher levels by the SL-null mutant compared to WT (Fig. S1). Little is known about the transcriptional changes between host cells exposed to live *P. gingivalis* and OMVs. However, work by Castillo et al. (68), using live bacteria, shows cell exposure to WT *P. gingivalis* shifted cellular gene expression into a more activated state than what they found with OMVs. In a proteomics approach-based study, it was found that *P. gingivalis* challenged human gingival epithelial cells presented with a mixed array of upregulated and downregulated proteins being expressed, yet similar to our gene transcription findings, more proteins were at lower levels than were found increased; however, this study reported protein content at 6 h and not at 2 h (69). Although we do not understand why WT *P. gingivalis* OMVs drove suppression of gene expression in the early phase of host response, it has been reported by our group that WT *P. gingivalis* W83 bacteria and its OMVs were less inflammatory to THP-1 cells compared with SL-null OMVs (25). This observation of reduced inflammation is broadly aligned with studies of Brown et al. (3), who reported that WT *B. thetaiotaomicron* elicited significantly less inflammation than an SL-null *B. thetaiotaomicron* strain in the context of an inflammatory bowel disease mouse model.

*P. gingivalis* SL-null OMVs elicited enrichment of multiple pathways linked to inflammatory disease, including “Rheumatoid Arthritis” and “Inflammatory Bowel Disease.” The overlap is notable, as periodontitis and rheumatoid arthritis share a similar overexpression of TNF-α, IL-1, and IL-6 (70), and IL-1A overexpression has been reported in rheumatoid arthritis patients (69). Moreover, rheumatoid arthritis targeted biologics, such as IL-6R inhibitors, have been observed to improve periodontitis in rheumatoid arthritis patients (71). This suggests that disrupted cytokine networks may form a mechanistic bridge between these two inflammatory conditions. The enrichment of inflammatory bowel disease pathways in the absence of SLs is equally intriguing, given that several of our top upregulated inflammatory genes (including IL6 and IL18RAP) are implicated in inflammatory bowel disease pathogenesis.

In addition to the array of upregulated THP-1 pathways related to inflammation/host response to infection pathways elicited by SL-null OMVs, we also found an opposite effect on pathways related to carbohydrate metabolism, including “ascorbate and aldarate metabolism,” “fructose and mannose metabolism,” and “glycolysis/gluconeogenesis,” which were suppressed, as were “cholesterol metabolism” in the SL null OMV-treated THP-1 cells compared to WT OMV-treated cells. It is currently unknown why the absence of SLs leads to a suppression of host metabolic pathways. Future studies into how bacterial SLs expression alters host metabolism are needed to functionally understand these connections. Intriguingly, it has been shown that *Bacteroides* SLs can be incorporated into host metabolic pathways (2, 3, 72), and that metabolic shifts can occur in host cells in response to different TLR stimuli to meet the nutritional needs of the responding cell (73, 74). When our findings are taken with those of previous studies (2, 3, 72), an intriguing picture is emerging that bacterial SLs may support and possibly maintain host homeostasis and could be part of an interkingdom communication program between specific microbes and the host.

In conclusion, this study advances our understanding of early host-pathogen interactions by demonstrating that *P. gingivalis* SLs critically shape OMV uptake and the resulting host transcriptional response. These findings provide new mechanistic insight into how OMV cargo influences immune recognition. The exact mechanisms behind this SL-mediated suppressive effect, however, remain unrevealed. Future investigations are essential to delineate how *P. gingivalis*, its sphingolipids, and their OMVs collectively modulate host immune responses.

## MATERIALS AND METHODS

### Bacterial growth

*P. gingivalis* WT strain W83 and its SL-null (∆SPT) mutant (15) were grown anaerobically at 37°C for 3–5 days on Trypticase Soy Broth Blood Agar plates supplemented with 5 µg/mL hemin and 1 µg/mL menadione (TSB-BAPHK), and were then transferred to similarly supplemented Trypticase Soy Broth (TSBHK), and bacteria were cultured for approximately 18 h to the late log phase, where they were subsequently used for generating OMVs.

### OMV purification and isolation

*P. gingivalis* WT and SL-null OMVs were isolated and purified as previously described (25). Briefly, bacteria grown in TSBHK were transferred to RPMI-1640 with 10% heat-inactivated FBS, incubated anaerobically for 24 h, then aerobically for 6 h. OMV-containing supernatants were clarified by centrifugation and 0.2 µm filtration, concentrated by ultrafiltration, and pelleted by ultracentrifugation. Pellets were purified via OptiPrep density-gradient centrifugation, repelleted, resuspended in PBS, and characterized by NTA and electron microscopy before use in experiments.

### Nanoparticle tracking, negative stain-TEM, and fluorescent labeling of *P. gingivalis* OMVs

The concentration and size distribution of the *P. gingivalis* WT and SL-null OMV preparations was determined by NTA using Nanosight NS300 (Malvern Panalytical, Malvern, UK), which is part of the UF Interdisciplinary Center for Biotechnology Research (UF ICBR) collection of core equipment. The morphology of purified OMVs was determined by negative stain-TEM. To accomplish this, a 5 μl volume of OMV suspensions (WT and SL-null) was applied to formvar-coated copper grids, stained with 2% uranyl acetate for 30 s, and analyzed using a 120 kV Tecnai G2 Spirit TWIN transmission electron microscope.

To fluorescently label *P. gingivalis* OMVs, we adapted the method of Jones et al. (39), where we incubated OMVs with Vybrant DiO lipophilic dye (Thermo Fisher Scientific, Waltham, MA) following the manufacturer’s instructions. Briefly, 1 × 10^11^ *P. gingivalis* W83 WT and SL-null OMVs/mL were incubated with Vybrant DiO lipophilic dye at 37°C for 30 min, washed with PBS coupled with ultracentrifugation at 100,000 × *g* at 4°C to remove unincorporated DiO dye, and the labeled OMVs were resuspended in PBS and stored at 4°C for a maximum of 1 month. NTA and negative stain-TEM were used to assess both unlabeled and labeled OMVs, as indicated above. In some experiments, unlabeled and DiO-labeled OMVs were added to THP-1 cells to determine if DiO labeling impacted the elicited THP-1 cytokine/chemokine response (quality control). Culture supernatant fluids were collected and stored frozen at −80°C for multiplex immunoassays. In OMV uptake experiments, DiO-labeled OMVs (1,000 OMVs/cell) were added to THP-1 cells to visualize and quantify uptake via confocal fluorescence microscopy.

### THP-1 cell culture

THP-1 cells, a human monocyte-like cell line (TIB-202, ATCC, Manassas, VA), were cultured in RPMI-1640 media containing 2 mM L-glutamine (Corning, NY), supplemented with 10% heat-inactivated FBS, 10 mM HEPES, 25 mM glucose, 1 mM sodium pyruvate, 18 mM sodium bicarbonate, 55 μM β-mercaptoethanol, and 100 μg/mL antibiotics (penicillin and streptomycin; P/S) at 37°C in a 5% CO_2_ incubator, and were differentiated into a macrophage-like state in complete RPMI-1640 media containing 100 ng/mL phorbol-12-myristate-13-acetate (PMA; Sigma-Aldrich, St. Louis, MO), and 1 mL was added to wells of 24-well plates and incubated for 48 h (25). Following differentiation, THP-1 cells were washed, fresh complete media was added to each well, and these cells were used in experiments outlined below.

### *P. gingivalis* OMV uptake assays

For OMV uptake experiments, THP-1 cells were cultured on sterile standard glass coverslips, either untreated or challenged with WT and SL-null DiO-labeled *P. gingivalis* OMVs (1,000 OMVs/cell) for 2 h. Glass coverslips were retrieved and incubated at room temperature for 1 min with 0.025% Trypan Blue solution (Corning, NY) in PBS to quench the fluorescence of non-internalized DiO-labeled OMVs (39). Next, coverslips were incubated with 18 µM CellTracker Red CMTPX dye (Thermo Fisher Scientific) in RPMI-1640 for 15 min at 37°C to label cell membranes. Cells were then fixed on the coverslips with 4% paraformaldehyde, and after washing, coverslips were mounted to microscope slides using DAPI-Fluoromount G mounting medium (Southern Biotech). OMV uptake was measured using confocal fluorescence microscopy; we first established the background fluorescence from THP-1 cells treated with medium alone. 3-D Z-stack images from representative fields were taken with 60× objective using a Nikon Ti2 confocal fluorescence microscope (Melville, NY), then converted to 2-D high dynamic range images for analysis with NIS Elements software (Melville, NY). For fluorescence quantification of OMV uptake (DiO/green channel), images from a minimum of 10 representative fields per sample were digitally captured, and then we traced the perimeter of cells to define the representative ROIs. This provided a direct measure of fluorescence associated with each cell. Lastly, we applied a binary mask to include only the DiO/OMV fluorescence channel in our analysis. The sum of the OMV fluorescence area (µm^2^) of each ROI was measured, and the total fluorescence area across all ROIs was calculated for 250 total THP-1 cells.

For OMV uptake inhibition studies, differentiated THP-1 cells were untreated or treated with 10 mM MβCD (inhibitor of lipid rafts) in RPMI-1640 media without supplements or cultured in medium alone for 30 min (32). MβCD treatment was then removed; THP-1 cells were then challenged with DiO-labeled WT or SL-null OMVs (1,000 OMVs/cell) in complete RPMI media without antibiotics as described above; microscope slides were also prepared as above.

### Host cell cytokine/chemokine profiling

THP-1 cell culture supernatant fluids were thawed, and levels of TNF-α, IL-1β, IL-6, IL-10, IL-8, and RANTES were determined using 6-plex Human Luminex Discovery Assays (Biotechne, Minneapolis, MN). Data were acquired using MAGPIX Luminex system running xPONENT 3.1 software (Luminex, Austin, TX). Data were analyzed using a five-parameter logistic spline curve-fitting method via Milliplex Analyst V5.1 software (Vigene Tech, Carlisle, MA). Nonlabeled WT vs DiO-labeled WT and nonlabeled SL-null vs DiO-labeled SL-null OMV challenge data represent five biological replicates.

### TLR- and TLR-signaling NF-κB reporter cells

HEK-Blue hTLR2 and hTLR4 cells were used to assess TLR sensing of *P. gingivalis* OMVs, while THP-1 Dual parental cell line (CTRL), TRIF-KO, and MyD88-KO NF-κB reporter cells were used to examine downstream TLR signaling. All cell lines were purchased from Invivogen (San Diego, CA) and handled following the manufacturer’s protocols (Invivogen). Frozen stocks of each cell line were stored in liquid nitrogen, and cultures were discarded before reaching 20 passages.

### Reporter cell assays

HEK-Blue TLR2 and TLR4 reporter cells and all THP-1 Dual cells: parental THP-1 Dual or control (CTRL), TRIF-KO, and MyD88-KO cells were challenged with unlabeled WT and SL-null OMVs at 1,000 OMVs/cell (25) for 22 h (26). Positive controls for TLR2 and TLR4 sensing included 10 ng/mL Pam2CSK4 (TLR2 agonist) and 100 µg/mL Ultrapure *E. coli* LPS (TLR4 agonist) (Invivogen), while positive controls for MyD88-dependent and MyD88-independent sensing included 10 ng/mL Pam2CSK4 (MyD88-dependent agonist) and 1 ng/mL recombinant TNF-α (MyD88-independent agonist) (Invivogen), respectively. Baseline response levels for each cell line were established using the unchallenged controls cultured in detection medium with an equal volume of vehicle (endotoxin-free water). Following WT and SL-null OMV challenge, SEAP levels in reporter cell supernatants were measured via absorbance at 620 nm using H1 Synergy plate reader (Agilent Technologies, Santa Clara, CA). Background absorbance was subtracted from the supernatant fluids of cells treated with detection medium alone. Recombinant SEAP protein (0.1 ng/mL–100 ng/mL) (Invivogen, San Diego, CA) in detection medium was used to generate a standard curve for SEAP quantification.

### THP-1 mRNA isolation and transcriptomic analysis

For transcriptomics assays, PMA-differentiated THP-1 cells were cultured as above for 2 h in antibiotic-free RPMI-1640 medium, or medium containing either *P. gingivalis* WT or SL-null OMVs (1,000 OMVs/cell). Culture supernatant fluids were removed, the THP-1 cells were lysed using Qiagen RLT lysis buffer (Germantown, MD), and cell lysates were stored frozen at −80°C until use. mRNA was isolated and purified from the cell lysates using RNeasy Plus Mini Kits (Qiagen; Germantown, MD). Quality assessments of all mRNA samples, library preparation, and paired-end sequencing (length = 101) were performed at UF ICBR Gene Expression and Genotyping Core. The bioinformatics pipeline was run entirely in the U. Florida HiperGator cluster computer. Quality control of raw sequences was conducted with FASTQC (75). Adapter sequences were trimmed with Cutadapt (76), sequences with length less than 75 nucleotides were removed, and 5′ and 3′ end bases with Phred score less than 30 were trimmed. Raw trimmed sequences were then mapped to the *Homo sapiens* GRCh38 primary reference genome (BioProject PRJNA31257). Local mapping of the curated reads was done with BWA-mem (77). Handling, sorting, and conversion of the alignment files was executed with the Samtools suite (78). Finally, the counts of transcripts along the reference genomes were conducted with HTSeq-count (79). Count files from all samples were parsed together in a matrix as input for the statistical and cluster analysis in R (80). Statistical analysis was conducted with the “edgeR” (81). Plotting was done with the “plot” base functions and the package “ggplot2” (82). Pathway and Gene Ontology analyses were conducted using the packages clusterProfiler (83) and pathview (84). Heatmap was generated with the package pheatmap (85).

### Statistical analysis

Experiments consisted of two to five biological replicates. Statistical analysis of data sets included the use of unpaired *t*-test using GraphPad Prism 10 software (GraphPad, San Diego, CA), and unless otherwise indicated, data are presented as means ± SEM. A *P* < 0.05 was considered significant.

## Data Availability

Gene expression data have been deposited in the NCBI Gene Expression Omnibus (GEO) database (https://www.ncbi.nlm.nih.gov/geo/) under GEO Series accession number GSE313285.

## SUPPLEMENTAL MATERIAL

Supplemental material iai.00736-25-s0001.docx Fig. S1 to S6; Tables S1 to S9.

ASM does not own the copyrights to Supplemental Material that may be linked to, or accessed through, an article. The authors have granted ASM a non-exclusive, world-wide license to publish the Supplemental Material files. Please contact the corresponding author directly for reuse.
