# Supplementary material for: Porphyromonas gingivalis sphingolipids affect early responses of THP-1 macrophages to outer membrane vesicles
Source: Infect Immun. 2026 Jul 6;94(8):e00736-25. doi: 10.1128/iai.00736-25 (PMC13460102; doi:10.1128/iai.00736-25)
Supplement: Supplementary file 1 [file iai.00736-25-s0001.docx]

**Supplemental material:**

**Figure S1.** **DiO labeling does not substantially alter THP-1 macrophage cytokine expression elicited by *P. gingivalis* OMVs.** THP-1 cells were challenged for 2 hours with unlabeled (DiO-) or labelled (DiO+) *P. gingivalis* SL-containing (WT) and SL-null (ΔSPT) OMVs, were washed, and after 2hr ,cell culture supernatant fluid samples were collected and levels of cytokines and chemokines were determined by Luminex immunoassay. Media alone (Med) served as the untreated control. Bars represent mean ± SEM values, n = 4-5 independent experiments. Data were analyzed by unpaired T test and ns = not significant, * = *p*<0.05.

**Figure S2. Cholesterol depletion from THP-1 macrophages cultured with MβCD.** THP-1 macrophages were untreated (Med; open bar) or treated with increasing concentrations (mM) of MβCD for 30 mins. Following washing, cholesterol concentration was measured. Bars represent mean ± SEM values, n = 2-3 independent experiments. unpaired T test was used to compare each treatment to untreated (Med); ns = not significant, ** = *p*<0.01, *** = *p*<0.001.

**Figure S3. Impact of MβCD concentration on THP-1 macrophage viability.** THP-1 macrophages were untreated (Med; open bar) or treated for 30 mins with indicated MβCD concentrations, and cell viability was determined by MTT assay. Bars represent mean ± SEM values, n = 4 independent experiments. Data were analyzed by unpaired T test and ns = not significant (all comparisons; *p*≥ 0.05)

**Figure. S4. Impact of lipid raft disruption on *P. gingivalis* OMV-elicited inflammation.** 5x10^5^ PMA-treated THP-1 cells were either treated with medium alone (Med) or with 10 mM MβCD for 30 min prior to WT *P. gingivalis* OMVs (WT) or SL-null OMVs (DSPT) OMV challenged with labelled *P. gingivalis* W83 (WT or ΔSPT) OMVs at 1000 OMVs/cell. Cell culture supernatant samples were collected and cytokine and chemokine levels of each indicated mediator was measured by Luminex immunoassay. Cells treated with medium alone (Med) served as the baseline for cytokine and chemokine expression. Cells treated with MβCD alone was used to test MβCD impact on THP-1 elicited inflammation independent of OMVs. Bars represent mean ± SEM values, n = 4 independent experiments. Data were analyzed by unpaired T test and ns = not significant (all comparisons; p≥ 0.05)

**Figure S5. Heatmap of mean z-scored gene expression values for all genes with p<0.05 across groups.** Rows represent genes; columns are group means (n=3). Colors represent relative expression (purple to blue = low, yellow = high). Hierarchical clustering highlights group-specific expression patterns. Conditions (cond) include: CTL=untreated control (green), WT=WT *P. gingivalis* OMV-treated group (pink), SPT=SL-null *P. gingivalis* OMV-treated group (purple).

**Figure S6. Volcano plot illustrating the transcriptome of THP-1 macrophages co-cultured with SL-null OMVs (SPT) versus medium only (CTR)**. Differential expression analyses were performed with cutoffs for log_2_-fold change >1.0 and p-value < 0.05. Upregulated genes are shown in red and downregulated genes in blue.

**Table S1.** **List of all differentially expressed genes (DEGs) of THP-1 cells co-cultured with WT OMVs compared to untreated THP-1 cells.**

| geneID | Name | Description | logFC | pValue | Change |
| --- | --- | --- | --- | --- | --- |
| ENSG00000115008 | IL1A | interleukin_1_alpha_[Source:HGNC_Symbol%3BAcc:HGNC:5991] | -5.6960131 | 0.00285565 | Down |
| ENSG00000205436 | EXOC3L4 | exocyst_complex_component_3_like_4_[Source:HGNC_Symbol%3BAcc:HGNC:20120] | -5.2284584 | 0.00908078 | Down |
| ENSG00000130997 | POLN | DNA_polymerase_nu_[Source:HGNC_Symbol%3BAcc:HGNC:18870] | -5.1598777 | 0.02442348 | Down |
| ENSG00000205929 | C21orf62 | chromosome_21_open_reading_frame_62_[Source:HGNC_Symbol%3BAcc:HGNC:1305] | -5.1229108 | 0.02267514 | Down |
| ENSG00000108700 | CCL8 | C-C_motif_chemokine_ligand_8_[Source:HGNC_Symbol%3BAcc:HGNC:10635] | -5.0930849 | 0.01190962 | Down |
| ENSG00000213221 | DNLZ | DNL-type_zinc_finger_[Source:HGNC_Symbol%3BAcc:HGNC:33879] | -5.0705312 | 0.02561203 | Down |
| ENSG00000176204 | LRRTM4 | leucine_rich_repeat_transmembrane_neuronal_4_[Source:HGNC_Symbol%3BAcc:HGNC:19411] | -4.8937372 | 0.03487731 | Down |
| ENSG00000276231 | PIK3R6 | phosphoinositide-3-kinase_regulatory_subunit_6_[Source:HGNC_Symbol%3BAcc:HGNC:27101] | -4.8902401 | 0.01331276 | Down |
| ENSG00000197191 | CYSRT1 | cysteine_rich_tail_1_[Source:HGNC_Symbol%3BAcc:HGNC:30529] | -4.7894923 | 0.03694485 | Down |
| ENSG00000175564 | UCP3 | uncoupling_protein_3_[Source:HGNC_Symbol%3BAcc:HGNC:12519] | -4.7553608 | 0.02276413 | Down |
| ENSG00000101115 | SALL4 | spalt_like_transcription_factor_4_[Source:HGNC_Symbol%3BAcc:HGNC:15924] | -4.7440209 | 0.0206271 | Down |
| ENSG00000260371 |  | novel_protein | -4.7411369 | 0.02053944 | Down |
| ENSG00000151715 | TMEM45B | transmembrane_protein_45B_[Source:HGNC_Symbol%3BAcc:HGNC:25194] | -4.7192916 | 0.04652909 | Down |
| ENSG00000111728 | ST8SIA1 | ST8_alpha-N-acetyl-neuraminide_alpha-2%2C8-sialyltransferase_1_[Source:HGNC_Symbol%3BAcc:HGNC:10869] | -4.6446935 | 0.04900786 | Down |
| ENSG00000146374 | RSPO3 | R-spondin_3_[Source:HGNC_Symbol%3BAcc:HGNC:20866] | -4.6033988 | 0.02191278 | Down |
| ENSG00000288675 |  | proapoptotic_nucleolar_protein_1 | -4.448116 | 0.03628698 | Down |
| ENSG00000184619 | KRBA2 | KRAB-A_domain_containing_2_[Source:HGNC_Symbol%3BAcc:HGNC:26989] | -4.4236392 | 0.03900818 | Down |
| ENSG00000118514 | ALDH8A1 | aldehyde_dehydrogenase_8_family_member_A1_[Source:HGNC_Symbol%3BAcc:HGNC:15471] | -3.5317029 | 0.01111518 | Down |
| ENSG00000136944 | LMX1B | LIM_homeobox_transcription_factor_1_beta_[Source:HGNC_Symbol%3BAcc:HGNC:6654] | -3.3307998 | 0.01563607 | Down |
| ENSG00000163739 | CXCL1 | C-X-C_motif_chemokine_ligand_1_[Source:HGNC_Symbol%3BAcc:HGNC:4602] | -3.2750438 | 0.00024284 | Down |
| ENSG00000276070 | CCL4L2 | C-C_motif_chemokine_ligand_4_like_2_[Source:HGNC_Symbol%3BAcc:HGNC:24066] | -3.2492362 | 0.01021927 | Down |
| ENSG00000275302 | CCL4 | C-C_motif_chemokine_ligand_4_[Source:HGNC_Symbol%3BAcc:HGNC:10630] | -3.1486049 | 0.00458155 | Down |
| ENSG00000177692 | DNAJC28 | DnaJ_heat_shock_protein_family_(Hsp40)_member_C28_[Source:HGNC_Symbol%3BAcc:HGNC:1297] | -3.1200116 | 0.0302203 | Down |
| ENSG00000254995 | STX16-NPEPL1 | STX16-NPEPL1_readthrough_(NMD_candidate)_[Source:HGNC_Symbol%3BAcc:HGNC:41993] | -3.1111504 | 0.03933992 | Down |

| ENSG00000187122 | SLIT1 | slit_guidance_ligand_1_[Source:HGNC_Symbol%3BAcc:HGNC:11085] | -2.9086386 | 0.02427409 | Down |
| --- | --- | --- | --- | --- | --- |
| ENSG00000137393 | RNF144B | ring_finger_protein_144B_[Source:HGNC_Symbol%3BAcc:HGNC:21578] | -2.8493114 | 0.04427845 | Down |
| ENSG00000179388 | EGR3 | early_growth_response_3_[Source:HGNC_Symbol%3BAcc:HGNC:3240] | -2.8349106 | 0.02287481 | Down |
| ENSG00000144802 | NFKBIZ | NFKB_inhibitor_zeta_[Source:HGNC_Symbol%3BAcc:HGNC:29805] | -2.711525 | 0.00017812 | Down |
| ENSG00000100429 | HDAC10 | histone_deacetylase_10_[Source:HGNC_Symbol%3BAcc:HGNC:18128] | -2.6908116 | 0.02480543 | Down |
| ENSG00000186094 | AGBL4 | ATP/GTP_binding_protein_like_4_[Source:HGNC_Symbol%3BAcc:HGNC:25892] | -2.6501166 | 0.01840071 | Down |
| ENSG00000165521 | EML5 | EMAP_like_5_[Source:HGNC_Symbol%3BAcc:HGNC:18197] | -2.6336503 | 0.01183083 | Down |
| ENSG00000169429 | CXCL8 | C-X-C_motif_chemokine_ligand_8_[Source:HGNC_Symbol%3BAcc:HGNC:6025] | -2.4524762 | 0.00032367 | Down |
| ENSG00000118503 | TNFAIP3 | TNF_alpha_induced_protein_3_[Source:HGNC_Symbol%3BAcc:HGNC:11896] | -2.4501541 | 0.00026329 | Down |
| ENSG00000023445 | BIRC3 | baculoviral_IAP_repeat_containing_3_[Source:HGNC_Symbol%3BAcc:HGNC:591] | -2.2851578 | 0.04436658 | Down |
| ENSG00000120738 | EGR1 | early_growth_response_1_[Source:HGNC_Symbol%3BAcc:HGNC:3238] | -2.2470115 | 0.03113377 | Down |
| ENSG00000242950 | ERVW-1 | endogenous_retrovirus_group_W_member_1%2C_envelope_[Source:HGNC_Symbol%3BAcc:HGNC:13525] | -2.2343894 | 0.02206807 | Down |
| ENSG00000089123 | TASP1 | taspase_1_[Source:HGNC_Symbol%3BAcc:HGNC:15859] | -2.210986 | 0.0140766 | Down |
| ENSG00000154040 | CABYR | calcium_binding_tyrosine_phosphorylation_regulated_[Source:HGNC_Symbol%3BAcc:HGNC:15569] | -2.1497447 | 0.02585136 | Down |
| ENSG00000277632 | CCL3 | C-C_motif_chemokine_ligand_3_[Source:HGNC_Symbol%3BAcc:HGNC:10627] | -2.0072311 | 0.01610298 | Down |
| ENSG00000188158 | NHS | NHS_actin_remodeling_regulator_[Source:HGNC_Symbol%3BAcc:HGNC:7820] | -2.001887 | 0.0306762 | Down |
| ENSG00000276085 | CCL3L1 | C-C_motif_chemokine_ligand_3_like_1_[Source:HGNC_Symbol%3BAcc:HGNC:10628] | -1.9933699 | 0.03497988 | Down |
| ENSG00000118849 | RARRES1 | retinoic_acid_receptor_responder_1_[Source:HGNC_Symbol%3BAcc:HGNC:9867] | -1.950616 | 0.0486857 | Down |
| ENSG00000166762 | CATSPER2 | cation_channel_sperm_associated_2_[Source:HGNC_Symbol%3BAcc:HGNC:18810] | -1.7046569 | 0.02248101 | Down |
| ENSG00000125538 | IL1B | interleukin_1_beta_[Source:HGNC_Symbol%3BAcc:HGNC:5992] | -1.6666752 | 0.02539659 | Down |
| ENSG00000156269 | NAA11 | N-alpha-acetyltransferase_11%2C_NatA_catalytic_subunit_[Source:HGNC_Symbol%3BAcc:HGNC:28125] | -1.6583477 | 0.0387782 | Down |
| ENSG00000186654 | PRR5 | proline_rich_5_[Source:HGNC_Symbol%3BAcc:HGNC:31682] | -1.6092147 | 0.02787427 | Down |
| ENSG00000120210 | INSL6 | insulin_like_6_[Source:HGNC_Symbol%3BAcc:HGNC:6089] | -1.5942197 | 0.0135678 | Down |
| ENSG00000150630 | VEGFC | vascular_endothelial_growth_factor_C_[Source:HGNC_Symbol%3BAcc:HGNC:12682] | -1.5590347 | 0.02548932 | Down |
| ENSG00000272772 |  | novel_protein | -1.546581 | 0.040635 | Down |

**Table S1. Continued.**

| ENSG00000180509 | KCNE1 | potassium_voltage-gated_channel_subfamily_E_regulatory_subunit_1_[Source:HGNC_Symbol%3BAcc:HGNC:6240] | -1.5025821 | 0.04445327 | Down |
| --- | --- | --- | --- | --- | --- |
| ENSG00000105717 | PBX4 | PBX_homeobox_4_[Source:HGNC_Symbol%3BAcc:HGNC:13403] | -1.3271206 | 0.04030655 | Down |
| ENSG00000081059 | TCF7 | transcription_factor_7_[Source:HGNC_Symbol%3BAcc:HGNC:11639] | -1.2679836 | 0.04257192 | Down |
| ENSG00000112096 | SOD2 | superoxide_dismutase_2_[Source:HGNC_Symbol%3BAcc:HGNC:11180] | -1.1934777 | 0.01492422 | Down |
| ENSG00000163874 | ZC3H12A | zinc_finger_CCCH-type_containing_12A_[Source:HGNC_Symbol%3BAcc:HGNC:26259] | -1.1714337 | 0.01193964 | Down |
| ENSG00000115919 | KYNU | kynureninase_[Source:HGNC_Symbol%3BAcc:HGNC:6469] | -1.1561008 | 0.03453283 | Down |
| ENSG00000123700 | KCNJ2 | potassium_inwardly_rectifying_channel_subfamily_J_member_2_[Source:HGNC_Symbol%3BAcc:HGNC:6263] | -1.0988841 | 0.03574457 | Down |
| ENSG00000159388 | BTG2 | BTG_anti-proliferation_factor_2_[Source:HGNC_Symbol%3BAcc:HGNC:1131] | -1.0678483 | 0.01569015 | Down |
| ENSG00000100906 | NFKBIA | NFKB_inhibitor_alpha_[Source:HGNC_Symbol%3BAcc:HGNC:7797] | -1.0661997 | 0.0285348 | Down |
| ENSG00000137965 | IFI44 | interferon_induced_protein_44_[Source:HGNC_Symbol%3BAcc:HGNC:16938] | 1.22108488 | 0.04584356 | Up |
| ENSG00000141027 | NCOR1 | nuclear_receptor_corepressor_1_[Source:HGNC_Symbol%3BAcc:HGNC:7672] | 1.24867838 | 0.02341214 | Up |
| ENSG00000134321 | RSAD2 | radical_S-adenosyl_methionine_domain_containing_2_[Source:HGNC_Symbol%3BAcc:HGNC:30908] | 1.28757299 | 0.03923286 | Up |
| ENSG00000171246 | NPTX1 | neuronal_pentraxin_1_[Source:HGNC_Symbol%3BAcc:HGNC:7952] | 1.29703769 | 0.0299007 | Up |
| ENSG00000171320 | ESCO2 | establishment_of_sister_chromatid_cohesion_N-acetyltransferase_2_[Source:HGNC_Symbol%3BAcc:HGNC:27230] | 1.33593278 | 0.04272171 | Up |
| ENSG00000185818 | NAT8L | N-acetyltransferase_8_like_[Source:HGNC_Symbol%3BAcc:HGNC:26742] | 1.35486735 | 0.04378902 | Up |
| ENSG00000116711 | PLA2G4A | phospholipase_A2_group_IVA_[Source:HGNC_Symbol%3BAcc:HGNC:9035] | 1.38824548 | 0.02445184 | Up |
| ENSG00000152147 | GEMIN6 | gem_nuclear_organelle_associated_protein_6_[Source:HGNC_Symbol%3BAcc:HGNC:20044] | 1.40115558 | 0.02616321 | Up |
| ENSG00000189057 | FAM111B | FAM111_trypsin_like_peptidase_B_[Source:HGNC_Symbol%3BAcc:HGNC:24200] | 1.47625092 | 0.03594474 | Up |
| ENSG00000137959 | IFI44L | interferon_induced_protein_44_like_[Source:HGNC_Symbol%3BAcc:HGNC:17817] | 1.50170212 | 0.04301556 | Up |
| ENSG00000172878 | METAP1D | methionyl_aminopeptidase_type_1D%2C_mitochondrial_[Source:HGNC_Symbol%3BAcc:HGNC:32583] | 1.5534022 | 0.02521812 | Up |
| ENSG00000006625 | GGCT | gamma-glutamylcyclotransferase_[Source:HGNC_Symbol%3BAcc:HGNC:21705] | 1.56275286 | 0.03001271 | Up |
| ENSG00000138061 | CYP1B1 | cytochrome_P450_family_1_subfamily_B_member_1_[Source:HGNC_Symbol%3BAcc:HGNC:2597] | 1.72807377 | 0.03205843 | Up |
| ENSG00000163508 | EOMES | eomesodermin_[Source:HGNC_Symbol%3BAcc:HGNC:3372] | 2.01017016 | 0.03515215 | Up |
| ENSG00000156172 | C8orf37 | chromosome_8_open_reading_frame_37_[Source:HGNC_Symbol%3BAcc:HGNC:27232] | 2.04326129 | 0.03064234 | Up |
| ENSG00000102539 | MLNR | motilin_receptor_[Source:HGNC_Symbol%3BAcc:HGNC:4495] | 2.25998726 | 0.00301228 | Up |

**Table S1. Continued.**

**Table S1. Continued.**

| ENSG00000164920 | OSR2 | odd-skipped_related_transciption_factor_2_[Source:HGNC_Symbol%3BAcc:HGNC:15830] | 2.55367492 | 0.02114596 | Up |
| --- | --- | --- | --- | --- | --- |
| ENSG00000117877 | POLR1G | RNA_polymerase_I_subunit_G_[Source:HGNC_Symbol%3BAcc:HGNC:24219] | 2.59657675 | 0.01209038 | Up |
| ENSG00000146966 | DENND2A | DENN_domain_containing_2A_[Source:HGNC_Symbol%3BAcc:HGNC:22212] | 2.66099497 | 0.03994366 | Up |
| ENSG00000169744 | LDB2 | LIM_domain_binding_2_[Source:HGNC_Symbol%3BAcc:HGNC:6533] | 2.70555331 | 0.04510801 | Up |
| ENSG00000165685 | TMEM52B | transmembrane_protein_52B_[Source:HGNC_Symbol%3BAcc:HGNC:26438] | 2.80257247 | 0.02325949 | Up |
| ENSG00000155307 | SAMSN1 | SAM_domain%2C_SH3_domain_and_nuclear_localization_signals_1_[Source:HGNC_Symbol%3BAcc:HGNC:10528] | 2.80557379 | 0.00524709 | Up |
| ENSG00000088538 | DOCK3 | dedicator_of_cytokinesis_3_[Source:HGNC_Symbol%3BAcc:HGNC:2989] | 2.8852017 | 0.00666571 | Up |
| ENSG00000196335 | STK31 | serine/threonine_kinase_31_[Source:HGNC_Symbol%3BAcc:HGNC:11407] | 3.22855523 | 0.04390134 | Up |
| ENSG00000131016 | AKAP12 | A-kinase_anchoring_protein_12_[Source:HGNC_Symbol%3BAcc:HGNC:370] | 3.48877021 | 0.02364218 | Up |
| ENSG00000168152 | THAP9 | THAP_domain_containing_9_[Source:HGNC_Symbol%3BAcc:HGNC:23192] | 3.50640227 | 0.03990951 | Up |
| ENSG00000187957 | DNER | delta/notch_like_EGF_repeat_containing_[Source:HGNC_Symbol%3BAcc:HGNC:24456] | 3.77746985 | 0.03045034 | Up |
| ENSG00000087510 | TFAP2C | transcription_factor_AP-2_gamma_[Source:HGNC_Symbol%3BAcc:HGNC:11744] | 3.80138887 | 0.03083051 | Up |
| ENSG00000117228 | GBP1 | guanylate_binding_protein_1_[Source:HGNC_Symbol%3BAcc:HGNC:4182] | 4.2038381 | 0.00366032 | Up |
| ENSG00000288721 |  | novel_protein | 4.61713792 | 0.04582036 | Up |
| ENSG00000159409 | CELF3 | CUGBP_Elav-like_family_member_3_[Source:HGNC_Symbol%3BAcc:HGNC:11967] | 4.75800845 | 0.0411681 | Up |
| ENSG00000279164 |  | TEC | 4.79715069 | 0.01868611 | Up |
| ENSG00000105146 | AURKC | aurora_kinase_C_[Source:HGNC_Symbol%3BAcc:HGNC:11391] | 4.83434507 | 0.01430101 | Up |
| ENSG00000214050 | FBXO16 | F-box_protein_16_[Source:HGNC_Symbol%3BAcc:HGNC:13618] | 4.91725997 | 0.01595939 | Up |
| ENSG00000145864 | GABRB2 | gamma-aminobutyric_acid_type_A_receptor_subunit_beta2_[Source:HGNC_Symbol%3BAcc:HGNC:4082] | 5.05531994 | 0.02581033 | Up |
| ENSG00000174469 | CNTNAP2 | contactin_associated_protein_2_[Source:HGNC_Symbol%3BAcc:HGNC:13830] | 5.28963392 | 0.00706695 | Up |
| ENSG00000165959 | CLMN | calmin_[Source:HGNC_Symbol%3BAcc:HGNC:19972] | 5.50373061 | 0.00159298 | Up |

**Table S2. List of all DEGs of THP-1 cells co-cultured with ∆SPT OMVs compared to co-cultured with WT OMVs.**

| geneID | Name | Description | logFC | pValue | Change |
| --- | --- | --- | --- | --- | --- |
| ENSG00000256100 |  | novel_transcript | -5.17196 | 0.01567 | Down |
| ENSG00000267127 |  | novel_protein | -5.10192 | 0.027433 | Down |
| ENSG00000159409 | CELF3 | CUGBP_Elav-like_family_member_3_[Source:HGNC_Symbol%3BAcc:HGNC:11967] | -4.74664 | 0.045952 | Down |
| ENSG00000126246 | IGFLR1 | IGF_like_family_receptor_1_[Source:HGNC_Symbol%3BAcc:HGNC:23620] | -4.73527 | 0.041046 | Down |
| ENSG00000181690 | PLAG1 | PLAG1_zinc_finger_[Source:HGNC_Symbol%3BAcc:HGNC:9045] | -4.69874 | 0.02718 | Down |
| ENSG00000105289 | TJP3 | tight_junction_protein_3_[Source:HGNC_Symbol%3BAcc:HGNC:11829] | -4.51123 | 0.042821 | Down |
| ENSG00000137968 | SLC44A5 | solute_carrier_family_44_member_5_[Source:HGNC_Symbol%3BAcc:HGNC:28524] | -4.10777 | 0.010062 | Down |
| ENSG00000179240 | GVQW3 | GVQW_motif_containing_3_[Source:HGNC_Symbol%3BAcc:HGNC:51239] | -3.54308 | 0.033631 | Down |
| ENSG00000135312 | HTR1B | 5-hydroxytryptamine_receptor_1B_[Source:HGNC_Symbol%3BAcc:HGNC:5287] | -3.26466 | 0.003905 | Down |
| ENSG00000124143 | ARHGAP40 | Rho_GTPase_activating_protein_40_[Source:HGNC_Symbol%3BAcc:HGNC:16226] | -2.92722 | 0.016331 | Down |
| ENSG00000155307 | SAMSN1 | SAM_domain%2C_SH3_domain_and_nuclear_localization_signals_1_[Source:HGNC_Symbol%3BAcc:HGNC:10528] | -2.7001 | 0.008983 | Down |
| ENSG00000139044 | B4GALNT3 | beta-1%2C4-N-acetyl-galactosaminyltransferase_3_[Source:HGNC_Symbol%3BAcc:HGNC:24137] | -2.39011 | 0.019807 | Down |
| ENSG00000162526 | TSSK3 | testis_specific_serine_kinase_3_[Source:HGNC_Symbol%3BAcc:HGNC:15473] | -2.36314 | 0.04686 | Down |
| ENSG00000140030 | GPR65 | G_protein-coupled_receptor_65_[Source:HGNC_Symbol%3BAcc:HGNC:4517] | -2.29139 | 0.037787 | Down |
| ENSG00000123405 | NFE2 | nuclear_factor%2C_erythroid_2_[Source:HGNC_Symbol%3BAcc:HGNC:7780] | -2.27401 | 0.019868 | Down |
| ENSG00000138061 | CYP1B1 | cytochrome_P450_family_1_subfamily_B_member_1_[Source:HGNC_Symbol%3BAcc:HGNC:2597] | -2.21242 | 0.010014 | Down |
| ENSG00000146700 | SSC4D | scavenger_receptor_cysteine_rich_family_member_with_4_domains_[Source:HGNC_Symbol%3BAcc:HGNC:14461] | -1.94135 | 0.046512 | Down |
| ENSG00000102539 | MLNR | motilin_receptor_[Source:HGNC_Symbol%3BAcc:HGNC:4495] | -1.87405 | 0.015104 | Down |
| ENSG00000171246 | NPTX1 | neuronal_pentraxin_1_[Source:HGNC_Symbol%3BAcc:HGNC:7952] | -1.756 | 0.009563 | Down |
| ENSG00000178175 | ZNF366 | zinc_finger_protein_366_[Source:HGNC_Symbol%3BAcc:HGNC:18316] | -1.5908 | 0.025554 | Down |
| ENSG00000185614 | INKA1 | inka_box_actin_regulator_1_[Source:HGNC_Symbol%3BAcc:HGNC:32480] | -1.55841 | 0.007887 | Down |
| ENSG00000168061 | SAC3D1 | SAC3_domain_containing_1_[Source:HGNC_Symbol%3BAcc:HGNC:30179] | -1.55532 | 0.032298 | Down |
| ENSG00000145723 | GIN1 | gypsy_retrotransposon_integrase_1_[Source:HGNC_Symbol%3BAcc:HGNC:25959] | -1.55531 | 0.045377 | Down |
| ENSG00000198597 | ZNF536 | zinc_finger_protein_536_[Source:HGNC_Symbol%3BAcc:HGNC:29025] | -1.51239 | 0.037884 | Down |
| ENSG00000133561 | GIMAP6 | GTPase%2C_IMAP_family_member_6_[Source:HGNC_Symbol%3BAcc:HGNC:21918] | -1.46543 | 0.033333 | Down |

| ENSG00000168672 | LRATD2 | LRAT_domain_containing_2_[Source:HGNC_Symbol%3BAcc:HGNC:24166] | -1.44891 | 0.03795 | Down |
| --- | --- | --- | --- | --- | --- |
| ENSG00000110665 | C11orf21 | chromosome_11_open_reading_frame_21_[Source:HGNC_Symbol%3BAcc:HGNC:13231] | -1.44474 | 0.016181 | Down |
| ENSG00000113327 | GABRG2 | gamma-aminobutyric_acid_type_A_receptor_subunit_gamma2_[Source:HGNC_Symbol%3BAcc:HGNC:4087] | -1.42777 | 0.046407 | Down |
| ENSG00000177191 | B3GNT8 | UDP-GlcNAc:betaGal_beta-1%2C3-N-acetylglucosaminyltransferase_8_[Source:HGNC_Symbol%3BAcc:HGNC:24139] | -1.38138 | 0.025225 | Down |
| ENSG00000125352 | RNF113A | ring_finger_protein_113A_[Source:HGNC_Symbol%3BAcc:HGNC:12974] | -1.3591 | 0.03274 | Down |
| ENSG00000125735 | TNFSF14 | TNF_superfamily_member_14_[Source:HGNC_Symbol%3BAcc:HGNC:11930] | -1.34919 | 0.011941 | Down |
| ENSG00000176749 | CDK5R1 | cyclin_dependent_kinase_5_regulatory_subunit_1_[Source:HGNC_Symbol%3BAcc:HGNC:1775] | -1.32533 | 0.040281 | Down |
| ENSG00000178726 | THBD | thrombomodulin_[Source:HGNC_Symbol%3BAcc:HGNC:11784] | -1.28973 | 0.035169 | Down |
| ENSG00000133246 | PRAM1 | PML-RARA_regulated_adaptor_molecule_1_[Source:HGNC_Symbol%3BAcc:HGNC:30091] | -1.17952 | 0.036204 | Down |
| ENSG00000168758 | SEMA4C | semaphorin_4C_[Source:HGNC_Symbol%3BAcc:HGNC:10731] | -1.17025 | 0.032977 | Down |
| ENSG00000175538 | KCNE3 | potassium_voltage-gated_channel_subfamily_E_regulatory_subunit_3_[Source:HGNC_Symbol%3BAcc:HGNC:6243] | -1.14794 | 0.043487 | Down |
| ENSG00000178896 | EXOSC4 | exosome_component_4_[Source:HGNC_Symbol%3BAcc:HGNC:18189] | -1.13699 | 0.034766 | Down |
| ENSG00000158882 | TOMM40L | translocase_of_outer_mitochondrial_membrane_40_like_[Source:HGNC_Symbol%3BAcc:HGNC:25756] | -1.06569 | 0.044225 | Down |
| ENSG00000104903 | LYL1 | LYL1_basic_helix-loop-helix_family_member_[Source:HGNC_Symbol%3BAcc:HGNC:6734] | -1.0196 | 0.047708 | Down |
| ENSG00000155629 | PIK3AP1 | phosphoinositide-3-kinase_adaptor_protein_1_[Source:HGNC_Symbol%3BAcc:HGNC:30034] | 1.01471 | 0.02051 | Up |
| ENSG00000115652 | UXS1 | UDP-glucuronate_decarboxylase_1_[Source:HGNC_Symbol%3BAcc:HGNC:17729] | 1.044753 | 0.018535 | Up |
| ENSG00000123728 | RAP2C | RAP2C%2C_member_of_RAS_oncogene_family_[Source:HGNC_Symbol%3BAcc:HGNC:21165] | 1.062256 | 0.044652 | Up |
| ENSG00000184602 | SNN | stannin_[Source:HGNC_Symbol%3BAcc:HGNC:11149] | 1.078461 | 0.044568 | Up |
| ENSG00000196470 | SIAH1 | siah_E3_ubiquitin_protein_ligase_1_[Source:HGNC_Symbol%3BAcc:HGNC:10857] | 1.09014 | 0.047561 | Up |
| ENSG00000087074 | PPP1R15A | protein_phosphatase_1_regulatory_subunit_15A_[Source:HGNC_Symbol%3BAcc:HGNC:14375] | 1.107374 | 0.025699 | Up |
| ENSG00000104825 | NFKBIB | NFKB_inhibitor_beta_[Source:HGNC_Symbol%3BAcc:HGNC:7798] | 1.110422 | 0.039163 | Up |
| ENSG00000128016 | ZFP36 | ZFP36_ring_finger_protein_[Source:HGNC_Symbol%3BAcc:HGNC:12862] | 1.116185 | 0.025939 | Up |
| ENSG00000147650 | LRP12 | LDL_receptor_related_protein_12_[Source:HGNC_Symbol%3BAcc:HGNC:31708] | 1.121553 | 0.04081 | Up |
| ENSG00000104093 | DMXL2 | Dmx_like_2_[Source:HGNC_Symbol%3BAcc:HGNC:2938] | 1.155241 | 0.022935 | Up |
| ENSG00000170142 | UBE2E1 | ubiquitin_conjugating_enzyme_E2_E1_[Source:HGNC_Symbol%3BAcc:HGNC:12477] | 1.166957 | 0.018377 | Up |
| ENSG00000102908 | NFAT5 | nuclear_factor_of_activated_T_cells_5_[Source:HGNC_Symbol%3BAcc:HGNC:7774] | 1.174417 | 0.037294 | Up |

**Table S2. Continued.**

**Table S2. Continued.**

| ENSG00000176463 | SLCO3A1 | solute_carrier_organic_anion_transporter_family_member_3A1_[Source:HGNC_Symbol%3BAcc:HGNC:10952] | 1.183584 | 0.041814 | Up |
| --- | --- | --- | --- | --- | --- |
| ENSG00000184937 | WT1 | WT1_transcription_factor_[Source:HGNC_Symbol%3BAcc:HGNC:12796] | 1.186339 | 0.044448 | Up |
| ENSG00000008128 | CDK11A | cyclin_dependent_kinase_11A_[Source:HGNC_Symbol%3BAcc:HGNC:1730] | 1.187821 | 0.046526 | Up |
| ENSG00000126561 | STAT5A | signal_transducer_and_activator_of_transcription_5A_[Source:HGNC_Symbol%3BAcc:HGNC:11366] | 1.188054 | 0.045515 | Up |
| ENSG00000154864 | PIEZO2 | piezo_type_mechanosensitive_ion_channel_component_2_[Source:HGNC_Symbol%3BAcc:HGNC:26270] | 1.20143 | 0.04411 | Up |
| ENSG00000197885 | NKIRAS1 | NFKB_inhibitor_interacting_Ras_like_1_[Source:HGNC_Symbol%3BAcc:HGNC:17899] | 1.202245 | 0.040998 | Up |
| ENSG00000160584 | SIK3 | SIK_family_kinase_3_[Source:HGNC_Symbol%3BAcc:HGNC:29165] | 1.209345 | 0.033007 | Up |
| ENSG00000139117 | CPNE8 | copine_8_[Source:HGNC_Symbol%3BAcc:HGNC:23498] | 1.221398 | 0.031966 | Up |
| ENSG00000127666 | TICAM1 | toll_like_receptor_adaptor_molecule_1_[Source:HGNC_Symbol%3BAcc:HGNC:18348] | 1.231339 | 0.022061 | Up |
| ENSG00000069702 | TGFBR3 | transforming_growth_factor_beta_receptor_3_[Source:HGNC_Symbol%3BAcc:HGNC:11774] | 1.23313 | 0.039068 | Up |
| ENSG00000137478 | FCHSD2 | FCH_and_double_SH3_domains_2_[Source:HGNC_Symbol%3BAcc:HGNC:29114] | 1.258412 | 0.031665 | Up |
| ENSG00000198121 | LPAR1 | lysophosphatidic_acid_receptor_1_[Source:HGNC_Symbol%3BAcc:HGNC:3166] | 1.263895 | 0.028284 | Up |
| ENSG00000151726 | ACSL1 | acyl-CoA_synthetase_long_chain_family_member_1_[Source:HGNC_Symbol%3BAcc:HGNC:3569] | 1.2684 | 0.019961 | Up |
| ENSG00000146278 | PNRC1 | proline_rich_nuclear_receptor_coactivator_1_[Source:HGNC_Symbol%3BAcc:HGNC:17278] | 1.271565 | 0.008356 | Up |
| ENSG00000143333 | RGS16 | regulator_of_G_protein_signaling_16_[Source:HGNC_Symbol%3BAcc:HGNC:9997] | 1.273085 | 0.01232 | Up |
| ENSG00000154265 | ABCA5 | ATP_binding_cassette_subfamily_A_member_5_[Source:HGNC_Symbol%3BAcc:HGNC:35] | 1.275508 | 0.04667 | Up |
| ENSG00000023608 | SNAPC1 | small_nuclear_RNA_activating_complex_polypeptide_1_[Source:HGNC_Symbol%3BAcc:HGNC:11134] | 1.280506 | 0.03114 | Up |
| ENSG00000154451 | GBP5 | guanylate_binding_protein_5_[Source:HGNC_Symbol%3BAcc:HGNC:19895] | 1.293134 | 0.031999 | Up |
| ENSG00000111266 | DUSP16 | dual_specificity_phosphatase_16_[Source:HGNC_Symbol%3BAcc:HGNC:17909] | 1.296432 | 0.022493 | Up |
| ENSG00000073910 | FRY | FRY_microtubule_binding_protein_[Source:HGNC_Symbol%3BAcc:HGNC:20367] | 1.304869 | 0.048328 | Up |
| ENSG00000173846 | PLK3 | polo_like_kinase_3_[Source:HGNC_Symbol%3BAcc:HGNC:2154] | 1.313469 | 0.019853 | Up |
| ENSG00000273604 | EPOP | elongin_BC_and_polycomb_repressive_complex_2_associated_protein_[Source:HGNC_Symbol%3BAcc:HGNC:34493] | 1.314459 | 0.040178 | Up |
| ENSG00000157557 | ETS2 | ETS_proto-oncogene_2%2C_transcription_factor_[Source:HGNC_Symbol%3BAcc:HGNC:3489] | 1.324929 | 0.002682 | Up |
| ENSG00000184588 | PDE4B | phosphodiesterase_4B_[Source:HGNC_Symbol%3BAcc:HGNC:8781] | 1.325115 | 0.034296 | Up |
| ENSG00000118263 | KLF7 | Kruppel_like_factor_7_[Source:HGNC_Symbol%3BAcc:HGNC:6350] | 1.338865 | 0.007354 | Up |
| ENSG00000141682 | PMAIP1 | phorbol-12-myristate-13-acetate-induced_protein_1_[Source:HGNC_Symbol%3BAcc:HGNC:9108] | 1.352579 | 0.021578 | Up |

**Table S2. Continued.**

| ENSG00000162783 | IER5 | immediate_early_response_5_[Source:HGNC_Symbol%3BAcc:HGNC:5393] | 1.357743 | 0.001915 | Up |
| --- | --- | --- | --- | --- | --- |
| ENSG00000205927 | OLIG2 | oligodendrocyte_transcription_factor_2_[Source:HGNC_Symbol%3BAcc:HGNC:9398] | 1.371616 | 0.029162 | Up |
| ENSG00000138821 | SLC39A8 | solute_carrier_family_39_member_8_[Source:HGNC_Symbol%3BAcc:HGNC:20862] | 1.406643 | 0.034947 | Up |
| ENSG00000196449 | YRDC | yrdC_N6-threonylcarbamoyltransferase_domain_containing_[Source:HGNC_Symbol%3BAcc:HGNC:28905] | 1.414155 | 0.007202 | Up |
| ENSG00000055070 | SZRD1 | SUZ_RNA_binding_domain_containing_1_[Source:HGNC_Symbol%3BAcc:HGNC:30232] | 1.414705 | 0.020626 | Up |
| ENSG00000138386 | NAB1 | NGFI-A_binding_protein_1_[Source:HGNC_Symbol%3BAcc:HGNC:7626] | 1.423877 | 0.017299 | Up |
| ENSG00000182247 | UBE2E2 | ubiquitin_conjugating_enzyme_E2_E2_[Source:HGNC_Symbol%3BAcc:HGNC:12478] | 1.425936 | 0.020586 | Up |
| ENSG00000172403 | SYNPO2 | synaptopodin_2_[Source:HGNC_Symbol%3BAcc:HGNC:17732] | 1.440036 | 0.036382 | Up |
| ENSG00000135333 | EPHA7 | EPH_receptor_A7_[Source:HGNC_Symbol%3BAcc:HGNC:3390] | 1.456142 | 0.041197 | Up |
| ENSG00000148481 | MINDY3 | MINDY_lysine_48_deubiquitinase_3_[Source:HGNC_Symbol%3BAcc:HGNC:23578] | 1.464416 | 0.018992 | Up |
| ENSG00000184221 | OLIG1 | oligodendrocyte_transcription_factor_1_[Source:HGNC_Symbol%3BAcc:HGNC:16983] | 1.469788 | 0.011997 | Up |
| ENSG00000126003 | PLAGL2 | PLAG1_like_zinc_finger_2_[Source:HGNC_Symbol%3BAcc:HGNC:9047] | 1.473446 | 0.013754 | Up |
| ENSG00000070961 | ATP2B1 | ATPase_plasma_membrane_Ca2+_transporting_1_[Source:HGNC_Symbol%3BAcc:HGNC:814] | 1.473576 | 0.010002 | Up |
| ENSG00000116044 | NFE2L2 | nuclear_factor%2C_erythroid_2_like_2_[Source:HGNC_Symbol%3BAcc:HGNC:7782] | 1.486756 | 0.002883 | Up |
| ENSG00000072694 | FCGR2B | Fc_fragment_of_IgG_receptor_IIb_[Source:HGNC_Symbol%3BAcc:HGNC:3618] | 1.490656 | 0.041709 | Up |
| ENSG00000151287 | TEX30 | testis_expressed_30_[Source:HGNC_Symbol%3BAcc:HGNC:25188] | 1.499261 | 0.005966 | Up |
| ENSG00000078795 | PKD2L2 | polycystin_2_like_2%2C_transient_receptor_potential_cation_channel_[Source:HGNC_Symbol%3BAcc:HGNC:9012] | 1.500809 | 0.041221 | Up |
| ENSG00000197147 | LRRC8B | leucine_rich_repeat_containing_8_VRAC_subunit_B_[Source:HGNC_Symbol%3BAcc:HGNC:30692] | 1.514252 | 0.01558 | Up |
| ENSG00000120280 | TASL | TLR_adaptor_interacting_with_endolysosomal_SLC15A4_[Source:HGNC_Symbol%3BAcc:HGNC:25667] | 1.52051 | 0.030047 | Up |
| ENSG00000151014 | NOCT | nocturnin_[Source:HGNC_Symbol%3BAcc:HGNC:14254] | 1.531818 | 0.027598 | Up |
| ENSG00000003402 | CFLAR | CASP8_and_FADD_like_apoptosis_regulator_[Source:HGNC_Symbol%3BAcc:HGNC:1876] | 1.541178 | 0.002236 | Up |
| ENSG00000178860 | MSC | musculin_[Source:HGNC_Symbol%3BAcc:HGNC:7321] | 1.559889 | 0.001483 | Up |
| ENSG00000110047 | EHD1 | EH_domain_containing_1_[Source:HGNC_Symbol%3BAcc:HGNC:3242] | 1.572312 | 0.010779 | Up |
| ENSG00000131323 | TRAF3 | TNF_receptor_associated_factor_3_[Source:HGNC_Symbol%3BAcc:HGNC:12033] | 1.584268 | 0.003132 | Up |
| ENSG00000164659 | ELAPOR2 | endosome-lysosome_associated_apoptosis_and_autophagy_regulator_family_member_2_[Source:HGNC_Symbol%3BAcc:HGNC:21945] | 1.585484 | 0.024358 | Up |

**Table S2. Continued.**

| ENSG00000196387 | ZNF140 | zinc_finger_protein_140_[Source:HGNC_Symbol%3BAcc:HGNC:12925] | 1.590906 | 0.022459 | Up |
| --- | --- | --- | --- | --- | --- |
| ENSG00000221866 | PLXNA4 | plexin_A4_[Source:HGNC_Symbol%3BAcc:HGNC:9102] | 1.594112 | 0.030658 | Up |
| ENSG00000165312 | OTUD1 | OTU_deubiquitinase_1_[Source:HGNC_Symbol%3BAcc:HGNC:27346] | 1.622276 | 0.021053 | Up |
| ENSG00000168389 | MFSD2A | major_facilitator_superfamily_domain_containing_2A_[Source:HGNC_Symbol%3BAcc:HGNC:25897] | 1.63011 | 0.003249 | Up |
| ENSG00000124145 | SDC4 | syndecan_4_[Source:HGNC_Symbol%3BAcc:HGNC:10661] | 1.633754 | 0.001521 | Up |
| ENSG00000169758 | TMEM266 | transmembrane_protein_266_[Source:HGNC_Symbol%3BAcc:HGNC:26763] | 1.634504 | 0.023418 | Up |
| ENSG00000160325 | CACFD1 | calcium_channel_flower_domain_containing_1_[Source:HGNC_Symbol%3BAcc:HGNC:1365] | 1.646103 | 0.001177 | Up |
| ENSG00000069399 | BCL3 | BCL3_transcription_coactivator_[Source:HGNC_Symbol%3BAcc:HGNC:998] | 1.649313 | 0.003453 | Up |
| ENSG00000149557 | FEZ1 | fasciculation_and_elongation_protein_zeta_1_[Source:HGNC_Symbol%3BAcc:HGNC:3659] | 1.649426 | 0.009702 | Up |
| ENSG00000164236 | ANKRD33B | ankyrin_repeat_domain_33B_[Source:HGNC_Symbol%3BAcc:HGNC:35240] | 1.651627 | 0.006762 | Up |
| ENSG00000122691 | TWIST1 | twist_family_bHLH_transcription_factor_1_[Source:HGNC_Symbol%3BAcc:HGNC:12428] | 1.659918 | 0.006417 | Up |
| ENSG00000177045 | SIX5 | SIX_homeobox_5_[Source:HGNC_Symbol%3BAcc:HGNC:10891] | 1.667999 | 0.007973 | Up |
| ENSG00000119866 | BCL11A | BAF_chromatin_remodeling_complex_subunit_BCL11A_[Source:HGNC_Symbol%3BAcc:HGNC:13221] | 1.66958 | 0.046585 | Up |
| ENSG00000121989 | ACVR2A | activin_A_receptor_type_2A_[Source:HGNC_Symbol%3BAcc:HGNC:173] | 1.689657 | 0.012081 | Up |
| ENSG00000188786 | MTF1 | metal_regulatory_transcription_factor_1_[Source:HGNC_Symbol%3BAcc:HGNC:7428] | 1.691991 | 0.002655 | Up |
| ENSG00000125657 | TNFSF9 | TNF_superfamily_member_9_[Source:HGNC_Symbol%3BAcc:HGNC:11939] | 1.692922 | 0.004801 | Up |
| ENSG00000145911 | N4BP3 | NEDD4_binding_protein_3_[Source:HGNC_Symbol%3BAcc:HGNC:29852] | 1.706758 | 0.029622 | Up |
| ENSG00000188559 | RALGAPA2 | Ral_GTPase_activating_protein_catalytic_subunit_alpha_2_[Source:HGNC_Symbol%3BAcc:HGNC:16207] | 1.707311 | 0.019085 | Up |
| ENSG00000145901 | TNIP1 | TNFAIP3_interacting_protein_1_[Source:HGNC_Symbol%3BAcc:HGNC:16903] | 1.715834 | 0.000554 | Up |
| ENSG00000167604 | NFKBID | NFKB_inhibitor_delta_[Source:HGNC_Symbol%3BAcc:HGNC:15671] | 1.725186 | 0.021361 | Up |
| ENSG00000185022 | MAFF | MAF_bZIP_transcription_factor_F_[Source:HGNC_Symbol%3BAcc:HGNC:6780] | 1.726227 | 0.044104 | Up |
| ENSG00000089351 | GRAMD1A | GRAM_domain_containing_1A_[Source:HGNC_Symbol%3BAcc:HGNC:29305] | 1.726706 | 0.000933 | Up |
| ENSG00000113504 | SLC12A7 | solute_carrier_family_12_member_7_[Source:HGNC_Symbol%3BAcc:HGNC:10915] | 1.738025 | 0.003385 | Up |
| ENSG00000134138 | MEIS2 | Meis_homeobox_2_[Source:HGNC_Symbol%3BAcc:HGNC:7001] | 1.753881 | 0.027011 | Up |
| ENSG00000154640 | BTG3 | BTG_anti-proliferation_factor_3_[Source:HGNC_Symbol%3BAcc:HGNC:1132] | 1.780904 | 0.000993 | Up |
| ENSG00000137872 | SEMA6D | semaphorin_6D_[Source:HGNC_Symbol%3BAcc:HGNC:16770] | 1.783443 | 0.017553 | Up |

| ENSG00000180509 | KCNE1 | potassium_voltage-gated_channel_subfamily_E_regulatory_subunit_1_[Source:HGNC_Symbol%3BAcc:HGNC:6240] | 1.797878 | 0.028018 | Up |
| --- | --- | --- | --- | --- | --- |
| ENSG00000102096 | PIM2 | Pim-2_proto-oncogene%2C_serine/threonine_kinase_[Source:HGNC_Symbol%3BAcc:HGNC:8987] | 1.819769 | 0.001261 | Up |
| ENSG00000125845 | BMP2 | bone_morphogenetic_protein_2_[Source:HGNC_Symbol%3BAcc:HGNC:1069] | 1.844102 | 0.013432 | Up |
| ENSG00000113916 | BCL6 | BCL6_transcription_repressor_[Source:HGNC_Symbol%3BAcc:HGNC:1001] | 1.866642 | 0.005923 | Up |
| ENSG00000141510 | TP53 | tumor_protein_p53_[Source:HGNC_Symbol%3BAcc:HGNC:11998] | 1.870237 | 0.023764 | Up |
| ENSG00000152484 | USP12 | ubiquitin_specific_peptidase_12_[Source:HGNC_Symbol%3BAcc:HGNC:20485] | 1.886832 | 0.000601 | Up |
| ENSG00000138670 | RASGEF1B | RasGEF_domain_family_member_1B_[Source:HGNC_Symbol%3BAcc:HGNC:24881] | 1.890361 | 0.017224 | Up |
| ENSG00000265190 | ANXA8 | annexin_A8_[Source:HGNC_Symbol%3BAcc:HGNC:546] | 1.902489 | 0.048497 | Up |
| ENSG00000152229 | PSTPIP2 | proline-serine-threonine_phosphatase_interacting_protein_2_[Source:HGNC_Symbol%3BAcc:HGNC:9581] | 1.919964 | 0.009333 | Up |
| ENSG00000186431 | FCAR | Fc_fragment_of_IgA_receptor_[Source:HGNC_Symbol%3BAcc:HGNC:3608] | 1.923927 | 0.018676 | Up |
| ENSG00000160716 | CHRNB2 | cholinergic_receptor_nicotinic_beta_2_subunit_[Source:HGNC_Symbol%3BAcc:HGNC:1962] | 1.95279 | 0.0251 | Up |
| ENSG00000165246 | NLGN4Y | neuroligin_4_Y-linked_[Source:HGNC_Symbol%3BAcc:HGNC:15529] | 1.955424 | 0.000746 | Up |
| ENSG00000198355 | PIM3 | Pim-3_proto-oncogene%2C_serine/threonine_kinase_[Source:HGNC_Symbol%3BAcc:HGNC:19310] | 1.957004 | 0.000162 | Up |
| ENSG00000104312 | RIPK2 | receptor_interacting_serine/threonine_kinase_2_[Source:HGNC_Symbol%3BAcc:HGNC:10020] | 1.957347 | 0.001175 | Up |
| ENSG00000153234 | NR4A2 | nuclear_receptor_subfamily_4_group_A_member_2_[Source:HGNC_Symbol%3BAcc:HGNC:7981] | 1.960005 | 0.008828 | Up |
| ENSG00000099834 | CDHR5 | cadherin_related_family_member_5_[Source:HGNC_Symbol%3BAcc:HGNC:7521] | 1.966415 | 0.032854 | Up |
| ENSG00000196092 | PAX5 | paired_box_5_[Source:HGNC_Symbol%3BAcc:HGNC:8619] | 1.996704 | 0.039634 | Up |
| ENSG00000050344 | NFE2L3 | nuclear_factor%2C_erythroid_2_like_3_[Source:HGNC_Symbol%3BAcc:HGNC:7783] | 2.013296 | 0.031604 | Up |
| ENSG00000164989 | CCDC171 | coiled-coil_domain_containing_171_[Source:HGNC_Symbol%3BAcc:HGNC:29828] | 2.013883 | 0.044582 | Up |
| ENSG00000171724 | VAT1L | vesicle_amine_transport_1_like_[Source:HGNC_Symbol%3BAcc:HGNC:29315] | 2.015501 | 0.041025 | Up |
| ENSG00000111912 | NCOA7 | nuclear_receptor_coactivator_7_[Source:HGNC_Symbol%3BAcc:HGNC:21081] | 2.024013 | 0.001653 | Up |
| ENSG00000196220 | SRGAP3 | SLIT-ROBO_Rho_GTPase_activating_protein_3_[Source:HGNC_Symbol%3BAcc:HGNC:19744] | 2.025466 | 0.021899 | Up |
| ENSG00000077150 | NFKB2 | nuclear_factor_kappa_B_subunit_2_[Source:HGNC_Symbol%3BAcc:HGNC:7795] | 2.04573 | 0.001845 | Up |
| ENSG00000160326 | SLC2A6 | solute_carrier_family_2_member_6_[Source:HGNC_Symbol%3BAcc:HGNC:11011] | 2.052637 | 0.0000298 | Up |
| ENSG00000143067 | ZNF697 | zinc_finger_protein_697_[Source:HGNC_Symbol%3BAcc:HGNC:32034] | 2.059033 | 0.000972 | Up |
| ENSG00000116667 | C1orf21 | chromosome_1_open_reading_frame_21_[Source:HGNC_Symbol%3BAcc:HGNC:15494] | 2.090431 | 0.003492 | Up |

**Table S2. Continued.**

| ENSG00000154359 | LONRF1 | LON_peptidase_N-terminal_domain_and_ring_finger_1_[Source:HGNC_Symbol%3BAcc:HGNC:26302] | 2.097391 | 0.002613 | Up |
| --- | --- | --- | --- | --- | --- |
| ENSG00000154639 | CXADR | CXADR_Ig-like_cell_adhesion_molecule_[Source:HGNC_Symbol%3BAcc:HGNC:2559] | 2.12204 | 0.04027 | Up |
| ENSG00000112149 | CD83 | CD83_molecule_[Source:HGNC_Symbol%3BAcc:HGNC:1703] | 2.128192 | 0.000776 | Up |
| ENSG00000145632 | PLK2 | polo_like_kinase_2_[Source:HGNC_Symbol%3BAcc:HGNC:19699] | 2.129361 | 0.005156 | Up |
| ENSG00000126016 | AMOT | angiomotin_[Source:HGNC_Symbol%3BAcc:HGNC:17810] | 2.140132 | 0.024195 | Up |
| ENSG00000188396 | DYNLT4 | dynein_light_chain_Tctex-type_4_[Source:HGNC_Symbol%3BAcc:HGNC:32315] | 2.143097 | 0.025403 | Up |
| ENSG00000172817 | CYP7B1 | cytochrome_P450_family_7_subfamily_B_member_1_[Source:HGNC_Symbol%3BAcc:HGNC:2652] | 2.156688 | 0.002967 | Up |
| ENSG00000153898 | MCOLN2 | mucolipin_TRP_cation_channel_2_[Source:HGNC_Symbol%3BAcc:HGNC:13357] | 2.171031 | 0.009929 | Up |
| ENSG00000100906 | NFKBIA | NFKB_inhibitor_alpha_[Source:HGNC_Symbol%3BAcc:HGNC:7797] | 2.175284 | 0.00000403 | Up |
| ENSG00000101017 | CD40 | CD40_molecule_[Source:HGNC_Symbol%3BAcc:HGNC:11919] | 2.18301 | 0.000197 | Up |
| ENSG00000162645 | GBP2 | guanylate_binding_protein_2_[Source:HGNC_Symbol%3BAcc:HGNC:4183] | 2.187296 | 0.001202 | Up |
| ENSG00000159388 | BTG2 | BTG_anti-proliferation_factor_2_[Source:HGNC_Symbol%3BAcc:HGNC:1131] | 2.197168 | 0.0000273 | Up |
| ENSG00000104856 | RELB | RELB_proto-oncogene%2C_NF-kB_subunit_[Source:HGNC_Symbol%3BAcc:HGNC:9956] | 2.197676 | 0.000727 | Up |
| ENSG00000145365 | TIFA | TRAF_interacting_protein_with_forkhead_associated_domain_[Source:HGNC_Symbol%3BAcc:HGNC:19075] | 2.204442 | 0.001208 | Up |
| ENSG00000164418 | GRIK2 | glutamate_ionotropic_receptor_kainate_type_subunit_2_[Source:HGNC_Symbol%3BAcc:HGNC:4580] | 2.20464 | 0.007496 | Up |
| ENSG00000107242 | PIP5K1B | phosphatidylinositol-4-phosphate_5-kinase_type_1_beta_[Source:HGNC_Symbol%3BAcc:HGNC:8995] | 2.215206 | 0.007457 | Up |
| ENSG00000110844 | PRPF40B | pre-mRNA_processing_factor_40_homolog_B_[Source:HGNC_Symbol%3BAcc:HGNC:25031] | 2.216857 | 0.010434 | Up |
| ENSG00000186654 | PRR5 | proline_rich_5_[Source:HGNC_Symbol%3BAcc:HGNC:31682] | 2.218052 | 0.01311 | Up |
| ENSG00000168994 | PXDC1 | PX_domain_containing_1_[Source:HGNC_Symbol%3BAcc:HGNC:21361] | 2.219445 | 0.015355 | Up |
| ENSG00000173517 | PEAK1 | pseudopodium_enriched_atypical_kinase_1_[Source:HGNC_Symbol%3BAcc:HGNC:29431] | 2.249424 | 0.002873 | Up |
| ENSG00000155324 | GRAMD2B | GRAM_domain_containing_2B_[Source:HGNC_Symbol%3BAcc:HGNC:24911] | 2.252765 | 0.000403 | Up |
| ENSG00000162643 | DNAI3 | dynein_axonemal_intermediate_chain_3_[Source:HGNC_Symbol%3BAcc:HGNC:30711] | 2.26076 | 0.01364 | Up |
| ENSG00000090339 | ICAM1 | intercellular_adhesion_molecule_1_[Source:HGNC_Symbol%3BAcc:HGNC:5344] | 2.27624 | 0.000971 | Up |
| ENSG00000112394 | SLC16A10 | solute_carrier_family_16_member_10_[Source:HGNC_Symbol%3BAcc:HGNC:17027] | 2.282516 | 0.018971 | Up |
| ENSG00000139194 | RBP5 | retinol_binding_protein_5_[Source:HGNC_Symbol%3BAcc:HGNC:15847] | 2.291891 | 0.043024 | Up |

**Table S2. Continued.**

**Table S2. Continued.**

| ENSG00000134762 | DSC3 | desmocollin_3_[Source:HGNC_Symbol%3BAcc:HGNC:3037] | 2.307174 | 0.005549 | Up |
| --- | --- | --- | --- | --- | --- |
| ENSG00000162924 | REL | REL_proto-oncogene%2C_NF-kB_subunit_[Source:HGNC_Symbol%3BAcc:HGNC:9954] | 2.30936 | 0.0000443 | Up |
| ENSG00000107968 | MAP3K8 | mitogen-activated_protein_kinase_kinase_kinase_8_[Source:HGNC_Symbol%3BAcc:HGNC:6860] | 2.320718 | 0.000387 | Up |
| ENSG00000089123 | TASP1 | taspase_1_[Source:HGNC_Symbol%3BAcc:HGNC:15859] | 2.340206 | 0.041259 | Up |
| ENSG00000144935 | TRPC1 | transient_receptor_potential_cation_channel_subfamily_C_member_1_[Source:HGNC_Symbol%3BAcc:HGNC:12333] | 2.342501 | 0.03323 | Up |
| ENSG00000108691 | CCL2 | C-C_motif_chemokine_ligand_2_[Source:HGNC_Symbol%3BAcc:HGNC:10618] | 2.347438 | 0.005251 | Up |
| ENSG00000099992 | TBC1D10A | TBC1_domain_family_member_10A_[Source:HGNC_Symbol%3BAcc:HGNC:23609] | 2.358657 | 0.006992 | Up |
| ENSG00000111863 | ADTRP | androgen_dependent_TFPI_regulating_protein_[Source:HGNC_Symbol%3BAcc:HGNC:21214] | 2.368754 | 0.010714 | Up |
| ENSG00000185215 | TNFAIP2 | TNF_alpha_induced_protein_2_[Source:HGNC_Symbol%3BAcc:HGNC:11895] | 2.371781 | 0.0000297 | Up |
| ENSG00000095951 | HIVEP1 | HIVEP_zinc_finger_1_[Source:HGNC_Symbol%3BAcc:HGNC:4920] | 2.392501 | 0.001714 | Up |
| ENSG00000266094 | RASSF5 | Ras_association_domain_family_member_5_[Source:HGNC_Symbol%3BAcc:HGNC:17609] | 2.416107 | 0.000286 | Up |
| ENSG00000154734 | ADAMTS1 | ADAM_metallopeptidase_with_thrombospondin_type_1_motif_1_[Source:HGNC_Symbol%3BAcc:HGNC:217] | 2.426405 | 0.002246 | Up |
| ENSG00000135604 | STX11 | syntaxin_11_[Source:HGNC_Symbol%3BAcc:HGNC:11429] | 2.443069 | 0.0000613 | Up |
| ENSG00000186088 | GSAP | gamma-secretase_activating_protein_[Source:HGNC_Symbol%3BAcc:HGNC:28042] | 2.455527 | 0.000477 | Up |
| ENSG00000203727 | SAMD5 | sterile_alpha_motif_domain_containing_5_[Source:HGNC_Symbol%3BAcc:HGNC:21180] | 2.466169 | 0.024893 | Up |
| ENSG00000168421 | RHOH | ras_homolog_family_member_H_[Source:HGNC_Symbol%3BAcc:HGNC:686] | 2.501948 | 0.000645 | Up |
| ENSG00000117525 | F3 | coagulation_factor_III%2C_tissue_factor_[Source:HGNC_Symbol%3BAcc:HGNC:3541] | 2.566615 | 0.024015 | Up |
| ENSG00000136048 | DRAM1 | DNA_damage_regulated_autophagy_modulator_1_[Source:HGNC_Symbol%3BAcc:HGNC:25645] | 2.590465 | 0.0000149 | Up |
| ENSG00000132510 | KDM6B | lysine_demethylase_6B_[Source:HGNC_Symbol%3BAcc:HGNC:29012] | 2.59954 | 0.000165 | Up |
| ENSG00000164330 | EBF1 | EBF_transcription_factor_1_[Source:HGNC_Symbol%3BAcc:HGNC:3126] | 2.599614 | 0.004516 | Up |
| ENSG00000108984 | MAP2K6 | mitogen-activated_protein_kinase_kinase_6_[Source:HGNC_Symbol%3BAcc:HGNC:6846] | 2.607801 | 0.047775 | Up |
| ENSG00000137203 | TFAP2A | transcription_factor_AP-2_alpha_[Source:HGNC_Symbol%3BAcc:HGNC:11742] | 2.621688 | 0.001611 | Up |
| ENSG00000109320 | NFKB1 | nuclear_factor_kappa_B_subunit_1_[Source:HGNC_Symbol%3BAcc:HGNC:7794] | 2.671925 | 0.0000402 | Up |
| ENSG00000188992 | LIPI | lipase_I_[Source:HGNC_Symbol%3BAcc:HGNC:18821] | 2.680496 | 0.001536 | Up |
| ENSG00000158050 | DUSP2 | dual_specificity_phosphatase_2_[Source:HGNC_Symbol%3BAcc:HGNC:3068] | 2.738626 | 0.000262 | Up |
| ENSG00000205189 | ZBTB10 | zinc_finger_and_BTB_domain_containing_10_[Source:HGNC_Symbol%3BAcc:HGNC:30953] | 2.740342 | 0.0000397 | Up |

| ENSG00000184557 | SOCS3 | suppressor_of_cytokine_signaling_3_[Source:HGNC_Symbol%3BAcc:HGNC:19391] | 2.761846 | 0.0000886 | Up |
| --- | --- | --- | --- | --- | --- |
| ENSG00000134901 | POGLUT2 | protein_O-glucosyltransferase_2_[Source:HGNC_Symbol%3BAcc:HGNC:19350] | 2.770617 | 0.046238 | Up |
| ENSG00000211445 | GPX3 | glutathione_peroxidase_3_[Source:HGNC_Symbol%3BAcc:HGNC:4555] | 2.812177 | 0.00119 | Up |
| ENSG00000134571 | MYBPC3 | myosin_binding_protein_C3_[Source:HGNC_Symbol%3BAcc:HGNC:7551] | 2.812385 | 0.001473 | Up |
| ENSG00000059915 | PSD | pleckstrin_and_Sec7_domain_containing_[Source:HGNC_Symbol%3BAcc:HGNC:9507] | 2.851878 | 0.015303 | Up |
| ENSG00000146674 | IGFBP3 | insulin_like_growth_factor_binding_protein_3_[Source:HGNC_Symbol%3BAcc:HGNC:5472] | 2.935714 | 0.000125 | Up |
| ENSG00000232810 | TNF | tumor_necrosis_factor_[Source:HGNC_Symbol%3BAcc:HGNC:11892] | 2.939463 | 0.006363 | Up |
| ENSG00000176381 | PRR18 | proline_rich_18_[Source:HGNC_Symbol%3BAcc:HGNC:28574] | 2.939974 | 0.027664 | Up |
| ENSG00000042062 | RIPOR3 | RIPOR_family_member_3_[Source:HGNC_Symbol%3BAcc:HGNC:16168] | 2.941607 | 0.004019 | Up |
| ENSG00000147509 | RGS20 | regulator_of_G_protein_signaling_20_[Source:HGNC_Symbol%3BAcc:HGNC:14600] | 2.949385 | 0.02043 | Up |
| ENSG00000214113 | LYRM4 | LYR_motif_containing_4_[Source:HGNC_Symbol%3BAcc:HGNC:21365] | 2.965379 | 0.000125 | Up |
| ENSG00000187950 | OVCH1 | ovochymase_1_[Source:HGNC_Symbol%3BAcc:HGNC:23080] | 2.984517 | 0.000842 | Up |
| ENSG00000255330 |  | novel__protein | 3.01634 | 0.046248 | Up |
| ENSG00000175471 | MCTP1 | multiple_C2_and_transmembrane_domain_containing_1_[Source:HGNC_Symbol%3BAcc:HGNC:26183] | 3.021253 | 0.000198 | Up |
| ENSG00000125384 | PTGER2 | prostaglandin_E_receptor_2_[Source:HGNC_Symbol%3BAcc:HGNC:9594] | 3.064429 | 0.000305 | Up |
| ENSG00000122877 | EGR2 | early_growth_response_2_[Source:HGNC_Symbol%3BAcc:HGNC:3239] | 3.067382 | 0.001278 | Up |
| ENSG00000105246 | EBI3 | Epstein-Barr_virus_induced_3_[Source:HGNC_Symbol%3BAcc:HGNC:3129] | 3.075928 | 0.000193 | Up |
| ENSG00000123700 | KCNJ2 | potassium_inwardly_rectifying_channel_subfamily_J_member_2_[Source:HGNC_Symbol%3BAcc:HGNC:6263] | 3.079767 | 0.000142 | Up |
| ENSG00000134070 | IRAK2 | interleukin_1_receptor_associated_kinase_2_[Source:HGNC_Symbol%3BAcc:HGNC:6113] | 3.082804 | 0.0000151 | Up |
| ENSG00000067798 | NAV3 | neuron_navigator_3_[Source:HGNC_Symbol%3BAcc:HGNC:15998] | 3.087668 | 0.0000241 | Up |
| ENSG00000166016 | ABTB2 | ankyrin_repeat_and_BTB_domain_containing_2_[Source:HGNC_Symbol%3BAcc:HGNC:23842] | 3.088999 | 0.00000318 | Up |
| ENSG00000164949 | GEM | GTP_binding_protein_overexpressed_in_skeletal_muscle_[Source:HGNC_Symbol%3BAcc:HGNC:4234] | 3.162258 | 0.000107 | Up |
| ENSG00000131979 | GCH1 | GTP_cyclohydrolase_1_[Source:HGNC_Symbol%3BAcc:HGNC:4193] | 3.181149 | 0.00014 | Up |
| ENSG00000118514 | ALDH8A1 | aldehyde_dehydrogenase_8_family_member_A1_[Source:HGNC_Symbol%3BAcc:HGNC:15471] | 3.208477 | 0.041275 | Up |
| ENSG00000102302 | FGD1 | FYVE%2C_RhoGEF_and_PH_domain_containing_1_[Source:HGNC_Symbol%3BAcc:HGNC:3663] | 3.2085 | 0.041307 | Up |
| ENSG00000138685 | FGF2 | fibroblast_growth_factor_2_[Source:HGNC_Symbol%3BAcc:HGNC:3676] | 3.255682 | 0.009354 | Up |

**Table S2. Continued.**

| ENSG00000137331 | IER3 | immediate_early_response_3_[Source:HGNC_Symbol%3BAcc:HGNC:5392] | 3.27185 | 0.0000527 | Up |
| --- | --- | --- | --- | --- | --- |
| ENSG00000164603 | BMT2 | base_methyltransferase_of_25S_rRNA_2_homolog_[Source:HGNC_Symbol%3BAcc:HGNC:26475] | 3.27637 | 0.000103 | Up |
| ENSG00000010818 | HIVEP2 | HIVEP_zinc_finger_2_[Source:HGNC_Symbol%3BAcc:HGNC:4921] | 3.277158 | 0.000382 | Up |
| ENSG00000115919 | KYNU | kynureninase_[Source:HGNC_Symbol%3BAcc:HGNC:6469] | 3.285085 | 0.00000731 | Up |
| ENSG00000224420 | ADM5 | adrenomedullin_5_(putative)_[Source:HGNC_Symbol%3BAcc:HGNC:27293] | 3.310352 | 0.029195 | Up |
| ENSG00000076554 | TPD52 | tumor_protein_D52_[Source:HGNC_Symbol%3BAcc:HGNC:12005] | 3.358826 | 0.00501 | Up |
| ENSG00000187122 | SLIT1 | slit_guidance_ligand_1_[Source:HGNC_Symbol%3BAcc:HGNC:11085] | 3.410385 | 0.025327 | Up |
| ENSG00000163874 | ZC3H12A | zinc_finger_CCCH-type_containing_12A_[Source:HGNC_Symbol%3BAcc:HGNC:26259] | 3.430029 | 3.53E-13 | Up |
| ENSG00000184545 | DUSP8 | dual_specificity_phosphatase_8_[Source:HGNC_Symbol%3BAcc:HGNC:3074] | 3.439059 | 0.0000287 | Up |
| ENSG00000164181 | ELOVL7 | ELOVL_fatty_acid_elongase_7_[Source:HGNC_Symbol%3BAcc:HGNC:26292] | 3.442037 | 0.000271 | Up |
| ENSG00000128917 | DLL4 | delta_like_canonical_Notch_ligand_4_[Source:HGNC_Symbol%3BAcc:HGNC:2910] | 3.467946 | 2.23E-07 | Up |
| ENSG00000112096 | SOD2 | superoxide_dismutase_2_[Source:HGNC_Symbol%3BAcc:HGNC:11180] | 3.515845 | 7.28E-09 | Up |
| ENSG00000145779 | TNFAIP8 | TNF_alpha_induced_protein_8_[Source:HGNC_Symbol%3BAcc:HGNC:17260] | 3.518319 | 2.47E-08 | Up |
| ENSG00000056558 | TRAF1 | TNF_receptor_associated_factor_1_[Source:HGNC_Symbol%3BAcc:HGNC:12031] | 3.540648 | 0.0000125 | Up |
| ENSG00000055813 | CCDC85A | coiled-coil_domain_containing_85A_[Source:HGNC_Symbol%3BAcc:HGNC:29400] | 3.545576 | 0.0037 | Up |
| ENSG00000112246 | SIM1 | SIM_bHLH_transcription_factor_1_[Source:HGNC_Symbol%3BAcc:HGNC:10882] | 3.592003 | 0.033293 | Up |
| ENSG00000288658 |  | novel_protein%2C_ortholog_of_Gm2102_(M._musculus) | 3.677509 | 0.0000138 | Up |
| ENSG00000091129 | NRCAM | neuronal_cell_adhesion_molecule_[Source:HGNC_Symbol%3BAcc:HGNC:7994] | 3.724162 | 0.005666 | Up |
| ENSG00000121594 | CD80 | CD80_molecule_[Source:HGNC_Symbol%3BAcc:HGNC:1700] | 3.763553 | 0.001762 | Up |
| ENSG00000138771 | SHROOM3 | shroom_family_member_3_[Source:HGNC_Symbol%3BAcc:HGNC:30422] | 3.833553 | 0.003596 | Up |
| ENSG00000073282 | TP63 | tumor_protein_p63_[Source:HGNC_Symbol%3BAcc:HGNC:15979] | 3.839463 | 0.005768 | Up |
| ENSG00000120738 | EGR1 | early_growth_response_1_[Source:HGNC_Symbol%3BAcc:HGNC:3238] | 3.889525 | 0.001385 | Up |
| ENSG00000137507 | LRRC32 | leucine_rich_repeat_containing_32_[Source:HGNC_Symbol%3BAcc:HGNC:4161] | 3.926699 | 0.014563 | Up |
| ENSG00000155011 | DKK2 | dickkopf_WNT_signaling_pathway_inhibitor_2_[Source:HGNC_Symbol%3BAcc:HGNC:2892] | 3.950479 | 0.002287 | Up |
| ENSG00000118094 | TREH | trehalase_[Source:HGNC_Symbol%3BAcc:HGNC:12266] | 4.028507 | 0.014128 | Up |
| ENSG00000277632 | CCL3 | C-C_motif_chemokine_ligand_3_[Source:HGNC_Symbol%3BAcc:HGNC:10627] | 4.095059 | 0.00000131 | Up |

**Table S2. Continued.**

| ENSG00000123358 | NR4A1 | nuclear_receptor_subfamily_4_group_A_member_1_[Source:HGNC_Symbol%3BAcc:HGNC:7980] | 4.1371 | 0.007678 | Up |
| --- | --- | --- | --- | --- | --- |
| ENSG00000276085 | CCL3L1 | C-C_motif_chemokine_ligand_3_like_1_[Source:HGNC_Symbol%3BAcc:HGNC:10628] | 4.254261 | 0.00000733 | Up |
| ENSG00000112183 | RBM24 | RNA_binding_motif_protein_24_[Source:HGNC_Symbol%3BAcc:HGNC:21539] | 4.273524 | 0.009366 | Up |
| ENSG00000148459 | PDSS1 | decaprenyl_diphosphate_synthase_subunit_1_[Source:HGNC_Symbol%3BAcc:HGNC:17759] | 4.294966 | 0.00000809 | Up |
| ENSG00000125538 | IL1B | interleukin_1_beta_[Source:HGNC_Symbol%3BAcc:HGNC:5992] | 4.295772 | 1.39E-07 | Up |
| ENSG00000254979 |  | novel_protein | 4.433839 | 0.04587 | Up |
| ENSG00000110944 | IL23A | interleukin_23_subunit_alpha_[Source:HGNC_Symbol%3BAcc:HGNC:15488] | 4.528044 | 0.00000235 | Up |
| ENSG00000260371 |  | novel_protein | 4.60213 | 0.025367 | Up |
| ENSG00000164619 | BMPER | BMP_binding_endothelial_regulator_[Source:HGNC_Symbol%3BAcc:HGNC:24154] | 4.657126 | 0.000457 | Up |
| ENSG00000138639 | ARHGAP24 | Rho_GTPase_activating_protein_24_[Source:HGNC_Symbol%3BAcc:HGNC:25361] | 4.703539 | 0.013255 | Up |
| ENSG00000101115 | SALL4 | spalt_like_transcription_factor_4_[Source:HGNC_Symbol%3BAcc:HGNC:15924] | 4.715071 | 0.029481 | Up |
| ENSG00000168032 | ENTPD3 | ectonucleoside_triphosphate_diphosphohydrolase_3_[Source:HGNC_Symbol%3BAcc:HGNC:3365] | 4.730881 | 0.032167 | Up |
| ENSG00000258989 |  | novel_protein | 4.730888 | 0.032144 | Up |
| ENSG00000258461 |  | novel_transcript | 4.799943 | 0.038499 | Up |
| ENSG00000138378 | STAT4 | signal_transducer_and_activator_of_transcription_4_[Source:HGNC_Symbol%3BAcc:HGNC:11365] | 4.813428 | 0.000602 | Up |
| ENSG00000227051 | C14orf132 | chromosome_14_open_reading_frame_132_[Source:HGNC_Symbol%3BAcc:HGNC:20346] | 4.817856 | 0.03769 | Up |
| ENSG00000180611 | MB21D2 | Mab-21_domain_containing_2_[Source:HGNC_Symbol%3BAcc:HGNC:30438] | 4.828879 | 0.013137 | Up |
| ENSG00000100311 | PDGFB | platelet_derived_growth_factor_subunit_B_[Source:HGNC_Symbol%3BAcc:HGNC:8800] | 4.829739 | 0.040927 | Up |
| ENSG00000150630 | VEGFC | vascular_endothelial_growth_factor_C_[Source:HGNC_Symbol%3BAcc:HGNC:12682] | 4.831742 | 0.0000563 | Up |
| ENSG00000115009 | CCL20 | C-C_motif_chemokine_ligand_20_[Source:HGNC_Symbol%3BAcc:HGNC:10619] | 4.872348 | 1.76E-09 | Up |
| ENSG00000118503 | TNFAIP3 | TNF_alpha_induced_protein_3_[Source:HGNC_Symbol%3BAcc:HGNC:11896] | 4.881631 | 6.6E-15 | Up |
| ENSG00000077274 | CAPN6 | calpain_6_[Source:HGNC_Symbol%3BAcc:HGNC:1483] | 4.927322 | 0.03267 | Up |
| ENSG00000073756 | PTGS2 | prostaglandin-endoperoxide_synthase_2_[Source:HGNC_Symbol%3BAcc:HGNC:9605] | 4.931173 | 0.00000534 | Up |
| ENSG00000137033 | IL33 | interleukin_33_[Source:HGNC_Symbol%3BAcc:HGNC:16028] | 4.994021 | 0.007508 | Up |
| ENSG00000105711 | SCN1B | sodium_voltage-gated_channel_beta_subunit_1_[Source:HGNC_Symbol%3BAcc:HGNC:10586] | 5.009537 | 0.008292 | Up |
| ENSG00000117090 | SLAMF1 | signaling_lymphocytic_activation_molecule_family_member_1_[Source:HGNC_Symbol%3BAcc:HGNC:10903] | 5.064546 | 0.000106 | Up |

**Table S2. Continued.**

| ENSG00000118523 | CCN2 | cellular_communication_network_factor_2_[Source:HGNC_Symbol%3BAcc:HGNC:2500] | 5.070689 | 6.54E-09 | Up |
| --- | --- | --- | --- | --- | --- |
| ENSG00000078081 | LAMP3 | lysosomal_associated_membrane_protein_3_[Source:HGNC_Symbol%3BAcc:HGNC:14582] | 5.08719 | 0.000681 | Up |
| ENSG00000214324 | C3orf56 | chromosome_3_open_reading_frame_56_[Source:HGNC_Symbol%3BAcc:HGNC:32481] | 5.096015 | 0.028926 | Up |
| ENSG00000197191 | CYSRT1 | cysteine_rich_tail_1_[Source:HGNC_Symbol%3BAcc:HGNC:30529] | 5.107938 | 0.010322 | Up |
| ENSG00000092295 | TGM1 | transglutaminase_1_[Source:HGNC_Symbol%3BAcc:HGNC:11777] | 5.130462 | 0.006548 | Up |
| ENSG00000023445 | BIRC3 | baculoviral_IAP_repeat_containing_3_[Source:HGNC_Symbol%3BAcc:HGNC:591] | 5.188055 | 1.36E-10 | Up |
| ENSG00000054598 | FOXC1 | forkhead_box_C1_[Source:HGNC_Symbol%3BAcc:HGNC:3800] | 5.226044 | 0.005014 | Up |
| ENSG00000150938 | CRIM1 | cysteine_rich_transmembrane_BMP_regulator_1_[Source:HGNC_Symbol%3BAcc:HGNC:2359] | 5.386505 | 5.91E-09 | Up |
| ENSG00000138650 | PCDH10 | protocadherin_10_[Source:HGNC_Symbol%3BAcc:HGNC:13404] | 5.419375 | 0.015557 | Up |
| ENSG00000280228 |  | TEC | 5.420458 | 0.00784 | Up |
| ENSG00000122641 | INHBA | inhibin_subunit_beta_A_[Source:HGNC_Symbol%3BAcc:HGNC:6066] | 5.448518 | 0.034232 | Up |
| ENSG00000169429 | CXCL8 | C-X-C_motif_chemokine_ligand_8_[Source:HGNC_Symbol%3BAcc:HGNC:6025] | 5.454349 | 2.72E-26 | Up |
| ENSG00000186891 | TNFRSF18 | TNF_receptor_superfamily_member_18_[Source:HGNC_Symbol%3BAcc:HGNC:11914] | 5.454543 | 0.032564 | Up |
| ENSG00000275302 | CCL4 | C-C_motif_chemokine_ligand_4_[Source:HGNC_Symbol%3BAcc:HGNC:10630] | 5.616571 | 8.97E-09 | Up |
| ENSG00000182050 | MGAT4C | MGAT4_family_member_C_[Source:HGNC_Symbol%3BAcc:HGNC:30871] | 5.649153 | 0.027867 | Up |
| ENSG00000144802 | NFKBIZ | NFKB_inhibitor_zeta_[Source:HGNC_Symbol%3BAcc:HGNC:29805] | 5.741557 | 4.57E-20 | Up |
| ENSG00000214511 | HIGD1C | HIG1_hypoxia_inducible_domain_family_member_1C_[Source:HGNC_Symbol%3BAcc:HGNC:28044] | 5.876351 | 0.002827 | Up |
| ENSG00000137265 | IRF4 | interferon_regulatory_factor_4_[Source:HGNC_Symbol%3BAcc:HGNC:6119] | 5.942709 | 0.011756 | Up |
| ENSG00000276070 | CCL4L2 | C-C_motif_chemokine_ligand_4_like_2_[Source:HGNC_Symbol%3BAcc:HGNC:24066] | 5.977548 | 7.72E-07 | Up |
| ENSG00000137393 | RNF144B | ring_finger_protein_144B_[Source:HGNC_Symbol%3BAcc:HGNC:21578] | 6.005668 | 0.00000199 | Up |
| ENSG00000136244 | IL6 | interleukin_6_[Source:HGNC_Symbol%3BAcc:HGNC:6018] | 6.202526 | 0.000151 | Up |
| ENSG00000276418 |  | novel_protein%2C_TPD52-MRPS28_readthrough | 6.288541 | 0.005905 | Up |
| ENSG00000279212 |  | TEC | 6.33501 | 0.009339 | Up |
| ENSG00000175356 | SCUBE2 | signal_peptide%2C_CUB_domain_and_EGF_like_domain_containing_2_[Source:HGNC_Symbol%3BAcc:HGNC:30425] | 6.368477 | 0.023687 | Up |
| ENSG00000144810 | COL8A1 | collagen_type_VIII_alpha_1_chain_[Source:HGNC_Symbol%3BAcc:HGNC:2215] | 6.368482 | 0.023714 | Up |
| ENSG00000115607 | IL18RAP | interleukin_18_receptor_accessory_protein_[Source:HGNC_Symbol%3BAcc:HGNC:5989] | 6.474966 | 0.020406 | Up |

**Table S2. Continued.**

| ENSG00000127954 | STEAP4 | STEAP4_metalloreductase_[Source:HGNC_Symbol%3BAcc:HGNC:21923] | 6.525367 | 0.019253 | Up |
| --- | --- | --- | --- | --- | --- |
| ENSG00000119508 | NR4A3 | nuclear_receptor_subfamily_4_group_A_member_3_[Source:HGNC_Symbol%3BAcc:HGNC:7982] | 6.564621 | 0.00000466 | Up |
| ENSG00000179388 | EGR3 | early_growth_response_3_[Source:HGNC_Symbol%3BAcc:HGNC:3240] | 6.616486 | 0.0000914 | Up |
| ENSG00000050730 | TNIP3 | TNFAIP3_interacting_protein_3_[Source:HGNC_Symbol%3BAcc:HGNC:19315] | 6.75401 | 0.014352 | Up |
| ENSG00000108700 | CCL8 | C-C_motif_chemokine_ligand_8_[Source:HGNC_Symbol%3BAcc:HGNC:10635] | 7.116189 | 0.00000623 | Up |
| ENSG00000163739 | CXCL1 | C-X-C_motif_chemokine_ligand_1_[Source:HGNC_Symbol%3BAcc:HGNC:4602] | 7.393818 | 3.77E-25 | Up |
| ENSG00000081041 | CXCL2 | C-X-C_motif_chemokine_ligand_2_[Source:HGNC_Symbol%3BAcc:HGNC:4603] | 7.393885 | 5.49E-21 | Up |
| ENSG00000115604 | IL18R1 | interleukin_18_receptor_1_[Source:HGNC_Symbol%3BAcc:HGNC:5988] | 7.484204 | 0.000435 | Up |
| ENSG00000123610 | TNFAIP6 | TNF_alpha_induced_protein_6_[Source:HGNC_Symbol%3BAcc:HGNC:11898] | 7.613272 | 1.33E-08 | Up |
| ENSG00000115008 | IL1A | interleukin_1_alpha_[Source:HGNC_Symbol%3BAcc:HGNC:5991] | 9.767759 | 1.36E-10 | Up |
| ENSG00000163734 | CXCL3 | C-X-C_motif_chemokine_ligand_3_[Source:HGNC_Symbol%3BAcc:HGNC:4604] | 10.33298 | 6.45E-19 | Up |

**Table S2. Continued.**

**Table S3. List of all DEGs of THP-1 cells co-cultured with ∆SPT OMVs compared to untreated THP-1 cells.**

| geneID | Name | Description | logFC | pValue | Change |
| --- | --- | --- | --- | --- | --- |
| ENSG00000141314 | RHBDL3 | rhomboid_like_3_[Source:HGNC_Symbol%3BAcc:HGNC:16502] | -5.7690032 | 0.0068685 | Down |
| ENSG00000175920 | DOK7 | docking_protein_7_[Source:HGNC_Symbol%3BAcc:HGNC:26594] | -5.5714324 | 0.00153145 | Down |
| ENSG00000160593 | JAML | junction_adhesion_molecule_like_[Source:HGNC_Symbol%3BAcc:HGNC:19084] | -5.1015831 | 0.02273902 | Down |
| ENSG00000173867 |  | novel_transcript | -4.9712032 | 0.02961816 | Down |
| ENSG00000121764 | HCRTR1 | hypocretin_receptor_1_[Source:HGNC_Symbol%3BAcc:HGNC:4848] | -4.9211591 | 0.01350998 | Down |
| ENSG00000066735 | KIF26A | kinesin_family_member_26A_[Source:HGNC_Symbol%3BAcc:HGNC:20226] | -4.9200856 | 0.03990189 | Down |
| ENSG00000113319 | RASGRF2 | Ras_protein_specific_guanine_nucleotide_releasing_factor_2_[Source:HGNC_Symbol%3BAcc:HGNC:9876] | -4.7828177 | 0.04080699 | Down |
| ENSG00000174804 | FZD4 | frizzled_class_receptor_4_[Source:HGNC_Symbol%3BAcc:HGNC:4042] | -4.7738607 | 0.01531723 | Down |
| ENSG00000187566 | NHLRC1 | NHL_repeat_containing_E3_ubiquitin_protein_ligase_1_[Source:HGNC_Symbol%3BAcc:HGNC:21576] | -4.7343024 | 0.0214509 | Down |
| ENSG00000111728 | ST8SIA1 | ST8_alpha-N-acetyl-neuraminide_alpha-2%2C8-sialyltransferase_1_[Source:HGNC_Symbol%3BAcc:HGNC:10869] | -4.6694691 | 0.04850159 | Down |
| ENSG00000173728 | C1orf100 | chromosome_1_open_reading_frame_100_[Source:HGNC_Symbol%3BAcc:HGNC:30435] | -4.6310667 | 0.04952247 | Down |
| ENSG00000111087 | GLI1 | GLI_family_zinc_finger_1_[Source:HGNC_Symbol%3BAcc:HGNC:4317] | -4.4879071 | 0.04066212 | Down |
| ENSG00000085465 | OVGP1 | oviductal_glycoprotein_1_[Source:HGNC_Symbol%3BAcc:HGNC:8524] | -4.4685455 | 0.04346672 | Down |
| ENSG00000249141 |  | novel_protein | -3.7149782 | 0.00921362 | Down |
| ENSG00000100027 | YPEL1 | yippee_like_1_[Source:HGNC_Symbol%3BAcc:HGNC:12845] | -3.4137491 | 0.03191816 | Down |
| ENSG00000136944 | LMX1B | LIM_homeobox_transcription_factor_1_beta_[Source:HGNC_Symbol%3BAcc:HGNC:6654] | -3.3226639 | 0.01729128 | Down |
| ENSG00000066405 | CLDN18 | claudin_18_[Source:HGNC_Symbol%3BAcc:HGNC:2039] | -2.7074423 | 0.00855979 | Down |
| ENSG00000123405 | NFE2 | nuclear_factor%2C_erythroid_2_[Source:HGNC_Symbol%3BAcc:HGNC:7780] | -2.7006371 | 0.00218188 | Down |
| ENSG00000269026 |  | novel_protein | -2.5767644 | 0.01379987 | Down |
| ENSG00000187634 | SAMD11 | sterile_alpha_motif_domain_containing_11_[Source:HGNC_Symbol%3BAcc:HGNC:28706] | -2.5031369 | 0.01378053 | Down |
| ENSG00000135298 | ADGRB3 | adhesion_G_protein-coupled_receptor_B3_[Source:HGNC_Symbol%3BAcc:HGNC:945] | -2.4268579 | 0.00376244 | Down |
| ENSG00000186806 | VSIG10L | V-set_and_immunoglobulin_domain_containing_10_like_[Source:HGNC_Symbol%3BAcc:HGNC:27111] | -2.426052 | 0.01076659 | Down |
| ENSG00000181004 | BBS12 | Bardet-Biedl_syndrome_12_[Source:HGNC_Symbol%3BAcc:HGNC:26648] | -2.2778033 | 0.04299677 | Down |
| ENSG00000135312 | HTR1B | 5-hydroxytryptamine_receptor_1B_[Source:HGNC_Symbol%3BAcc:HGNC:5287] | -2.2734671 | 0.04927468 | Down |

| ENSG00000168280 | KIF5C | kinesin_family_member_5C_[Source:HGNC_Symbol%3BAcc:HGNC:6325] | -2.0524687 | 0.03256215 | Down |
| --- | --- | --- | --- | --- | --- |
| ENSG00000116885 | OSCP1 | organic_solute_carrier_partner_1_[Source:HGNC_Symbol%3BAcc:HGNC:29971] | -2.0208581 | 0.04979967 | Down |
| ENSG00000198597 | ZNF536 | zinc_finger_protein_536_[Source:HGNC_Symbol%3BAcc:HGNC:29025] | -1.7296125 | 0.01922647 | Down |
| ENSG00000158874 | APOA2 | apolipoprotein_A2_[Source:HGNC_Symbol%3BAcc:HGNC:601] | -1.7231159 | 0.02152993 | Down |
| ENSG00000149571 | KIRREL3 | kirre_like_nephrin_family_adhesion_molecule_3_[Source:HGNC_Symbol%3BAcc:HGNC:23204] | -1.7207425 | 0.01764093 | Down |
| ENSG00000069764 | PLA2G10 | phospholipase_A2_group_X_[Source:HGNC_Symbol%3BAcc:HGNC:9029] | -1.7000156 | 0.04743065 | Down |
| ENSG00000164707 | SLC13A4 | solute_carrier_family_13_member_4_[Source:HGNC_Symbol%3BAcc:HGNC:15827] | -1.6861639 | 0.01781507 | Down |
| ENSG00000004799 | PDK4 | pyruvate_dehydrogenase_kinase_4_[Source:HGNC_Symbol%3BAcc:HGNC:8812] | -1.6772168 | 0.01277377 | Down |
| ENSG00000114737 | CISH | cytokine_inducible_SH2_containing_protein_[Source:HGNC_Symbol%3BAcc:HGNC:1984] | -1.6612068 | 0.02041892 | Down |
| ENSG00000166578 | IQCD | IQ_motif_containing_D_[Source:HGNC_Symbol%3BAcc:HGNC:25168] | -1.5818855 | 0.04374277 | Down |
| ENSG00000169740 | ZNF32 | zinc_finger_protein_32_[Source:HGNC_Symbol%3BAcc:HGNC:13095] | -1.5199686 | 0.0355274 | Down |
| ENSG00000185614 | INKA1 | inka_box_actin_regulator_1_[Source:HGNC_Symbol%3BAcc:HGNC:32480] | -1.4741095 | 0.01197733 | Down |
| ENSG00000124713 | GNMT | glycine_N-methyltransferase_[Source:HGNC_Symbol%3BAcc:HGNC:4415] | -1.437498 | 0.04885391 | Down |
| ENSG00000187187 | ZNF546 | zinc_finger_protein_546_[Source:HGNC_Symbol%3BAcc:HGNC:28671] | -1.4249858 | 0.0337665 | Down |
| ENSG00000168061 | SAC3D1 | SAC3_domain_containing_1_[Source:HGNC_Symbol%3BAcc:HGNC:30179] | -1.4134597 | 0.0397092 | Down |
| ENSG00000177191 | B3GNT8 | UDP-GlcNAc:betaGal_beta-1%2C3-N-acetylglucosaminyltransferase_8_[Source:HGNC_Symbol%3BAcc:HGNC:24139] | -1.4113007 | 0.0206497 | Down |
| ENSG00000175538 | KCNE3 | potassium_voltage-gated_channel_subfamily_E_regulatory_subunit_3_[Source:HGNC_Symbol%3BAcc:HGNC:6243] | -1.4043036 | 0.01117164 | Down |
| ENSG00000106366 | SERPINE1 | serpin_family_E_member_1_[Source:HGNC_Symbol%3BAcc:HGNC:8583] | -1.3431806 | 0.0225671 | Down |
| ENSG00000125735 | TNFSF14 | TNF_superfamily_member_14_[Source:HGNC_Symbol%3BAcc:HGNC:11930] | -1.3103294 | 0.01402984 | Down |
| ENSG00000168758 | SEMA4C | semaphorin_4C_[Source:HGNC_Symbol%3BAcc:HGNC:10731] | -1.3000963 | 0.01694612 | Down |
| ENSG00000104903 | LYL1 | LYL1_basic_helix-loop-helix_family_member_[Source:HGNC_Symbol%3BAcc:HGNC:6734] | -1.2168728 | 0.01387643 | Down |
| ENSG00000165424 | ZCCHC24 | zinc_finger_CCHC-type_containing_24_[Source:HGNC_Symbol%3BAcc:HGNC:26911] | -1.1586683 | 0.03778194 | Down |
| ENSG00000165752 | STK32C | serine/threonine_kinase_32C_[Source:HGNC_Symbol%3BAcc:HGNC:21332] | -1.1300754 | 0.04774298 | Down |
| ENSG00000160999 | SH2B2 | SH2B_adaptor_protein_2_[Source:HGNC_Symbol%3BAcc:HGNC:17381] | -1.0788763 | 0.02872983 | Down |
| ENSG00000184916 | JAG2 | jagged_canonical_Notch_ligand_2_[Source:HGNC_Symbol%3BAcc:HGNC:6189] | -1.0659596 | 0.04697468 | Down |
| ENSG00000143772 | ITPKB | inositol-trisphosphate_3-kinase_B_[Source:HGNC_Symbol%3BAcc:HGNC:6179] | -1.0449526 | 0.03169955 | Down |

**Table S3. Continued.**

**Table S3. Continued.**

| ENSG00000158882 | TOMM40L | translocase_of_outer_mitochondrial_membrane_40_like_[Source:HGNC_Symbol%3BAcc:HGNC:25756] | -1.034035 | 0.04751278 | Down |
| --- | --- | --- | --- | --- | --- |
| ENSG00000165030 | NFIL3 | nuclear_factor%2C_interleukin_3_regulated_[Source:HGNC_Symbol%3BAcc:HGNC:7787] | 1.0158866 | 0.04796339 | Up |
| ENSG00000104093 | DMXL2 | Dmx_like_2_[Source:HGNC_Symbol%3BAcc:HGNC:2938] | 1.01595618 | 0.04491843 | Up |
| ENSG00000157557 | ETS2 | ETS_proto-oncogene_2%2C_transcription_factor_[Source:HGNC_Symbol%3BAcc:HGNC:3489] | 1.0528237 | 0.01704842 | Up |
| ENSG00000104825 | NFKBIB | NFKB_inhibitor_beta_[Source:HGNC_Symbol%3BAcc:HGNC:7798] | 1.05517713 | 0.0429468 | Up |
| ENSG00000155629 | PIK3AP1 | phosphoinositide-3-kinase_adaptor_protein_1_[Source:HGNC_Symbol%3BAcc:HGNC:30034] | 1.07144283 | 0.01465217 | Up |
| ENSG00000116044 | NFE2L2 | nuclear_factor%2C_erythroid_2_like_2_[Source:HGNC_Symbol%3BAcc:HGNC:7782] | 1.09839877 | 0.02613796 | Up |
| ENSG00000178860 | MSC | musculin_[Source:HGNC_Symbol%3BAcc:HGNC:7321] | 1.1103938 | 0.01939093 | Up |
| ENSG00000100906 | NFKBIA | NFKB_inhibitor_alpha_[Source:HGNC_Symbol%3BAcc:HGNC:7797] | 1.12113837 | 0.0117014 | Up |
| ENSG00000159388 | BTG2 | BTG_anti-proliferation_factor_2_[Source:HGNC_Symbol%3BAcc:HGNC:1131] | 1.14456931 | 0.02511868 | Up |
| ENSG00000117594 | HSD11B1 | hydroxysteroid_11-beta_dehydrogenase_1_[Source:HGNC_Symbol%3BAcc:HGNC:5208] | 1.15603398 | 0.04950148 | Up |
| ENSG00000087074 | PPP1R15A | protein_phosphatase_1_regulatory_subunit_15A_[Source:HGNC_Symbol%3BAcc:HGNC:14375] | 1.15619007 | 0.01411624 | Up |
| ENSG00000184937 | WT1 | WT1_transcription_factor_[Source:HGNC_Symbol%3BAcc:HGNC:12796] | 1.16323984 | 0.04971565 | Up |
| ENSG00000160325 | CACFD1 | calcium_channel_flower_domain_containing_1_[Source:HGNC_Symbol%3BAcc:HGNC:1365] | 1.16476007 | 0.0291299 | Up |
| ENSG00000023608 | SNAPC1 | small_nuclear_RNA_activating_complex_polypeptide_1_[Source:HGNC_Symbol%3BAcc:HGNC:11134] | 1.16733794 | 0.04771952 | Up |
| ENSG00000165246 | NLGN4Y | neuroligin_4_Y-linked_[Source:HGNC_Symbol%3BAcc:HGNC:15529] | 1.17863768 | 0.0369383 | Up |
| ENSG00000213853 | EMP2 | epithelial_membrane_protein_2_[Source:HGNC_Symbol%3BAcc:HGNC:3334] | 1.2207855 | 0.02645358 | Up |
| ENSG00000184221 | OLIG1 | oligodendrocyte_transcription_factor_1_[Source:HGNC_Symbol%3BAcc:HGNC:16983] | 1.22108723 | 0.04830129 | Up |
| ENSG00000145901 | TNIP1 | TNFAIP3_interacting_protein_1_[Source:HGNC_Symbol%3BAcc:HGNC:16903] | 1.23722826 | 0.0134988 | Up |
| ENSG00000112297 | CRYBG1 | crystallin_beta-gamma_domain_containing_1_[Source:HGNC_Symbol%3BAcc:HGNC:356] | 1.24951062 | 0.0266464 | Up |
| ENSG00000171320 | ESCO2 | establishment_of_sister_chromatid_cohesion_N-acetyltransferase_2_[Source:HGNC_Symbol%3BAcc:HGNC:27230] | 1.26349035 | 0.02383905 | Up |
| ENSG00000104856 | RELB | RELB_proto-oncogene%2C_NF-kB_subunit_[Source:HGNC_Symbol%3BAcc:HGNC:9956] | 1.28005331 | 0.01057175 | Up |
| ENSG00000070961 | ATP2B1 | ATPase_plasma_membrane_Ca2+_transporting_1_[Source:HGNC_Symbol%3BAcc:HGNC:814] | 1.29315081 | 0.0185159 | Up |
| ENSG00000196664 | TLR7 | toll_like_receptor_7_[Source:HGNC_Symbol%3BAcc:HGNC:15631] | 1.30203688 | 0.0376212 | Up |
| ENSG00000113369 | ARRDC3 | arrestin_domain_containing_3_[Source:HGNC_Symbol%3BAcc:HGNC:29263] | 1.32902177 | 0.01273654 | Up |

| ENSG00000184588 | PDE4B | phosphodiesterase_4B_[Source:HGNC_Symbol%3BAcc:HGNC:8781] | 1.33583747 | 0.0408939 | Up |
| --- | --- | --- | --- | --- | --- |
| ENSG00000003402 | CFLAR | CASP8_and_FADD_like_apoptosis_regulator_[Source:HGNC_Symbol%3BAcc:HGNC:1876] | 1.34415207 | 0.00739326 | Up |
| ENSG00000152484 | USP12 | ubiquitin_specific_peptidase_12_[Source:HGNC_Symbol%3BAcc:HGNC:20485] | 1.34575228 | 0.00605153 | Up |
| ENSG00000155324 | GRAMD2B | GRAM_domain_containing_2B_[Source:HGNC_Symbol%3BAcc:HGNC:24911] | 1.35106439 | 0.04493428 | Up |
| ENSG00000165891 | E2F7 | E2F_transcription_factor_7_[Source:HGNC_Symbol%3BAcc:HGNC:23820] | 1.35938619 | 0.00873598 | Up |
| ENSG00000116285 | ERRFI1 | ERBB_receptor_feedback_inhibitor_1_[Source:HGNC_Symbol%3BAcc:HGNC:18185] | 1.36856265 | 0.03870124 | Up |
| ENSG00000102096 | PIM2 | Pim-2_proto-oncogene%2C_serine/threonine_kinase_[Source:HGNC_Symbol%3BAcc:HGNC:8987] | 1.36985992 | 0.0105204 | Up |
| ENSG00000149557 | FEZ1 | fasciculation_and_elongation_protein_zeta_1_[Source:HGNC_Symbol%3BAcc:HGNC:3659] | 1.38985472 | 0.01524786 | Up |
| ENSG00000124145 | SDC4 | syndecan_4_[Source:HGNC_Symbol%3BAcc:HGNC:10661] | 1.39237324 | 0.00328392 | Up |
| ENSG00000118263 | KLF7 | Kruppel_like_factor_7_[Source:HGNC_Symbol%3BAcc:HGNC:6350] | 1.39482707 | 0.00487731 | Up |
| ENSG00000121966 | CXCR4 | C-X-C_motif_chemokine_receptor_4_[Source:HGNC_Symbol%3BAcc:HGNC:2561] | 1.39613174 | 0.03869258 | Up |
| ENSG00000101017 | CD40 | CD40_molecule_[Source:HGNC_Symbol%3BAcc:HGNC:11919] | 1.39765013 | 0.0068545 | Up |
| ENSG00000185215 | TNFAIP2 | TNF_alpha_induced_protein_2_[Source:HGNC_Symbol%3BAcc:HGNC:11895] | 1.42135314 | 0.00712964 | Up |
| ENSG00000154640 | BTG3 | BTG_anti-proliferation_factor_3_[Source:HGNC_Symbol%3BAcc:HGNC:1132] | 1.4251136 | 0.01060417 | Up |
| ENSG00000211445 | GPX3 | glutathione_peroxidase_3_[Source:HGNC_Symbol%3BAcc:HGNC:4555] | 1.43282311 | 0.03738273 | Up |
| ENSG00000177045 | SIX5 | SIX_homeobox_5_[Source:HGNC_Symbol%3BAcc:HGNC:10891] | 1.46090847 | 0.02328186 | Up |
| ENSG00000116667 | C1orf21 | chromosome_1_open_reading_frame_21_[Source:HGNC_Symbol%3BAcc:HGNC:15494] | 1.46601693 | 0.03861416 | Up |
| ENSG00000169621 | APLF | aprataxin_and_PNKP_like_factor_[Source:HGNC_Symbol%3BAcc:HGNC:28724] | 1.51473402 | 0.04020329 | Up |
| ENSG00000135333 | EPHA7 | EPH_receptor_A7_[Source:HGNC_Symbol%3BAcc:HGNC:3390] | 1.51613502 | 0.0099857 | Up |
| ENSG00000126561 | STAT5A | signal_transducer_and_activator_of_transcription_5A_[Source:HGNC_Symbol%3BAcc:HGNC:11366] | 1.52581405 | 0.00976973 | Up |
| ENSG00000089351 | GRAMD1A | GRAM_domain_containing_1A_[Source:HGNC_Symbol%3BAcc:HGNC:29305] | 1.56504555 | 0.00240352 | Up |
| ENSG00000134571 | MYBPC3 | myosin_binding_protein_C3_[Source:HGNC_Symbol%3BAcc:HGNC:7551] | 1.57682163 | 0.03264185 | Up |
| ENSG00000145632 | PLK2 | polo_like_kinase_2_[Source:HGNC_Symbol%3BAcc:HGNC:19699] | 1.58742519 | 0.02885011 | Up |
| ENSG00000121989 | ACVR2A | activin_A_receptor_type_2A_[Source:HGNC_Symbol%3BAcc:HGNC:173] | 1.59514849 | 0.00836453 | Up |
| ENSG00000164236 | ANKRD33B | ankyrin_repeat_domain_33B_[Source:HGNC_Symbol%3BAcc:HGNC:35240] | 1.61181161 | 0.00988372 | Up |
| ENSG00000134138 | MEIS2 | Meis_homeobox_2_[Source:HGNC_Symbol%3BAcc:HGNC:7001] | 1.62350055 | 0.04104083 | Up |

**Table S3. Continued.**

**Table S3. Continued.**

| ENSG00000198355 | PIM3 | Pim-3_proto-oncogene%2C_serine/threonine_kinase_[Source:HGNC_Symbol%3BAcc:HGNC:19310] | 1.62358405 | 0.00140889 | Up |
| --- | --- | --- | --- | --- | --- |
| ENSG00000113504 | SLC12A7 | solute_carrier_family_12_member_7_[Source:HGNC_Symbol%3BAcc:HGNC:10915] | 1.62927686 | 0.00424679 | Up |
| ENSG00000152229 | PSTPIP2 | proline-serine-threonine_phosphatase_interacting_protein_2_[Source:HGNC_Symbol%3BAcc:HGNC:9581] | 1.64201108 | 0.02564981 | Up |
| ENSG00000110844 | PRPF40B | pre-mRNA_processing_factor_40_homolog_B_[Source:HGNC_Symbol%3BAcc:HGNC:25031] | 1.6459718 | 0.0228409 | Up |
| ENSG00000038427 | VCAN | versican_[Source:HGNC_Symbol%3BAcc:HGNC:2464] | 1.6584156 | 0.01262518 | Up |
| ENSG00000160326 | SLC2A6 | solute_carrier_family_2_member_6_[Source:HGNC_Symbol%3BAcc:HGNC:11011] | 1.67495448 | 0.00020191 | Up |
| ENSG00000165312 | OTUD1 | OTU_deubiquitinase_1_[Source:HGNC_Symbol%3BAcc:HGNC:27346] | 1.68247836 | 0.01289141 | Up |
| ENSG00000143067 | ZNF697 | zinc_finger_protein_697_[Source:HGNC_Symbol%3BAcc:HGNC:32034] | 1.68604583 | 0.00179422 | Up |
| ENSG00000112149 | CD83 | CD83_molecule_[Source:HGNC_Symbol%3BAcc:HGNC:1703] | 1.69193992 | 0.00156056 | Up |
| ENSG00000168685 | IL7R | interleukin_7_receptor_[Source:HGNC_Symbol%3BAcc:HGNC:6024] | 1.69370517 | 0.0425371 | Up |
| ENSG00000113916 | BCL6 | BCL6_transcription_repressor_[Source:HGNC_Symbol%3BAcc:HGNC:1001] | 1.7023287 | 0.00211268 | Up |
| ENSG00000164418 | GRIK2 | glutamate_ionotropic_receptor_kainate_type_subunit_2_[Source:HGNC_Symbol%3BAcc:HGNC:4580] | 1.71510707 | 0.03276278 | Up |
| ENSG00000188992 | LIPI | lipase_I_[Source:HGNC_Symbol%3BAcc:HGNC:18821] | 1.72173453 | 0.02744714 | Up |
| ENSG00000077150 | NFKB2 | nuclear_factor_kappa_B_subunit_2_[Source:HGNC_Symbol%3BAcc:HGNC:7795] | 1.7256322 | 0.00143634 | Up |
| ENSG00000111912 | NCOA7 | nuclear_receptor_coactivator_7_[Source:HGNC_Symbol%3BAcc:HGNC:21081] | 1.72793377 | 0.00631686 | Up |
| ENSG00000143344 | RGL1 | ral_guanine_nucleotide_dissociation_stimulator_like_1_[Source:HGNC_Symbol%3BAcc:HGNC:30281] | 1.7387364 | 0.02023713 | Up |
| ENSG00000168421 | RHOH | ras_homolog_family_member_H_[Source:HGNC_Symbol%3BAcc:HGNC:686] | 1.7491977 | 0.01028201 | Up |
| ENSG00000266094 | RASSF5 | Ras_association_domain_family_member_5_[Source:HGNC_Symbol%3BAcc:HGNC:17609] | 1.75427996 | 0.00579274 | Up |
| ENSG00000099992 | TBC1D10A | TBC1_domain_family_member_10A_[Source:HGNC_Symbol%3BAcc:HGNC:23609] | 1.76102179 | 0.03051582 | Up |
| ENSG00000169413 | RNASE6 | ribonuclease_A_family_member_k6_[Source:HGNC_Symbol%3BAcc:HGNC:10048] | 1.77732939 | 0.04352023 | Up |
| ENSG00000178878 | APOLD1 | apolipoprotein_L_domain_containing_1_[Source:HGNC_Symbol%3BAcc:HGNC:25268] | 1.78107961 | 0.03734576 | Up |
| ENSG00000095951 | HIVEP1 | HIVEP_zinc_finger_1_[Source:HGNC_Symbol%3BAcc:HGNC:4920] | 1.78858835 | 0.00594302 | Up |
| ENSG00000182168 | UNC5C | unc-5_netrin_receptor_C_[Source:HGNC_Symbol%3BAcc:HGNC:12569] | 1.80738492 | 0.02088252 | Up |
| ENSG00000090339 | ICAM1 | intercellular_adhesion_molecule_1_[Source:HGNC_Symbol%3BAcc:HGNC:5344] | 1.81273365 | 0.00043005 | Up |
| ENSG00000010818 | HIVEP2 | HIVEP_zinc_finger_2_[Source:HGNC_Symbol%3BAcc:HGNC:4921] | 1.82967776 | 0.02584575 | Up |

**Table S3. Continued.**

| ENSG00000175471 | MCTP1 | multiple_C2_and_transmembrane_domain_containing_1_[Source:HGNC_Symbol%3BAcc:HGNC:26183] | 1.83138909 | 0.01795141 | Up |
| --- | --- | --- | --- | --- | --- |
| ENSG00000186088 | GSAP | gamma-secretase_activating_protein_[Source:HGNC_Symbol%3BAcc:HGNC:28042] | 1.84091864 | 0.00897295 | Up |
| ENSG00000185022 | MAFF | MAF_bZIP_transcription_factor_F_[Source:HGNC_Symbol%3BAcc:HGNC:6780] | 1.84705775 | 0.00982296 | Up |
| ENSG00000125430 | HS3ST3B1 | heparan_sulfate-glucosamine_3-sulfotransferase_3B1_[Source:HGNC_Symbol%3BAcc:HGNC:5198] | 1.85247 | 0.0043283 | Up |
| ENSG00000135604 | STX11 | syntaxin_11_[Source:HGNC_Symbol%3BAcc:HGNC:11429] | 1.89023861 | 0.00256842 | Up |
| ENSG00000173517 | PEAK1 | pseudopodium_enriched_atypical_kinase_1_[Source:HGNC_Symbol%3BAcc:HGNC:29431] | 1.89351456 | 0.01024461 | Up |
| ENSG00000162924 | REL | REL_proto-oncogene%2C_NF-kB_subunit_[Source:HGNC_Symbol%3BAcc:HGNC:9954] | 1.89698443 | 0.00131532 | Up |
| ENSG00000136048 | DRAM1 | DNA_damage_regulated_autophagy_modulator_1_[Source:HGNC_Symbol%3BAcc:HGNC:25645] | 1.92007914 | 0.00106325 | Up |
| ENSG00000188396 | DYNLT4 | dynein_light_chain_Tctex-type_4_[Source:HGNC_Symbol%3BAcc:HGNC:32315] | 1.95798582 | 0.02966069 | Up |
| ENSG00000042062 | RIPOR3 | RIPOR_family_member_3_[Source:HGNC_Symbol%3BAcc:HGNC:16168] | 1.98468548 | 0.03037647 | Up |
| ENSG00000104312 | RIPK2 | receptor_interacting_serine/threonine_kinase_2_[Source:HGNC_Symbol%3BAcc:HGNC:10020] | 1.99466716 | 0.00113048 | Up |
| ENSG00000123700 | KCNJ2 | potassium_inwardly_rectifying_channel_subfamily_J_member_2_[Source:HGNC_Symbol%3BAcc:HGNC:6263] | 1.99559388 | 0.00871297 | Up |
| ENSG00000150347 | ARID5B | AT-rich_interaction_domain_5B_[Source:HGNC_Symbol%3BAcc:HGNC:17362] | 1.99857904 | 0.01859599 | Up |
| ENSG00000107968 | MAP3K8 | mitogen-activated_protein_kinase_kinase_kinase_8_[Source:HGNC_Symbol%3BAcc:HGNC:6860] | 2.00346484 | 0.00020611 | Up |
| ENSG00000050344 | NFE2L3 | nuclear_factor%2C_erythroid_2_like_3_[Source:HGNC_Symbol%3BAcc:HGNC:7783] | 2.00857271 | 0.01549523 | Up |
| ENSG00000184545 | DUSP8 | dual_specificity_phosphatase_8_[Source:HGNC_Symbol%3BAcc:HGNC:3074] | 2.03759698 | 0.00158821 | Up |
| ENSG00000109320 | NFKB1 | nuclear_factor_kappa_B_subunit_1_[Source:HGNC_Symbol%3BAcc:HGNC:7794] | 2.04516298 | 0.00143159 | Up |
| ENSG00000162437 | RAVER2 | ribonucleoprotein%2C_PTB_binding_2_[Source:HGNC_Symbol%3BAcc:HGNC:25577] | 2.04985067 | 0.04522593 | Up |
| ENSG00000108700 | CCL8 | C-C_motif_chemokine_ligand_8_[Source:HGNC_Symbol%3BAcc:HGNC:10635] | 2.05701541 | 0.01819201 | Up |
| ENSG00000172348 | RCAN2 | regulator_of_calcineurin_2_[Source:HGNC_Symbol%3BAcc:HGNC:3041] | 2.08375583 | 0.03297021 | Up |
| ENSG00000134070 | IRAK2 | interleukin_1_receptor_associated_kinase_2_[Source:HGNC_Symbol%3BAcc:HGNC:6113] | 2.09518105 | 0.00074767 | Up |
| ENSG00000277632 | CCL3 | C-C_motif_chemokine_ligand_3_[Source:HGNC_Symbol%3BAcc:HGNC:10627] | 2.0978639 | 0.00073557 | Up |
| ENSG00000067798 | NAV3 | neuron_navigator_3_[Source:HGNC_Symbol%3BAcc:HGNC:15998] | 2.10145724 | 0.00439232 | Up |
| ENSG00000167034 | NKX3-1 | NK3_homeobox_1_[Source:HGNC_Symbol%3BAcc:HGNC:7838] | 2.10879303 | 0.02558435 | Up |
| ENSG00000145365 | TIFA | TRAF_interacting_protein_with_forkhead_associated_domain_[Source:HGNC_Symbol%3BAcc:HGNC:19075] | 2.12607779 | 0.00042146 | Up |
| ENSG00000132510 | KDM6B | lysine_demethylase_6B_[Source:HGNC_Symbol%3BAcc:HGNC:29012] | 2.1404072 | 0.00080778 | Up |

**Table S3. Continued.**

| ENSG00000115919 | KYNU | kynureninase_[Source:HGNC_Symbol%3BAcc:HGNC:6469] | 2.14154002 | 0.00204515 | Up |
| --- | --- | --- | --- | --- | --- |
| ENSG00000164330 | EBF1 | EBF_transcription_factor_1_[Source:HGNC_Symbol%3BAcc:HGNC:3126] | 2.14703678 | 0.00324324 | Up |
| ENSG00000124233 | SEMG1 | semenogelin_1_[Source:HGNC_Symbol%3BAcc:HGNC:10742] | 2.15594863 | 0.04640581 | Up |
| ENSG00000137203 | TFAP2A | transcription_factor_AP-2_alpha_[Source:HGNC_Symbol%3BAcc:HGNC:11742] | 2.19504982 | 0.00189723 | Up |
| ENSG00000128917 | DLL4 | delta_like_canonical_Notch_ligand_4_[Source:HGNC_Symbol%3BAcc:HGNC:2910] | 2.19717463 | 0.00089837 | Up |
| ENSG00000153234 | NR4A2 | nuclear_receptor_subfamily_4_group_A_member_2_[Source:HGNC_Symbol%3BAcc:HGNC:7981] | 2.19895685 | 0.00705593 | Up |
| ENSG00000055813 | CCDC85A | coiled-coil_domain_containing_85A_[Source:HGNC_Symbol%3BAcc:HGNC:29400] | 2.24400677 | 0.04052267 | Up |
| ENSG00000131979 | GCH1 | GTP_cyclohydrolase_1_[Source:HGNC_Symbol%3BAcc:HGNC:4193] | 2.25017271 | 0.00637199 | Up |
| ENSG00000187950 | OVCH1 | ovochymase_1_[Source:HGNC_Symbol%3BAcc:HGNC:23080] | 2.2531847 | 0.01403449 | Up |
| ENSG00000276085 | CCL3L1 | C-C_motif_chemokine_ligand_3_like_1_[Source:HGNC_Symbol%3BAcc:HGNC:10628] | 2.26722912 | 0.00083576 | Up |
| ENSG00000163874 | ZC3H12A | zinc_finger_CCCH-type_containing_12A_[Source:HGNC_Symbol%3BAcc:HGNC:26259] | 2.27749543 | 9.3201E-07 | Up |
| ENSG00000214113 | LYRM4 | LYR_motif_containing_4_[Source:HGNC_Symbol%3BAcc:HGNC:21365] | 2.28272864 | 0.00305737 | Up |
| ENSG00000172817 | CYP7B1 | cytochrome_P450_family_7_subfamily_B_member_1_[Source:HGNC_Symbol%3BAcc:HGNC:2652] | 2.32443673 | 0.00079454 | Up |
| ENSG00000112096 | SOD2 | superoxide_dismutase_2_[Source:HGNC_Symbol%3BAcc:HGNC:11180] | 2.34652605 | 1.6612E-05 | Up |
| ENSG00000205189 | ZBTB10 | zinc_finger_and_BTB_domain_containing_10_[Source:HGNC_Symbol%3BAcc:HGNC:30953] | 2.35035592 | 0.00081039 | Up |
| ENSG00000125845 | BMP2 | bone_morphogenetic_protein_2_[Source:HGNC_Symbol%3BAcc:HGNC:1069] | 2.37133322 | 0.00185378 | Up |
| ENSG00000184557 | SOCS3 | suppressor_of_cytokine_signaling_3_[Source:HGNC_Symbol%3BAcc:HGNC:19391] | 2.37985903 | 9.2449E-07 | Up |
| ENSG00000107242 | PIP5K1B | phosphatidylinositol-4-phosphate_5-kinase_type_1_beta_[Source:HGNC_Symbol%3BAcc:HGNC:8995] | 2.44731849 | 0.00363557 | Up |
| ENSG00000118503 | TNFAIP3 | TNF_alpha_induced_protein_3_[Source:HGNC_Symbol%3BAcc:HGNC:11896] | 2.44996786 | 2.7714E-05 | Up |
| ENSG00000162645 | GBP2 | guanylate_binding_protein_2_[Source:HGNC_Symbol%3BAcc:HGNC:4183] | 2.45157919 | 0.00021096 | Up |
| ENSG00000155011 | DKK2 | dickkopf_WNT_signaling_pathway_inhibitor_2_[Source:HGNC_Symbol%3BAcc:HGNC:2892] | 2.46021219 | 0.04545734 | Up |
| ENSG00000275302 | CCL4 | C-C_motif_chemokine_ligand_4_[Source:HGNC_Symbol%3BAcc:HGNC:10630] | 2.47595319 | 0.00721726 | Up |
| ENSG00000105246 | EBI3 | Epstein-Barr_virus_induced_3_[Source:HGNC_Symbol%3BAcc:HGNC:3129] | 2.48712934 | 0.00057191 | Up |
| ENSG00000146674 | IGFBP3 | insulin_like_growth_factor_binding_protein_3_[Source:HGNC_Symbol%3BAcc:HGNC:5472] | 2.56651077 | 0.000247 | Up |
| ENSG00000112394 | SLC16A10 | solute_carrier_family_16_member_10_[Source:HGNC_Symbol%3BAcc:HGNC:17027] | 2.57093802 | 0.00850407 | Up |
| ENSG00000224420 | ADM5 | adrenomedullin_5_(putative)_[Source:HGNC_Symbol%3BAcc:HGNC:27293] | 2.61693089 | 0.04499585 | Up |

**Table S3. Continued.**

| ENSG00000166016 | ABTB2 | ankyrin_repeat_and_BTB_domain_containing_2_[Source:HGNC_Symbol%3BAcc:HGNC:23842] | 2.62134316 | 6.7252E-05 | Up |
| --- | --- | --- | --- | --- | --- |
| ENSG00000125538 | IL1B | interleukin_1_beta_[Source:HGNC_Symbol%3BAcc:HGNC:5992] | 2.65096233 | 6.5321E-07 | Up |
| ENSG00000138670 | RASGEF1B | RasGEF_domain_family_member_1B_[Source:HGNC_Symbol%3BAcc:HGNC:24881] | 2.65666591 | 0.00125747 | Up |
| ENSG00000091129 | NRCAM | neuronal_cell_adhesion_molecule_[Source:HGNC_Symbol%3BAcc:HGNC:7994] | 2.69980613 | 0.03118196 | Up |
| ENSG00000164603 | BMT2 | base_methyltransferase_of_25S_rRNA_2_homolog_[Source:HGNC_Symbol%3BAcc:HGNC:26475] | 2.70209587 | 0.00123281 | Up |
| ENSG00000276070 | CCL4L2 | C-C_motif_chemokine_ligand_4_like_2_[Source:HGNC_Symbol%3BAcc:HGNC:24066] | 2.73270293 | 0.0058266 | Up |
| ENSG00000288658 |  | novel_protein%2C_ortholog_of_Gm2102_(M._musculus) | 2.73429302 | 0.00083613 | Up |
| ENSG00000148459 | PDSS1 | decaprenyl_diphosphate_synthase_subunit_1_[Source:HGNC_Symbol%3BAcc:HGNC:17759] | 2.74445714 | 0.000406 | Up |
| ENSG00000163508 | EOMES | eomesodermin_[Source:HGNC_Symbol%3BAcc:HGNC:3372] | 2.78093495 | 0.00105289 | Up |
| ENSG00000163082 | SGPP2 | sphingosine-1-phosphate_phosphatase_2_[Source:HGNC_Symbol%3BAcc:HGNC:19953] | 2.79081163 | 0.03793446 | Up |
| ENSG00000145779 | TNFAIP8 | TNF_alpha_induced_protein_8_[Source:HGNC_Symbol%3BAcc:HGNC:17260] | 2.79931283 | 3.1305E-06 | Up |
| ENSG00000158050 | DUSP2 | dual_specificity_phosphatase_2_[Source:HGNC_Symbol%3BAcc:HGNC:3068] | 2.84839327 | 6.744E-05 | Up |
| ENSG00000023445 | BIRC3 | baculoviral_IAP_repeat_containing_3_[Source:HGNC_Symbol%3BAcc:HGNC:591] | 2.93988953 | 8.2046E-05 | Up |
| ENSG00000164949 | GEM | GTP_binding_protein_overexpressed_in_skeletal_muscle_[Source:HGNC_Symbol%3BAcc:HGNC:4234] | 2.9451116 | 2.3913E-05 | Up |
| ENSG00000112246 | SIM1 | SIM_bHLH_transcription_factor_1_[Source:HGNC_Symbol%3BAcc:HGNC:10882] | 2.94662488 | 0.03797704 | Up |
| ENSG00000122877 | EGR2 | early_growth_response_2_[Source:HGNC_Symbol%3BAcc:HGNC:3239] | 2.94835603 | 0.00074614 | Up |
| ENSG00000138378 | STAT4 | signal_transducer_and_activator_of_transcription_4_[Source:HGNC_Symbol%3BAcc:HGNC:11365] | 3.01978033 | 0.00580387 | Up |
| ENSG00000169429 | CXCL8 | C-X-C_motif_chemokine_ligand_8_[Source:HGNC_Symbol%3BAcc:HGNC:6025] | 3.02560729 | 4.4738E-06 | Up |
| ENSG00000144802 | NFKBIZ | NFKB_inhibitor_zeta_[Source:HGNC_Symbol%3BAcc:HGNC:29805] | 3.05047681 | 5.3514E-06 | Up |
| ENSG00000174469 | CNTNAP2 | contactin_associated_protein_2_[Source:HGNC_Symbol%3BAcc:HGNC:13830] | 3.1453827 | 0.03897125 | Up |
| ENSG00000137393 | RNF144B | ring_finger_protein_144B_[Source:HGNC_Symbol%3BAcc:HGNC:21578] | 3.17390703 | 0.00298997 | Up |
| ENSG00000123358 | NR4A1 | nuclear_receptor_subfamily_4_group_A_member_1_[Source:HGNC_Symbol%3BAcc:HGNC:7980] | 3.1753055 | 0.01035118 | Up |
| ENSG00000164056 | SPRY1 | sprouty_RTK_signaling_antagonist_1_[Source:HGNC_Symbol%3BAcc:HGNC:11269] | 3.21778975 | 0.01414813 | Up |
| ENSG00000115604 | IL18R1 | interleukin_18_receptor_1_[Source:HGNC_Symbol%3BAcc:HGNC:5988] | 3.27375527 | 0.02337521 | Up |
| ENSG00000138771 | SHROOM3 | shroom_family_member_3_[Source:HGNC_Symbol%3BAcc:HGNC:30422] | 3.27806233 | 0.00261153 | Up |
| ENSG00000150630 | VEGFC | vascular_endothelial_growth_factor_C_[Source:HGNC_Symbol%3BAcc:HGNC:12682] | 3.28508789 | 0.004087 | Up |

**Table S3. Continued.**

| ENSG00000121594 | CD80 | CD80_molecule_[Source:HGNC_Symbol%3BAcc:HGNC:1700] | 3.31194275 | 0.00333791 | Up |
| --- | --- | --- | --- | --- | --- |
| ENSG00000137331 | IER3 | immediate_early_response_3_[Source:HGNC_Symbol%3BAcc:HGNC:5392] | 3.33830345 | 1.0134E-07 | Up |
| ENSG00000164181 | ELOVL7 | ELOVL_fatty_acid_elongase_7_[Source:HGNC_Symbol%3BAcc:HGNC:26292] | 3.34851382 | 0.0003261 | Up |
| ENSG00000174576 | NPAS4 | neuronal_PAS_domain_protein_4_[Source:HGNC_Symbol%3BAcc:HGNC:18983] | 3.40290315 | 0.03559666 | Up |
| ENSG00000125384 | PTGER2 | prostaglandin_E_receptor_2_[Source:HGNC_Symbol%3BAcc:HGNC:9594] | 3.44854707 | 2.5005E-05 | Up |
| ENSG00000073756 | PTGS2 | prostaglandin-endoperoxide_synthase_2_[Source:HGNC_Symbol%3BAcc:HGNC:9605] | 3.47950644 | 9.7013E-05 | Up |
| ENSG00000108984 | MAP2K6 | mitogen-activated_protein_kinase_kinase_6_[Source:HGNC_Symbol%3BAcc:HGNC:6846] | 3.50059948 | 0.0101938 | Up |
| ENSG00000134901 | POGLUT2 | protein_O-glucosyltransferase_2_[Source:HGNC_Symbol%3BAcc:HGNC:19350] | 3.59614682 | 0.01429106 | Up |
| ENSG00000110944 | IL23A | interleukin_23_subunit_alpha_[Source:HGNC_Symbol%3BAcc:HGNC:15488] | 3.60384004 | 0.00010698 | Up |
| ENSG00000180611 | MB21D2 | Mab-21_domain_containing_2_[Source:HGNC_Symbol%3BAcc:HGNC:30438] | 3.65393861 | 0.04737485 | Up |
| ENSG00000179388 | EGR3 | early_growth_response_3_[Source:HGNC_Symbol%3BAcc:HGNC:3240] | 3.7795595 | 0.00533704 | Up |
| ENSG00000106511 | MEOX2 | mesenchyme_homeobox_2_[Source:HGNC_Symbol%3BAcc:HGNC:7014] | 3.80606499 | 0.03463606 | Up |
| ENSG00000059915 | PSD | pleckstrin_and_Sec7_domain_containing_[Source:HGNC_Symbol%3BAcc:HGNC:9507] | 3.82232038 | 0.00077735 | Up |
| ENSG00000117090 | SLAMF1 | signaling_lymphocytic_activation_molecule_family_member_1_[Source:HGNC_Symbol%3BAcc:HGNC:10903] | 3.83326989 | 0.00116987 | Up |
| ENSG00000115009 | CCL20 | C-C_motif_chemokine_ligand_20_[Source:HGNC_Symbol%3BAcc:HGNC:10619] | 3.84173543 | 4.0818E-10 | Up |
| ENSG00000142871 | CCN1 | cellular_communication_network_factor_1_[Source:HGNC_Symbol%3BAcc:HGNC:2654] | 3.88545196 | 0.02561368 | Up |
| ENSG00000056558 | TRAF1 | TNF_receptor_associated_factor_1_[Source:HGNC_Symbol%3BAcc:HGNC:12031] | 3.99533619 | 1.3246E-09 | Up |
| ENSG00000214511 | HIGD1C | HIG1_hypoxia_inducible_domain_family_member_1C_[Source:HGNC_Symbol%3BAcc:HGNC:28044] | 4.09100061 | 0.0091667 | Up |
| ENSG00000065320 | NTN1 | netrin_1_[Source:HGNC_Symbol%3BAcc:HGNC:8029] | 4.09726676 | 0.01052151 | Up |
| ENSG00000115008 | IL1A | interleukin_1_alpha_[Source:HGNC_Symbol%3BAcc:HGNC:5991] | 4.11085628 | 2.7614E-05 | Up |
| ENSG00000163739 | CXCL1 | C-X-C_motif_chemokine_ligand_1_[Source:HGNC_Symbol%3BAcc:HGNC:4602] | 4.12986279 | 2.2972E-11 | Up |
| ENSG00000138685 | FGF2 | fibroblast_growth_factor_2_[Source:HGNC_Symbol%3BAcc:HGNC:3676] | 4.15774003 | 0.00107629 | Up |
| ENSG00000137033 | IL33 | interleukin_33_[Source:HGNC_Symbol%3BAcc:HGNC:16028] | 4.30972493 | 0.01543033 | Up |
| ENSG00000150938 | CRIM1 | cysteine_rich_transmembrane_BMP_regulator_1_[Source:HGNC_Symbol%3BAcc:HGNC:2359] | 4.33787693 | 1.6193E-06 | Up |
| ENSG00000279212 |  | TEC | 4.56440602 | 0.0225411 | Up |
| ENSG00000104826 | LHB | luteinizing_hormone_subunit_beta_[Source:HGNC_Symbol%3BAcc:HGNC:6584] | 4.56971745 | 0.03055649 | Up |
| ENSG00000179564 | LSMEM2 | leucine_rich_single-pass_membrane_protein_2_[Source:HGNC_Symbol%3BAcc:HGNC:26781] | 4.56981783 | 0.03070932 | Up |

**Table S3. Continued.**

| ENSG00000125820 | NKX2-2 | NK2_homeobox_2_[Source:HGNC_Symbol%3BAcc:HGNC:7835] | 4.58622756 | 0.04997864 | Up |
| --- | --- | --- | --- | --- | --- |
| ENSG00000188676 | IDO2 | indoleamine_2%2C3-dioxygenase_2_[Source:HGNC_Symbol%3BAcc:HGNC:27269] | 4.72884261 | 0.03758669 | Up |
| ENSG00000127954 | STEAP4 | STEAP4_metalloreductase_[Source:HGNC_Symbol%3BAcc:HGNC:21923] | 4.76247788 | 0.04115031 | Up |
| ENSG00000118523 | CCN2 | cellular_communication_network_factor_2_[Source:HGNC_Symbol%3BAcc:HGNC:2500] | 4.80659943 | 2.8435E-10 | Up |
| ENSG00000258461 |  | novel_transcript | 4.85218759 | 0.03388711 | Up |
| ENSG00000268696 | ZNF723 | zinc_finger_protein_723_[Source:HGNC_Symbol%3BAcc:HGNC:32286] | 4.85832436 | 0.03669209 | Up |
| ENSG00000165959 | CLMN | calmin_[Source:HGNC_Symbol%3BAcc:HGNC:19972] | 4.86146499 | 0.03263129 | Up |
| ENSG00000100311 | PDGFB | platelet_derived_growth_factor_subunit_B_[Source:HGNC_Symbol%3BAcc:HGNC:8800] | 4.878554 | 0.02933029 | Up |
| ENSG00000157168 | NRG1 | neuregulin_1_[Source:HGNC_Symbol%3BAcc:HGNC:7997] | 4.95440349 | 0.01064223 | Up |
| ENSG00000077274 | CAPN6 | calpain_6_[Source:HGNC_Symbol%3BAcc:HGNC:1483] | 4.97670286 | 0.02877129 | Up |
| ENSG00000081041 | CXCL2 | C-X-C_motif_chemokine_ligand_2_[Source:HGNC_Symbol%3BAcc:HGNC:4603] | 5.02923517 | 1.1452E-10 | Up |
| ENSG00000105711 | SCN1B | sodium_voltage-gated_channel_beta_subunit_1_[Source:HGNC_Symbol%3BAcc:HGNC:10586] | 5.06006595 | 0.00688772 | Up |
| ENSG00000092295 | TGM1 | transglutaminase_1_[Source:HGNC_Symbol%3BAcc:HGNC:11777] | 5.18068923 | 0.00528535 | Up |
| ENSG00000054598 | FOXC1 | forkhead_box_C1_[Source:HGNC_Symbol%3BAcc:HGNC:3800] | 5.27668891 | 0.00394698 | Up |
| ENSG00000123610 | TNFAIP6 | TNF_alpha_induced_protein_6_[Source:HGNC_Symbol%3BAcc:HGNC:11898] | 5.34524828 | 2.6842E-06 | Up |
| ENSG00000138650 | PCDH10 | protocadherin_10_[Source:HGNC_Symbol%3BAcc:HGNC:13404] | 5.46901141 | 0.01366038 | Up |
| ENSG00000280228 |  | TEC | 5.469473 | 0.00656594 | Up |
| ENSG00000122641 | INHBA | inhibin_subunit_beta_A_[Source:HGNC_Symbol%3BAcc:HGNC:6066] | 5.4978812 | 0.03104017 | Up |
| ENSG00000119508 | NR4A3 | nuclear_receptor_subfamily_4_group_A_member_3_[Source:HGNC_Symbol%3BAcc:HGNC:7982] | 5.52099079 | 4.2556E-06 | Up |
| ENSG00000182050 | MGAT4C | MGAT4_family_member_C_[Source:HGNC_Symbol%3BAcc:HGNC:30871] | 5.69880595 | 0.02275502 | Up |
| ENSG00000061918 | GUCY1B1 | guanylate_cyclase_1_soluble_subunit_beta_1_[Source:HGNC_Symbol%3BAcc:HGNC:4687] | 5.69881395 | 0.02277296 | Up |
| ENSG00000073282 | TP63 | tumor_protein_p63_[Source:HGNC_Symbol%3BAcc:HGNC:15979] | 5.78299977 | 0.00072617 | Up |
| ENSG00000163734 | CXCL3 | C-X-C_motif_chemokine_ligand_3_[Source:HGNC_Symbol%3BAcc:HGNC:4604] | 5.9705439 | 1.1055E-12 | Up |
| ENSG00000137265 | IRF4 | interferon_regulatory_factor_4_[Source:HGNC_Symbol%3BAcc:HGNC:6119] | 5.99280369 | 0.01050169 | Up |
| ENSG00000136244 | IL6 | interleukin_6_[Source:HGNC_Symbol%3BAcc:HGNC:6018] | 6.25300617 | 9.5777E-05 | Up |
| ENSG00000144810 | COL8A1 | collagen_type_VIII_alpha_1_chain_[Source:HGNC_Symbol%3BAcc:HGNC:2215] | 6.41941542 | 0.02068549 | Up |

**Table S3. Continued.**

| ENSG00000115607 | IL18RAP | interleukin_18_receptor_accessory_protein_[Source:HGNC_Symbol%3BAcc:HGNC:5989] | 6.52592738 | 0.01855781 | Up |
| --- | --- | --- | --- | --- | --- |
| ENSG00000138639 | ARHGAP24 | Rho_GTPase_activating_protein_24_[Source:HGNC_Symbol%3BAcc:HGNC:25361] | 6.71088622 | 0.00335117 | Up |
| ENSG00000078081 | LAMP3 | lysosomal_associated_membrane_protein_3_[Source:HGNC_Symbol%3BAcc:HGNC:14582] | 7.07341103 | 9.8873E-05 | Up |

**Table S4. Top ten downregulated and upregulated DEGs of THP-1 cells co-cultured with WT OMVs compared to untreated THP-1 cells.**

| geneID | Name | Description | logFC | pValue | Change |
| --- | --- | --- | --- | --- | --- |
| ENSG00000115008 | IL1A | interleukin_1_alpha | -5.69601 | 0.002856 | Down |
| ENSG00000205436 | EXOC3L4 | exocyst_complex_component_3_like_4 | -5.22846 | 0.009081 | Down |
| ENSG00000130997 | POLN | DNA_polymerase_nu | -5.15988 | 0.024423 | Down |
| ENSG00000205929 | C21orf62 | chromosome_21_open_reading_frame_62 | -5.12291 | 0.022675 | Down |
| ENSG00000108700 | CCL8 | C-C_motif_chemokine_ligand_8 | -5.09308 | 0.01191 | Down |
| ENSG00000213221 | DNLZ | DNL-type_zinc_finger | -5.07053 | 0.025612 | Down |
| ENSG00000176204 | LRRTM4 | leucine_rich_repeat_transmembrane_neuronal_4 | -4.89374 | 0.034877 | Down |
| ENSG00000276231 | PIK3R6 | phosphoinositide-3-kinase_regulatory_subunit_6 | -4.89024 | 0.013313 | Down |
| ENSG00000197191 | CYSRT1 | cysteine_rich_tail_1 | -4.78949 | 0.036945 | Down |
| ENSG00000175564 | UCP3 | uncoupling_protein_3 | -4.75536 | 0.022764 | Down |
| ENSG00000087510 | TFAP2C | transcription_factor_AP-2_gamma | 3.801389 | 0.030831 | Up |
| ENSG00000117228 | GBP1 | guanylate_binding_protein_1 | 4.203838 | 0.00366 | Up |
| ENSG00000288721 | 0 | novel_protein | 4.617138 | 0.04582 | Up |
| ENSG00000159409 | CELF3 | CUGBP_Elav-like_family_member_3 | 4.758008 | 0.041168 | Up |
| ENSG00000279164 | 0 | TEC | 4.797151 | 0.018686 | Up |
| ENSG00000105146 | AURKC | aurora_kinase_C | 4.834345 | 0.014301 | Up |
| ENSG00000214050 | FBXO16 | F-box_protein_16 | 4.91726 | 0.015959 | Up |
| ENSG00000145864 | GABRB2 | gamma-aminobutyric_acid_type_A_receptor_subunit_beta2 | 5.05532 | 0.02581 | Up |
| ENSG00000174469 | CNTNAP2 | contactin_associated_protein_2 | 5.289634 | 0.007067 | Up |
| ENSG00000165959 | CLMN | calmin_ | 5.503731 | 0.001593 | Up |

**Table S5. Top ten downregulated and upregulated DEGs of THP-1 cells co-cultured with ∆SPT OMVs compared to co-cultured with WT OMVs.**

| geneID | Name | Description | logFC | pValue | Change |
| --- | --- | --- | --- | --- | --- |
| ENSG00000256100 | 0 | novel_transcript | -5.1719581 | 0.01567 | Down |
| ENSG00000267127 | 0 | novel_protein | -5.1019224 | 0.027433 | Down |
| ENSG00000159409 | CELF3 | CUGBP_Elav-like_family_member_3 | -4.7466407 | 0.045952 | Down |
| ENSG00000126246 | IGFLR1 | IGF_like_family_receptor_1 | -4.7352669 | 0.041046 | Down |
| ENSG00000181690 | PLAG1 | PLAG1_zinc_finger | -4.6987399 | 0.02718 | Down |
| ENSG00000105289 | TJP3 | tight_junction_protein_3 | -4.5112325 | 0.042821 | Down |
| ENSG00000137968 | SLC44A5 | solute_carrier_family_44_member_5 | -4.1077652 | 0.010062 | Down |
| ENSG00000179240 | GVQW3 | GVQW_motif_containing_3 | -3.5430804 | 0.033631 | Down |
| ENSG00000135312 | HTR1B | 5-hydroxytryptamine_receptor_1B | -3.2646601 | 0.003905 | Down |
| ENSG00000124143 | ARHGAP40 | Rho_GTPase_activating_protein_40 | -2.9272214 | 0.016331 | Down |
| ENSG00000119508 | NR4A3 | nuclear_receptor_subfamily_4_group_A_member_3 | 6.56462101 | 4.66E-06 | Up |
| ENSG00000179388 | EGR3 | early_growth_response_3 | 6.61648613 | 9.14E-05 | Up |
| ENSG00000050730 | TNIP3 | TNFAIP3_interacting_protein_3 | 6.7540095 | 0.014352 | Up |
| ENSG00000108700 | CCL8 | C-C_motif_chemokine_ligand_8 | 7.1161885 | 6.23E-06 | Up |
| ENSG00000163739 | CXCL1 | C-X-C_motif_chemokine_ligand_1 | 7.39381783 | 3.77E-25 | Up |
| ENSG00000081041 | CXCL2 | C-X-C_motif_chemokine_ligand_2 | 7.39388538 | 5.49E-21 | Up |
| ENSG00000115604 | IL18R1 | interleukin_18_receptor_1 | 7.4842042 | 0.000435 | Up |
| ENSG00000123610 | TNFAIP6 | TNF_alpha_induced_protein_6 | 7.61327206 | 1.33E-08 | Up |
| ENSG00000115008 | IL1A | interleukin_1_alpha | 9.76775904 | 1.36E-10 | Up |
| ENSG00000163734 | CXCL3 | C-X-C_motif_chemokine_ligand_3 | 10.3329838 | 6.45E-19 | Up |

**Table S6. Top ten downregulated and upregulated DEGs of THP-1 cells co-cultured with ∆SPT OMVs compared to untreated THP-1 cells.**

| geneID | Name | Description | logFC | pValue | Change |
| --- | --- | --- | --- | --- | --- |
| ENSG00000141314 | RHBDL3 | rhomboid_like_3 | -5.769 | 0.006869 | Down |
| ENSG00000175920 | DOK7 | docking_protein_7 | -5.57143 | 0.001531 | Down |
| ENSG00000160593 | JAML | junction_adhesion_molecule_like | -5.10158 | 0.022739 | Down |
| ENSG00000173867 | 0 | novel_transcript | -4.9712 | 0.029618 | Down |
| ENSG00000121764 | HCRTR1 | hypocretin_receptor_1 | -4.92116 | 0.01351 | Down |
| ENSG00000066735 | KIF26A | kinesin_family_member_26A | -4.92009 | 0.039902 | Down |
| ENSG00000113319 | RASGRF2 | Ras_protein_specific_guanine_nucleotide_releasing_factor_2 | -4.78282 | 0.040807 | Down |
| ENSG00000174804 | FZD4 | frizzled_class_receptor_4 | -4.77386 | 0.015317 | Down |
| ENSG00000187566 | NHLRC1 | NHL_repeat_containing_E3_ubiquitin_protein_ligase_1 | -4.7343 | 0.021451 | Down |
| ENSG00000111728 | ST8SIA1 | ST8_alpha-N-acetyl-neuraminide_alpha-2%2C8-sialyltransferase_1 | -4.66947 | 0.048502 | Down |
| ENSG00000182050 | MGAT4C | MGAT4_family_member_C | 5.698806 | 0.022755 | Up |
| ENSG00000061918 | GUCY1B1 | guanylate_cyclase_1_soluble_subunit_beta_1 | 5.698814 | 0.022773 | Up |
| ENSG00000073282 | TP63 | tumor_protein_p63 | 5.783 | 0.000726 | Up |
| ENSG00000163734 | CXCL3 | C-X-C_motif_chemokine_ligand_3 | 5.970544 | 1.11E-12 | Up |
| ENSG00000137265 | IRF4 | interferon_regulatory_factor_4 | 5.992804 | 0.010502 | Up |
| ENSG00000136244 | IL6 | interleukin_6 | 6.253006 | 9.58E-05 | Up |
| ENSG00000144810 | COL8A1 | collagen_type_VIII_alpha_1_chain | 6.419415 | 0.020685 | Up |
| ENSG00000115607 | IL18RAP | interleukin_18_receptor_accessory_protein | 6.525927 | 0.018558 | Up |
| ENSG00000138639 | ARHGAP24 | Rho_GTPase_activating_protein_24 | 6.710886 | 0.003351 | Up |
| ENSG00000078081 | LAMP3 | lysosomal_associated_membrane_protein_3 | 7.073411 | 9.89E-05 | Up |

**Table S7. List of DEGs identified in the top enriched KEGG pathways in figure 8 (WT-CTRL).**

| ID | Description | NES | pvalue | Core_enrichment | Count | GeneRatio | Direction |
| --- | --- | --- | --- | --- | --- | --- | --- |
| hsa04668 | TNF signaling pathway | -1.823307201 | 0.000334 | CCL20/BCL3/NFKBIA/LTA/CCL2/PTGS2/VEGFC/IL1B/  TNF/BIRC3/CXCL2/TNFAIP3/VEGFD/FAS/CXCL1/IL18R1/CXCL3 | 17 | 0.16831683 | Suppressed |
| hsa04657 | IL-17 signaling pathway | -1.712824956 | 0.001195 | CCL20/NFKBIA/CCL2/PTGS2/IL17D/IL1B/TNF/CXCL2/  TNFAIP3/CXCL8/IL17C/CXCL1/CXCL3 | 13 | 0.203125 | Suppressed |
| hsa05134 | Legionellosis | -1.800636304 | 0.001733 | NFKB1/BNIP3/NFKBIA/IL1B/TNF/CXCL2/CXCL8/TLR5/CXCL1/CXCL3 | 10 | 0.20408163 | Suppressed |
| hsa00982 | Drug metabolism - cytochrome P450 | -1.670702912 | 0.006481 | CYP2D6/CYP3A5/ALDH3A1/AOX1 | 4 | 0.22222222 | Suppressed |
| hsa04062 | Chemokine signaling pathway | -1.598409073 | 0.001439 | HCK/SHC2/KRAS/RAP1A/ADCY6/CCL22/NCF1/SOS2/  NFKB1/CXCL12/VAV2/SHC3/ADCY4/BRAF/SRC/CCL20/NFKBIA/  GNG4/CCL2/CXCR1/CCL1/CCL3L1/CCL3/CXCL2/CXCL8/GRK7/CCL4/XCR1/CCL27/CCL4L2/CXCL1/CXCL3/PIK3R6/CCL8 | 34 | 0.22666667 | Suppressed |
| hsa04064 | NF-kappa B signaling pathway | -1.569525989 | 0.004035 | NFKB1/CARD10/CXCL12/PRKCQ/LTB/TRAF3/CD40/RELB/  NFKBIA/LTA/PTGS2/IL1B/TNF/BIRC3/CXCL2/TNFAIP3/CXCL8/  CCL4/CCL4L2/CXCL1/CXCL3 | 21 | 0.23333333 | Suppressed |
| hsa05323 | Rheumatoid arthritis | -1.589640271 | 0.005824 | CD86/CXCL12/HLA-DQB1/LTB/IL23A/CCL20/HLA-DRA/  CCL2/IL1B/TNF/CCL3L1/CCL3/CXCL2/CXCL8/HLA-DQA1/CXCL1/  CXCL3/IL1A | 18 | 0.28571429 | Suppressed |
| hsa05417 | Lipid and atherosclerosis | -1.592240341 | 0.00079 | TICAM1/TNFRSF10A/MIB1/RAP1B/PIK3CA/PIK3CB/GSK3B/  PLCB1/TRAF6/NFE2L2/XBP1/POU2F2/MAPK11/POU2F3/TNFRSF10B/  NOS3/ICAM1/BAD/TRAF2/MAPK8/IRAK4/KRAS/RAP1A/NCF1/LDLR/ABCA1/NFKB1/VLDLR/VAV2/ABCG1/TRAF3/HSPA2/CD40/NOX1/SRC/NFKBIA/TP53/CAMK2A/CCL2/APOA1/IL1B/TNF/DDIT3/CCL3L1/  CCL3/CXCL2/CXCL8/FAS/CXCL1/CXCL3 | 50 | 0.3030303 | Suppressed |
| hsa04060 | Cytokine-cytokine receptor interaction | -1.734561561 | 0.000317 | CCL22/EBI3/CRLF2/CD70/CXCL12/IL1RAP/IL33/LTB/INHBE  /CSF3R/CD40/TNFSF9/IL31RA/OSM/IL23A/CCL20/TSLP/LTA/  CNTF/GDF7/CCL2/IL17D/BMP4/IL1B/TNF/CXCR1/IFNAR2/CCL1/IL9R/IL13RA2/IL27/AMH/CCL3L1/CCL3/CXCL2/CXCL8/IL17C/NODAL/CCL4/XCR1/CCL27/FAS/CCL4L2/CXCL1/TNFRSF18/IL10RB/IL18R1/CXCL3/CCL8/IL1A | 50 | 0.32258065 | Suppressed |
| hsa04061 | Viral protein interaction with cytokine and cytokine receptor | -1.913750098 | 0.000344 | CCL20/LTA/CCL2/TNF/CXCR1/CCL1/CCL3L1/CCL3/CXCL2/CXCL8/  CCL4/XCR1/CCL27/CCL4L2/CXCL1/IL10RB/IL18R1/  CXCL3/CCL8 | 19 | 0.33333333 | Suppressed |
| hsa04933 | AGE-RAGE signaling pathway in diabetic complications | -1.579955066 | 0.003861 | BCL2/PLCB4/SMAD4/FN1/COL4A4/AGER/PRKCZ/PLCE1/PIK3CA/SMAD3/PIK3CB/PLCB1/VEGFA/PRKCE/MAPK11/NOS3/ICAM1/MAPK8/KRAS/NFKB1/SERPINE1/NOX1/CCL2/VEGFC/F3/IL1B/TNF/EGR1/CXCL8/  VEGFD/IL1A | 31 | 0.36046512 | Suppressed |
| hsa03030 | DNA replication | 1.715442658 | 0.006661 | PRIM1/RFC3/MCM4/MCM5/RFC4/POLD1/MCM2/MCM3/PCNA/POLE/MCM6/RPA1/MCM7/POLD3/LIG1/RFC1/RFC5 | 17 | 0.56666667 | Activated |

**Table S8. List of DEGs identified in the top enriched KEGG pathways in figure 9 (SPT-WT).**

| ID | Description | NES | pvalue | Core_enrichment | Count | GeneRatio | Direction |
| --- | --- | --- | --- | --- | --- | --- | --- |
| hsa00010 | Glycolysis / Gluconeogenesis | -1.629750526 | 0.011544462 | BPGM/GALM/ALDOA/ENO4/ALDOB/ALDH7A1 | 6 | 0.13953488 | Suppressed |
| hsa04623 | Cytosolic DNA-sensing pathway | 1.886107961 | 0.000277701 | IL6/CCL4L2/CCL4/IL33/IL1B/ZDHHC11/IFNB1/ZDHHC11B/NFKB1/NFKBIA | 10 | 0.15873016 | Activated |
| hsa05164 | Influenza A | 1.887670536 | 0.00012486 | IL1A/IL6/CXCL8/IL33/IL12B/IL1B/FAS/IFNB1/CPSF4L/TMPRSS11D/PRSS3/TNF/SOCS3/NFKB1/CCL2/ICAM1/NFKBIA/HLA-DQA1/TRAF3/TICAM1/NFKBIB/NXT2/HLA-DRA | 23 | 0.17557252 | Activated |
| hsa05167 | Kaposi sarcoma-associated herpesvirus infection | 1.943341473 | 0.000121065 | CXCL3/CXCL2/CXCL1/IL6/CXCL8/PTGS2/PDGFB/PIK3R6/FAS/IFNB1/CD200R1L/CLEC2B/FGF2/NFKB1/CSF2/MAP2K6/ICAM1/GNG4/NFKBIA/TP53/TRAF3/SRC/TCF7L2/TICAM1/ZFP36/PIK3R5/TCF7/  NFATC1/TRAF2/CREB1 | 30 | 0.19354839 | Activated |
| hsa04620 | Toll-like receptor signaling pathway | 2.040027919 | 0.000133779 | IL6/CCL4L2/CCL4/CXCL8/IL12B/IL1B/CCL3L1/CCL3/CXCL9/CD80/IFNB1/TNF/NFKB1/MAP2K6/MAP3K8/CD40/NFKBIA | 17 | 0.20987654 | Activated |
| hsa05134 | Legionellosis | 2.08698524 | 0.000143041 | CXCL3/CXCL2/CXCL1/IL6/CXCL8/IL12B/IL1B/TNF/NFKB1/  NFKBIA/NFKB2 | 11 | 0.22 | Activated |
| hsa04625 | C-type lectin receptor signaling pathway | 1.943236962 | 0.00013224 | EGR3/IL6/PTGS2/IL12B/IL23A/IL1B/EGR2/TNF/NFKB1/RELB/  NFKBIA/NFKB2/BCL3/SRC/PLK3/CCL22/CYLD/IL17D/NFATC1 | 19 | 0.22093023 | Activated |
| hsa00051 | Fructose and mannose metabolism | -1.611027587 | 0.016521983 | PFKFB2/GFUS/FCSK/PFKFB1/KHK/ALDOA/ALDOB | 7 | 0.25 | Suppressed |
| hsa04657 | IL-17 signaling pathway | 2.120870812 | 0.000136277 | CXCL3/CXCL2/CXCL1/IL6/CXCL8/PTGS2/TNFAIP3/CCL20/IL1B/  CXCL6/IL17C/TNF/NFKB1/CSF2/CCL2/NFKBIA/MUC5B/TRAF3 | 18 | 0.26470588 | Activated |
| hsa04064 | NF-kappa B signaling pathway | 1.986336014 | 0.000131303 | CXCL3/CXCL2/CXCL1/CCL4L2/CCL4/CXCL8/BIRC3/PTGS2/TNFAIP3/IL1B/TRAF1/VCAM1/TNF/NFKB1/ICAM1/RELB/LTA/CD40/  NFKBIA/NFKB2/CD40LG/TRAF3/CFLAR/CXCL12/PRKCQ/BLNK/  LTB/TICAM1 | 28 | 0.30434783 | Activated |
| hsa04668 | TNF signaling pathway | 2.286784153 | 0.0001287 | CXCL3/IL18R1/CXCL2/CXCL1/IL6/BIRC3/PTGS2/TNFAIP3/CCL20/VEGFC/IL1B/FAS/CXCL6/IFNB1/TRAF1/VCAM1/IL15/TNF/SOCS3/NFKB1/CSF2/MAP2K6/LIF/CCL2/MAP3K8/ICAM1/LTA/NFKBIA/  EDN1/BCL3/TRAF3/CFLAR/CSF1 | 33 | 0.30841121 | Activated |
| hsa05321 | Inflammatory bowel disease | 1.980566752 | 0.000147471 | IL1A/IL18R1/IL18RAP/IL6/STAT4/IL12B/IL23A/IL1B/TNF/TBX21/  NFKB1/IL23R/HLA-DQA1 | 13 | 0.30952381 | Activated |
| hsa05323 | Rheumatoid arthritis | 2.078896199 | 0.000136426 | CXCL3/IL1A/CXCL2/CXCL1/IL6/CXCL8/CCL20/IL23A/IL1B/CCL3L1/CCL3/CXCL6/CD80/IL15/TNF/CSF2/CCL2/ICAM1/HLA-DQA1/CXCL12/CSF1/LTB | 22 | 0.32835821 | Activated |
| hsa05144 | Malaria | 1.859163974 | 0.000152509 | IL6/CXCL8/IL1B/HGF/VCAM1/HBA2/TNF/CCL2/ICAM1/CD40/  CD40LG | 11 | 0.35483871 | Activated |
| hsa00053 | Ascorbate and aldarate metabolism | -1.610888328 | 0.022824536 | UGDH/ALDH3A2/MIOX/ALDH7A1 | 4 | 0.36363636 | Suppressed |
| hsa04060 | Cytokine-cytokine receptor interaction | 2.15012183 | 0.000119717 | CXCL3/IL1A/IL18R1/CXCL2/CXCL1/CCL8/IL18RAP/IL6/CCL4L2/CCL4/TNFRSF18/CXCL8/INHBA/IL33/CCL20/IL12B/IL23A/IL1B/CCL3L1/CCL3/CXCL9/FAS/CXCL6/IFNB1/CXCL13/INHBB/CCR10/IL15/TNFRSF19/TNFSF18/EBI3/IL17C/TNF/CSF2/LIF/AMH/CCL2/LTA/  CD40/CXCL14/IL34/TNFRSF11B/IL23R/IL27/BMP2/IL13RA2/  IL10RB/CD40LG/TNFSF9/ACVR2A/CNTF/CXCL12/CSF1/TSLP/LTB/  CXCR4/IL15RA/IL7R/CD70/TNFRSF9/RELL1/ACKR3/CCL22 | 63 | 0.37951807 | Activated |
| hsa05143 | African trypanosomiasis | 1.979117591 | 0.000160772 | IL6/IL12B/IDO2/IL1B/FAS/VCAM1/HBA2/TNF/ICAM1 | 9 | 0.42857143 | Activated |
| hsa04061 | Viral protein interaction with cytokine and cytokine receptor | 2.080376142 | 0.000139353 | CXCL3/IL18R1/CXCL2/CXCL1/CCL8/IL18RAP/IL6/CCL4L2/CCL4/  CXCL8/CCL20/CCL3L1/CCL3/CXCL9/CXCL6/CXCL13/CCR10/TNF/CCL2/LTA/CXCL14/IL34/IL10RB/CXCL12/CSF1/CXCR4/ACKR3/  CCL22 | 28 | 0.46666667 | Activated |

**Table S9. List of DEGs identified in the top enriched KEGG pathways in figure S7 (SPT-CTRL).**

| ID | Description | NES | pvalue | Core_enrichment | Count | GeneRatio | Direction |
| --- | --- | --- | --- | --- | --- | --- | --- |
| hsa04623 | Cytosolic DNA-sensing pathway | 1.790919814 | 0.000299 | IL6/IL33/ZDHHC11/IFNB1/CCL4L2/IL1B/CCL4/NFKB1/ZDHHC11B/NFKBIA/NFKBIB | 11 | 0.17 | Activated |
| hsa05164 | Influenza A | 1.802259992 | 0.000136 | IL6/IL12B/IL33/IL1A/IFNB1/IRF9/CPSF4L/PRSS3/CXCL8/IL1B/  SOCS3/NFKB1/TLR3/ICAM1/TLR7/TNF/NFKBIA/NFKBIB/CCL2/  TICAM1/FAS/TRAF3/IFIH1 | 23 | 0.18 | Activated |
| hsa05167 | Kaposi sarcoma-associated herpesvirus infection | 1.865933057 | 0.000133 | IL6/CXCL3/CXCL2/PDGFB/FGF2/CXCL1/IFNB1/CD200R1L/IRF9/  MAP2K6/PTGS2/CXCL8/CCR5/CSF2/NFKB1/TLR3/ICAM1/CLEC2B/GNG4/NFKBIA/LEF1/TICAM1/FAS/TRAF3/TP53/TCF7L2/E2F2/  PIK3R5 | 28 | 0.18 | Activated |
| hsa05134 | Legionellosis | 1.795166621 | 0.000306 | IL6/CXCL3/CXCL2/IL12B/CXCL1/CXCL8/IL1B/NFKB1/NFKB2/  TNF/NFKBIA | 11 | 0.22 | Activated |
| hsa05133 | Pertussis | 1.807631524 | 0.000302 | IL6/IL12B/CFL1/IL1A/CXCL6/IL23A/CXCL8/IL1B/NFKB1/IL10/  NOS2/TNF/TICAM1 | 13 | 0.22 | Activated |
| hsa00982 | Drug metabolism - cytochrome P450 | -1.624706203 | 0.015746 | CYP3A5/GSTO2/GSTM2/ALDH3A1 | 4 | 0.22 | Suppressed |
| hsa04625 | C-type lectin receptor signaling pathway | 1.806702979 | 0.000288 | IL6/IL12B/EGR3/IL23A/IRF9/PTGS2/EGR2/IL1B/NFKB1/IL10/  NFKB2/RELB/TNF/NFKBIA/PLK3/CYLD/NFATC1/BCL3/SRC/AKT3/  MDM2/CLEC7A | 22 | 0.26 | Activated |
| hsa04657 | IL-17 signaling pathway | 2.028132307 | 0.000149 | IL6/CXCL3/CXCL2/CXCL1/CCL20/CXCL6/PTGS2/CXCL8/CSF2/  IL1B/TNFAIP3/NFKB1/MUC5B/TNF/NFKBIA/CCL2/TRAF5/TRAF3 | 18 | 0.27 | Activated |
| hsa04740 | Olfactory transduction | -1.570743683 | 0.013205 | GNG7/ANO2/PDE2A/OR7D2/OR2A1/OR2T8/OR10AD1/OR52K1/  GNAL/CAMK2A | 10 | 0.27 | Suppressed |
| hsa04620 | Toll-like receptor signaling pathway | 1.945630026 | 0.000145 | IL6/IL12B/CXCL9/IFNB1/IRF9/MAP2K6/CD80/CXCL8/CCL4L2/IL1B/CCL4/CCL3L1/CCL3/NFKB1/MAP3K8/TLR3/CD40/TLR7/TNF/  NFKBIA/TICAM1/TLR8/TRAF3 | 23 | 0.28 | Activated |
| hsa00980 | Metabolism of xenobiotics by cytochrome P450 | -1.694311094 | 0.005952 | CYP3A5/GSTO2/DHDH/GSTM2/AKR7A3/ALDH3A1/AKR1C1 | 7 | 0.28 | Suppressed |
| hsa05321 | Inflammatory bowel disease | 1.821377774 | 0.000468 | IL18RAP/IL6/IL12B/IL1A/IL23A/IL18R1/STAT4/TBX21/IL1B/NFKB1/IL23R/IL10 | 12 | 0.29 | Activated |
| hsa05323 | Rheumatoid arthritis | 2.037271606 | 0.000149 | IL6/CXCL3/CXCL2/CXCL1/IL1A/CCL20/CXCL6/IL23A/CD80/IL15/  CXCL8/CSF2/IL1B/CCL3L1/CCL3/ICAM1/TEK/TNF/TNFSF11/CSF1/CCL2 | 21 | 0.32 | Activated |
| hsa04668 | TNF signaling pathway | 2.078751805 | 0.000139 | IL6/CXCL3/CXCL2/CXCL1/TRAF1/CCL20/CXCL6/IFNB1/VCAM1/MAP2K6/PTGS2/VEGFC/IL18R1/IL15/BIRC3/CSF2/IL1B/TNFAIP3/  SOCS3/LIF/NFKB1/MAP3K8/EDN1/ICAM1/CFLAR/TNF/NFKBIA/  CSF1/LTA/CCL2/TRAF5/FAS/TRAF3/MAP2K3/CREB3L1/JAG1/  CYLD | 37 | 0.34 | Activated |
| hsa04060 | Cytokine-cytokine receptor interaction | 1.949594382 | 0.000131 | IL18RAP/IL6/CXCL3/INHBA/CXCL2/IL12B/IL33/CXCL1/IL1A/  CXCL9/CCL20/CXCL6/IFNB1/IL23A/CXCL13/INHBB/CCR10/  IL18R1/IL15/TNFSF18/TNFRSF19/CXCL8/CCR5/CCL4L2/BMP6/  CSF2/IL1B/EBI3/CCL4/BMP2/CCL3L1/CXCL14/LIF/CCL3/CCL8/IL23R/IL34/TNFRSF11B/TNFRSF18/IL10/CD40LG/IL7R/CXCR5/  ACVR2A/PRLR/CD40/CXCR4/TNFRSF9/TNF/TNFSF11/CSF1/LTA/  ACKR3/CCL2/GDF11/BMPR1A/FAS/TNFSF9/CXCL12/CCR7 | 60 | 0.35 | Activated |
| hsa04064 | NF-kappa B signaling pathway | 1.906046872 | 0.000143 | CXCL3/CXCL2/CXCL1/TRAF1/VCAM1/PTGS2/CXCL8/BIRC3/CCL4L2/IL1B/CCL4/TNFAIP3/BLNK/NFKB1/CD40LG/ICAM1/NFKB2/  CD40/CFLAR/RELB/TNF/TNFSF11/NFKBIA/LTA/PLAU/TICAM1/  TRAF5/TRAF3/CXCL12/GADD45B/BCL2A1/CYLD/PIDD1/PRKCQ | 34 | 0.37 | Activated |
| hsa05144 | Malaria | 1.846823629 | 0.000318 | IL6/VCAM1/HBA2/CXCL8/IL1B/IL10/CD40LG/ICAM1/CD40/TNF/  CCL2/THBS4/SDC1 | 13 | 0.42 | Activated |
| hsa04979 | Cholesterol metabolism | -1.520599917 | 0.022558 | STARD3/NPC1/CD36/VAPB/LRP1/ABCA1/APOE/ABCB11/APOC1/  NCEH1/MYLIP/CETP/APOA1/APOA2/LIPC/ABCG8 | 16 | 0.43 | Suppressed |
| hsa04061 | Viral protein interaction with cytokine and cytokine receptor | 1.830923459 | 0.000299 | IL18RAP/IL6/CXCL3/CXCL2/CXCL1/CXCL9/CCL20/CXCL6/  CXCL13/CCR10/IL18R1/CXCL8/CCR5/CCL4L2/CCL4/CCL3L1/  CXCL14/CCL3/CCL8/IL34/IL10/CXCR5/CXCR4/TNF/CSF1/LTA/  ACKR3/CCL2 | 28 | 0.44 | Activated |
